# Chemically induced repair, adhesion, and recycling of polymers made by inverse vulcanization†
†Electronic supplementary information (ESI) available: Full experimental details, characterisation data and mechanistic discussion. See DOI: 10.1039/d0sc00855a


**DOI:** 10.1039/d0sc00855a

**Published:** 2020-05-15

**Authors:** Samuel J. Tonkin, Christopher T. Gibson, Jonathan A. Campbell, David A. Lewis, Amir Karton, Tom Hasell, Justin M. Chalker

**Affiliations:** a Institute for Nanoscale Science and Technology , College of Science and Engineering , Flinders University , Bedford Park , South Australia 5042 , Australia . Email: justin.chalker@flinders.edu.au; b Flinders Microscopy and Microanalysis , College of Science and Engineering , Flinders University , Bedford Park , South Australia 5042 , Australia; c School of Molecular Sciences , University of Western Australia , Perth , Western Australia 6009 , Australia; d Department of Chemistry , University of Liverpool , Liverpool L69 7ZD , UK

## Abstract

Inverse vulcanization is a copolymerization of elemental sulfur and alkenes that provides unique materials with high sulfur content (typically ≥50% sulfur by mass). These polymers contain a dynamic and reactive polysulfide network that creates many opportunities for processing, assembly, and repair that are not possible with traditional plastics, rubbers and thermosets. In this study, we demonstrate that two surfaces of these sulfur polymers can be chemically joined at room temperature through a phosphine or amine-catalyzed exchange of the S–S bonds in the polymer. When the nucleophile is pyridine or triethylamine, we show that S–S metathesis only occurs at room temperature for a sulfur rank > 2—an important discovery for the design of polymers made by inverse vulcanization. This mechanistic understanding of the S–S metathesis was further supported with small molecule crossover experiments in addition to computational studies. Applications of this chemistry in latent adhesives, additive manufacturing, polymer repair, and recycling are also presented.

## Abstract 2

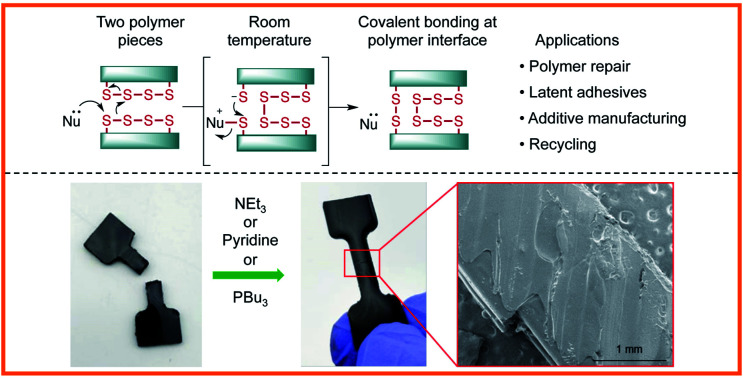
Polymers made by inverse vulcanization can be assembled, repaired, and recycled at room temperature through nucleophile-catalyzed S–S metathesis.

## Introduction

The introduction of inverse vulcanization by Pyun and co-workers has ushered in a new age of polymers with high sulfur content.^1^ In this process, elemental sulfur is copolymerized at a high feed ratio with an olefin, usually a diene or triene, to provide materials with ∼50–90% sulfur by mass—distinguishing these polymers from classic polysulfide polymers and rubbers.^2^ These unique polymers have found increasing use in energy storage applications, infrared optics, and environmental remediation.^3^–^6^ Additionally, these polymers are dynamic, with S–S bonds in the backbone that can be reversibly broken and reformed upon the application of heat or shear.^7^,^8^ This unique property of these polysulfide materials has prompted a number of studies on the thermally induced repair and recycling of these polymers.^9^–^17^ The ability to break the S–S bonds in these polymers has also been exploited in the insertion of monomers into the backbone of sulfur pre-polymers,^18^–^20^ delayed curing systems,^20^ and also in next-generation adhesives.^21^ In these studies, S–S bond cleavage is provoked by heating—usually to temperatures near 100 °C or higher. In one notable exception, Zhang has shown that sulfur polymer and liquid metal composites can self-heal at room temperature, but this adhesion is based on affinity of the sulfur for the metal rather than through S–S bond exchange.^22^ And while other innovative polymer systems have been reported that self-heal *via* S–S metathesis,^23^–^25^ polymers prepared by inverse vulcanization have the advantage of being prepared in a one-step process from very low cost building blocks, often on kilogram scale.^26^,^27^ Furthermore, the thermomechanical properties of polymers made by inverse vulcanization can be readily tuned through variation of the organic crosslinker and feed ratios of the monomers.^28^

**Fig. 1:**
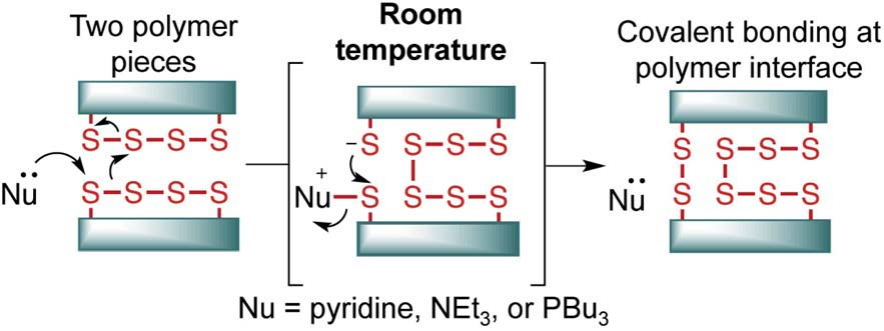
In this study, nucleophiles such as amines and tributylphosphine were tested in their ability to provoke S–S metathesis between the faces of polymers made by inverse vulcanization.

It is an important goal to develop methods for the adhesion and repair of polymers made by inverse vulcanization that do not require energy intensive heating. In this study, we report our progress toward this goal and reveal that nucleophiles such as amines or tributylphosphine can induce S–S metathesis in these polymers rapidly at room temperature, enabling facile adhesion, repair, and recycling (Fig. 1).

A number of clues in the literature suggest that nucleophiles such as amines could be used to break S–S bonds in polymers made by inverse vulcanization. Pyun, for instance, noted that the rate of inverse vulcanization was enhanced for aniline-derived monomers or when a nucleophile such as *N*-methylimidazole was added to the reaction.^29^,^30^ This phenomenon was attributed to the ability of the nucleophile to attack S_8_ and generate more reactive linear polysulfides.^29^,^30^ Similarly, Mutlu and co-workers have recently shown that amine base 1,5,7-triazabicyclo[4.4.0]dec-5-ene (TBD) can catalyze sulfur insertion and exchange reactions in related polymers.^31^ In our own studies, we have noted that pyridine was often the only solvent that could completely dissolve polymers made by inverse vulcanization.^26^,^32^–^34^ Rather than simply dissolving these polymers, we suspected that the pyridine actually broke S–S cross-links and converted the polymer into a new soluble species. It occurred to us that if this process were reversible, then we should be able to take advantage of this chemistry to induce S–S metathesis on two polymer surfaces, leading to covalent bonding between the polymer pieces (Fig. 1). Similarly, we hypothesized that nucleophilic phosphines would also induce the same S–S exchange on the polymer surface in the same way they do for disulfide metathesis reactions in small molecules.^35^ Notably, such polysulfide processing would be expected to proceed at room temperature, greatly reducing the energy input typically required to form and manipulate polymers made by inverse vulcanization.

## Results and discussion

### Polymer synthesis and characterization

**Fig. 2:**
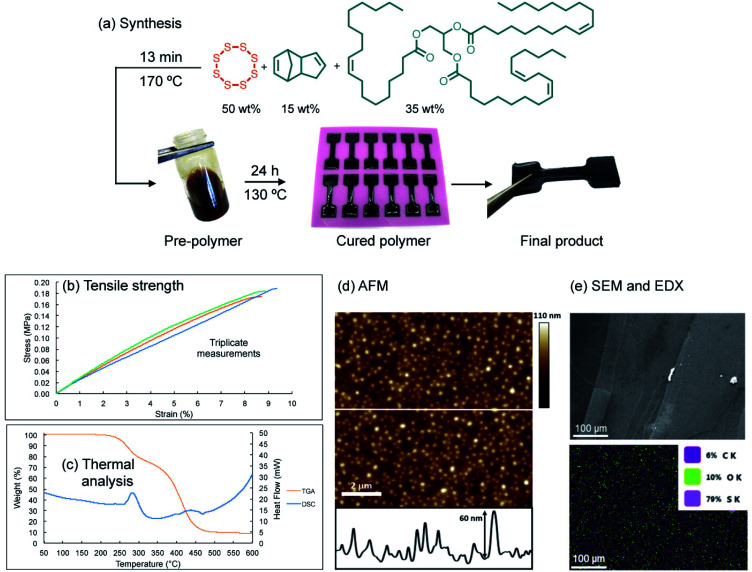
(a) Synthesis of a terpolymer by the direct reaction of sulfur, canola oil, and dicyclopentadiene. A liquid pre-polymer was prepared before molding and curing the polymer at 130 °C for 24 hours. (b) Tensile testing of dogbone samples. (c) Simultaneous thermal analysis of the polymer. (d) AFM analysis of surface roughness. The cross-section at the bottom of the image corresponds to the position of the white line in the AFM image. (e) SEM and EDX of polymer surface.

A model terpolymer made from the copolymerization of sulfur, canola oil (a triglyceride) and dicyclopentadiene (DCPD) was selected as a model system for this study. These polymers have previously been shown to display tunable elastomeric properties through variation of the feed ratio, which make them convenient for studying mechanical properties such as tensile strength and compression modulus.^28^ In the synthesis, sulfur (50 wt%) was melted in a vial and heated to 170 °C briefly before a mixture of canola oil (35 wt%) and DCPD (15 wt%) (pre-heated to 170 °C) was added to the reaction (ESI page S4†). After 13 minutes of heating with stirring, a dark liquid pre-polymer formed. This mixture was then poured into a silicone mold and then cured for 24 hours at 130 °C (Fig. 2a).

At this feed ratio, the average sulfur rank in the polymer is expected to be approximately 4. This parameter is based on the molar ratio of sulfur atoms to alkenes and the fact that all S_8_ was consumed in the reaction (determined by DSC) and 94% of the alkenes reacted (determined by ^1^H NMR) (see ESI page S4† for additional discussion). Additionally, we anticipated that this sulfur rank might be important in providing a relatively stable and robust bulk polymer, but one that contains S–S bonds weak enough to participate in metathesis (*vide infra*).

Physically, the cured polymer is a soft and flexible black rubber with a glass transition temperature of –9.1 °C, as determined by variable temperature dynamic mechanical analysis (DMA) and a compression modulus of 2.55 MPa (ESI page S5–S7†). The polymer, when molded in a dogbone shape (adapted from ISO 37 Type 4, Fig. 2a, ESI page S4 and S5†), could be stretched to 9 ± 0.27% strain before breaking for triplicate experiments, with a average measured tensile strength of 0.18 MPa (ESI page S6†). Simultaneous Thermal Analysis (STA) was used to obtain TGA and DSC data for the terpolymer (Fig. 2c). The DSC data did not reveal a phase transition between 100 °C and 150 °C, which is consistent with complete consumption of elemental sulfur in the polymerization. And while Raman spectroscopy revealed some residual elemental sulfur on the surface of the polymer, Raman spectroscopic analysis of a polymer cross-section did not show any unreacted sulfur (ESI page S12 and S13†). We attribute this minor amount of sulfur on the surface as contamination from sublimed sulfur generated during the polymer curing. Thermal gravimetric analysis (TGA) indicated two mass losses for the polymer. The first onset occurred at just over 200 °C, corresponding to S–S decomposition and extrusion of sulfurous material, with the remaining organic material decomposing above 350 °C. Such TGA profiles are consistent with previous thermal analyses on related copolymers.^32^,^36^ The polymer was smooth in appearance, and indeed surface analysis by scanning electron microscopy revealed a continuous bulk polymer with no visible crystals of sulfur. Atomic force microscopy (AFM) analysis also revealed a smooth polymer with an average surface roughness of 8.84 ± 1.28 nm.^37^ It is not clear if this surface feature is a consequence of some type of phase separation or morphological variation imparted by the curing process, but surface roughness is nonetheless an important parameter in studies of polymer adhesion so it is reported here (Fig. 2d and e).

Additional characterization by infrared (IR) spectroscopy revealed (sp^3^) C–H signals at 2923 and 2851 cm^–1^ (expected for the hydrocarbon co-monomers), as well as the C=O stretch from the canola oil triglyceride at 1742 cm^–1^ (ESI page S14†). ^1^H NMR spectroscopy in deuterated pyridine indicated complete consumption of the norbornene alkene signal of DCPD at *δ* = 5.98 ppm, and a combined 94% conversion of the remaining alkenes in the canola oil and the cyclopentene group of DCPD. This analysis was based on the change in integration of these alkene signals relative to the methine signal in the triglyceride over the polymerization (ESI page S15–S17†).

### Polymer repair and mechanistic studies

With the model polymer in hand, as well as initial tensile strength benchmarks, we set out to assess pyridine and tributylphosphine as chemical inducers for polymer repair. Accordingly, the dogbone molds were cut through the gage section using a scalpel. The two pieces were then placed back into the mold and pyridine or tributylphosphine (1–15 µL) was applied to the cut surface (4 × 2 mm). The cut pieces were left in contact without applied pressure for 24 hours. Both pyridine and tributylphosphine provoked repair of the polymer, with 10 µL of pyridine and 1 µL of tributylphosphine leading to the most promising recovery, returning to 60% and 45% of the tensile strength in these first tests, respectively (ESI page S18 and S19†).

**Fig. 3:**
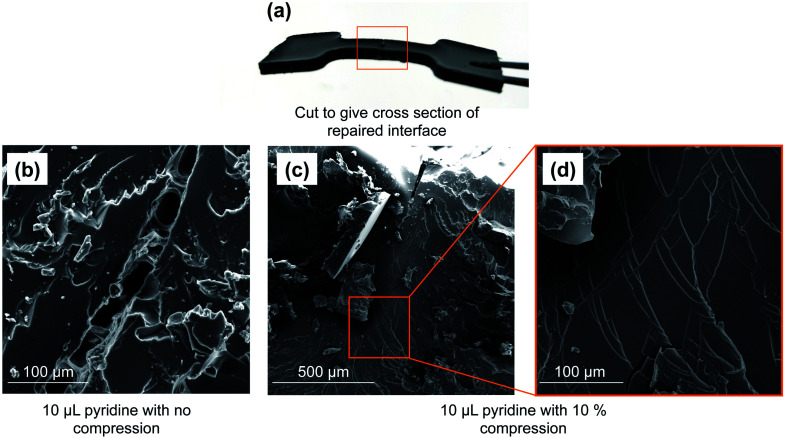
(a) Image of repaired polymer. (b) Application of 10 µL of pyridine to a cut polymer interface leads to partial repair when no compression is applied. (c) When pressure is applied to force polymer interfaces together (10% compression, orthogonal to the bonding interfaces), 10 µL of pyridine catalyzes the repair of the polymer. (d) The repaired region of the polymer presents as continuous bulk material.

SEM analysis of the polymer interface clearly revealed regions of repair, but the repair was not uniform (Fig. 3). By simply compressing these pieces together (10% compression orthogonal to the bonding interface), the reacting interfaces were in more uniform contact and led to more thorough repair. In this case, 74% of the original tensile strength was recovered in the case of pyridine. Importantly, no adhesion was observed in a negative control in which neither pyridine nor tributylphosphine were used. When common solvents such as acetone, chloroform, ethanol, toluene, THF or DMF were applied to the polymer interface (instead of pyridine or tributylphosphine), there was no polymer repair (ESI page S20–S24†). This result means that the repair mechanism is not simply polymer dissolution and re-entanglement of polymer chains, as might be possible with a linear polymer. Rather, the repair is the result S–S metathesis, catalyzed by pyridine or tributylphosphine (*vide infra*). Additionally, analyzing the tensile strength of the dogbone after different times of repair revealed that less than 1 hour was required to reach maximum strength for tributylphosphine induced repair and 2 hours for pyridine (ESI page S19†). Therefore, the chemically-induced adhesion between the polymer pieces is relatively rapid at room temperature.

Investigating the mechanism of repair further, energy-dispersive X-ray (EDX) spectroscopy was used to map the elemental composition of the repaired polymer interface. In the case of tributylphosphine, phosphorous was detected throughout the bulk polymer in repaired cross-sections (ESI page S25 and S26†). This result suggests that the phosphine is diffusing through the polymer as it is reacting and provoking S–S metathesis. In contrast, pyridine was not detected, likely because it evaporated under the reduced pressure used in the measurement. This result implied that the pyridine could be removed after inducing repair and it was therefore traceless. Investigating this hypothesis further, tributylphosphine and pyridine were each applied directly to the surface of an undamaged polymer, incubated for 24 hours, and then extracted with chloroform for analysis (ESI page S27–S33†). It was found by GC-MS and NMR analysis that the tributylphosphine was completely reacted and converted primarily to tributylphosphine sulfide. This product could be completely extracted from the polymer, as no phosphorous was detected in the polymer by EDX analysis after the extraction. Pyridine, however, could be extracted—unreacted—into chloroform after 24 hours on the polymer. This result confirms that pyridine is not converted into another compound when in contact with the polymer. This is consistent with pyridine acting as a nucleophilic catalyst for the S–S metathesis at the polymer surfaces.

**Fig. 4:**
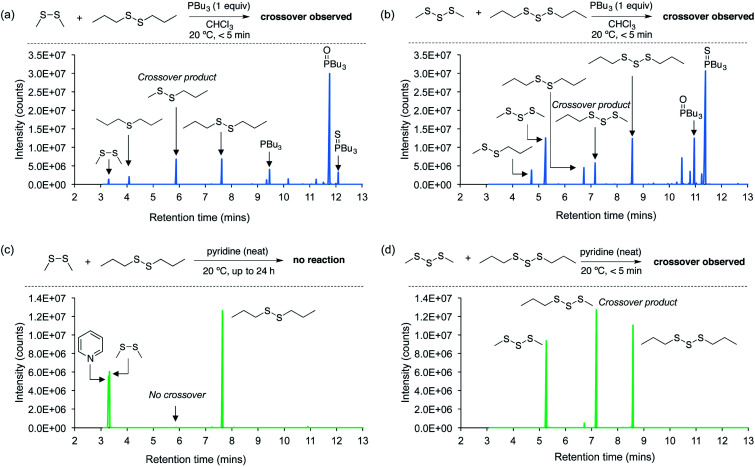
GC-MS analysis of crossover experiments using model disulfides and trisulfides and their reaction with tributylphosphine or pyridine. (a) Tributylphosphine mediates rapid S–S metathesis and desulfurization of dimethyl disulfide and di-*n*-propyl disulfide at 20 °C. (b) Tributylphosphine mediates rapid S–S metathesis and desulfurization of dimethyl trisulfide and di-*n*-propyl trisulfide at 20 °C. (c) Pyridine does not react with dimethyl disulfide or di-*n*-propyl disulfide at 20 °C. (d) S–S metathesis of dimethyl trisulfide and di-*n*-propyl trisulfide occurs rapidly at 20 °C when the trisulfides are reacted in neat pyridine.

Next, a series of crossover experiments on model disulfides and trisulfides were carried out to further confirm that both tributylphosphine and pyridine can indeed mediate the S–S metathesis mechanism proposed for the polymer adhesion (Fig. 4 and ESI page S33–S43†). Accordingly, equimolar amounts of dimethyl disulfide, di-*n*-propyl disulfide, and either tributylphosphine or pyridine were added to chloroform so that the final concentration of all three components was 115 mM. Tributylphosphine led to rapid S–S metathesis as well as desulfurization within minutes at 20 °C (see ESI page S36† for a mechanistic discussion).^38^,^39^ A similar outcome was observed in the reaction of tributylphosphine with dimethyl trisulfide and di-*n*-propyl trisulfide (Fig. 4b). In contrast, pyridine did not react with the disulfides under these conditions. Even when the disulfides were dissolved directly in pyridine and incubated for 24 hours at 20 °C, no crossover products were detected (Fig. 4c). This was initially surprising given the adhesion results for the polymer experiments. However, we suspected that higher sulfur rank materials with weaker S–S bonds, such as trisulfides, might be required for reaction with pyridine. Indeed, when dimethyl trisulfide and di-*n*-propyl trisulfide were dissolved in pyridine, rapid and clean S–S metathesis was observed within 5 minutes by GC-MS (Fig. 4d), providing direct evidence that pyridine can indeed provoke the reaction required for polymer repair.

It should be noted that neat pyridine was required for the crossover of the trisulfide substrates, and when equimolar amounts of pyridine and the trisulfides were prepared as a 115 mM solution in chloroform, no reaction was observed (ESI page S42†). Because the pyridine is applied directly to the polysulfide polymer, we regard the reaction in neat pyridine as a better model for the polymer repair mechanism.

The crossover experiments were further corroborated computationally. Accordingly, high-level *ab initio* calculations (using the *ab initio* G4(MP2) thermochemical protocol)^40^,^41^ were performed in a simulated pyridine environment (SMD(pyridine)-G4(MP2)) in order to gain mechanistic insights into the different reactivity of the RSSR and RSSSR sulfur chains with pyridine. Full computational details are given in the ESI (ESI page S44–S51).† The G4(MP2) protocol is an efficient composite procedure for approximating the CCSD(T) energy (coupled cluster with singles, doubles, and quasiperturbative triple excitations) in conjunction with a large triple-ζ-quality basis set and has been found to produce thermochemical and kinetic properties with chemical accuracy (arbitrarily defined as ∼4 kJ mol^–1^).^40^–^45^ For reasons of computational cost, the R groups were modeled as methyl groups. We found that these reactions proceeded *via* two consecutive nucleophilic attacks. In the first step, the pyridine nitrogen attacks the terminal sulfur of MeS_*n*_Me (*n* = 2, 3) to form PySMe^+^ and MeS_(*n*–1)_^–^. The MeS_(*n*–1)_^–^ species generated is only needed in a catalytic amount in order to initiate subsequent S–S metathesis reactions (*e.g.* the crossover reaction in Fig. 4 or the polymer interfacial adhesion reaction).

We found that the reaction of pyridine with MeS_3_Me is kinetically favored by as much as 43.1 kJ mol^–1^ over the reaction with MeS_2_Me; which, according to the Eyring equation, translates into a difference of ∼7 orders of magnitude in the reaction rates at 298 K. In addition, the reaction with MeS_3_Me is less endergonic by 34.0 kJ mol^–1^ than the reaction with MeS_2_Me. Thus, the reaction of pyridine with MeS_3_Me is both kinetically and thermodynamically favored over the reaction with MeS_2_Me. This computational result is consistent with the crossover experiments in Fig. 4 in which pyridine reacted with the trisulfides but not the disulfides. The catalytic MeS_(*n*–1)_^–^ species generated in the first step can then nucleophilically attack the terminal sulfur atom of a neutral MeS_*n*_Me molecule (*n* = 2, 3). This second nucleophilic attack is found to proceed *via* a low-lying transition state with activation energies of Δ*G*‡298 = 49.6 (MeS^–^ + MeS_2_Me), 61.6 (MeS_2_^–^ + MeS_2_Me), 34.6 (MeS^–^ + MeS_3_Me), and 47.7 (MeS_2_^–^ + MeS_3_Me) kJ mol^–1^ relative to the free reactants. Taken together, these computational results indicate that only a catalytic amount of the strong MeS_*n*_^–^ nucleophile is required to initiate S–S metathesis, but pyridine can only generate such species at room temperature for dialkyl trisulfides and species of higher sulfur rank, but not for dialkyl disulfides.

**Fig. 5:**
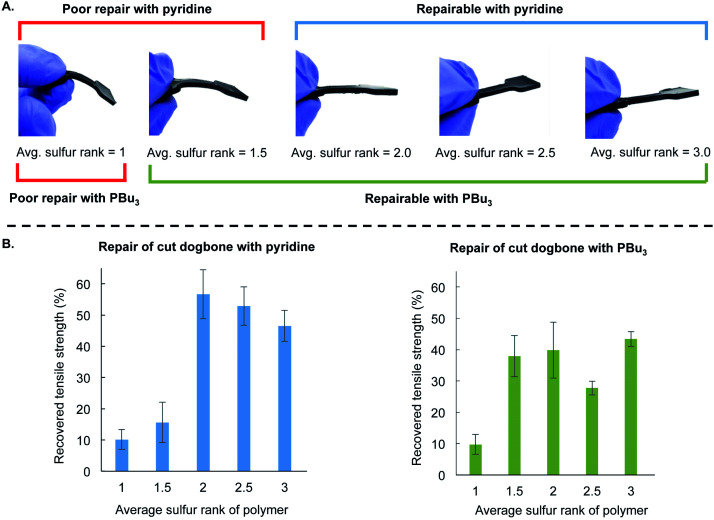
(A) Polymer dogbones were prepared with different feed ratios of sulfur to provide materials with different average sulfur rank. The ratio of DCPD to canola oil was kept constant for all of these polymers. The polymers were prepared in a silicone mold and cured for 24 hours at 130 °C. The amount of sulfur in the polymers influenced the flexibility of the final product, with lower sulfur rank resulting in a more flexible material and higher sulfur rank resulting in a more rigid material. (B) The dogbone samples were cut and then repaired by the application of pyridine (10 µL) or tributylphosphine (10 µL) to the surfaces of the cut dogbones and incubating in the mold for 24 hours at room temperature. No compression was applied during the repair and all experiments were performed in triplicate. The tensile strength was tested and compared to an undamaged polymer of the same sulfur rank. Poor repair was observed when pyridine was applied to the polymers with an average sulfur rank of 1 or 1.5. Poor repair was observed when tributylphosphine was applied to the polymer with an average sulfur rank of 1. The polymers with an average sulfur rank of 2 or more could be repaired with either pyridine or tributylphosphine.

This mechanistic framework was also consistent with repair of polymer dogbones made with varying sulfur rank (Fig. 5). These materials were made using the same procedure as before by preparing the liquid pre-polymer and then curing in the silicone mold at 130 °C for 24 hours. The ratio of canola oil to DCPD was kept constant for all of these polymers, and they differ only in the amount of sulfur used in the reaction. The average sulfur rank of these polymers was 1, 1.5, 2.0, 2.5, and 3.0. In Fig. 5A, it is clear that the mechanical properties of these polymers differ, with the lower sulfur rank material having greater flexibility and the materials with higher sulfur rank were more rigid (see ESI page S52–S56† for additional characterization). This is another example in which the material properties of polymers made by inverse vulcanization can be controlled by simple variation of the feed ratio of the monomers in these terpolymers.^28^ These dogbones were then cut and repaired by the application of either 10 µL pyridine or 10 µL tributylphosphine. Tensile strength was then measured and compared to the strength of the non-cut dogbone (Fig. 5B and ESI page S56†). Poor repair was observed when pyridine was applied to the polymers with average sulfur ranks of 1 and 1.5. Repair was observed for the polymers with average sulfur rank of 2 or more. For tributylphosphine, poor repair was observed when applied to the polymer with an average sulfur rank of 1, but repair could be induced at higher sulfur ranks (Fig. 5B). These results are consistent with the model studies and computational results that predict the reaction of pyridine and disulfides to be slow or not occur at room temperature. Pyridine-catalyzed repair of the polymer with an average sulfur rank of 2 likely occurred because of reaction of pyridine with trisulfide linkages or longer sulfur chains in the polymer (the sulfur rank is only an average, so a sulfur rank of 2 will contain a distribution of longer and shorter sulfur chains). The more nucleophilic tributylphosphine, in contrast, can induce the repair of the lower sulfur rank material (sulfur rank 1.5) because it can react with the disulfide linkages in this material.

**Fig. 6:**
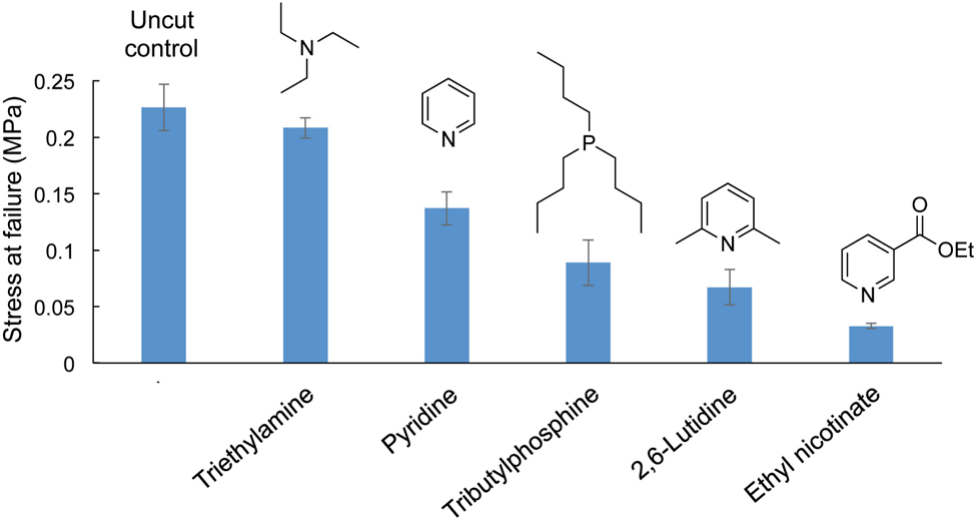
Liquid catalysts (10 µL) used to repair cut dogbone polymers made by inverse vulcanization (50 wt% sulfur, 35 wt% canola oil, 15 wt% DCPD, sulfur rank ∼ 4). Repair with triethylamine returned the dogbone sample to its original tensile strength. All experiments were performed in triplicate.

Different amine catalyst were tested next and compared to the original pyridine and tributylphosphine catalysts in the repair of cut dogbone samples (Fig. 6 and ESI page S57†). Ethyl nicotinate (an ester of vitamin B3 and pyridine derivative) was tested because it is a less noxious alternative to pyridine. However, the tensile strength of the repaired polymer was poor, likely because the nucleophilicity is reduced because of the electron-withdrawing ester group. Similarly, 2,6-lutidine was tested, but the tensile strength of the repaired polymer was again weaker than obtained with repair with pyridine. The methyl groups likely hinder nucleophilic attack of the catalyst on S–S bonds, thereby slowing the initial step of S–S metathesis. In contrast, triethylamine performed well in repair, restoring the polymer dogbone to its original tensile strength. In fact, when testing the polymer repaired with triethylamine, the failure did not occur at the repaired interface but at a different position in the dogbone. We attribute the rapid and effective repair to triethylamine's greater nucleophilicity compared to pyridine. Triethylamine can therefore initiate the S–S metathesis faster than the other catalysts. This greater reactivity was also observed in a cross-over experiment with dimethyl trisulfide and di-*n*-propyl trisulfide. Unlike pyridine, neat triethylamine was not required to catalyze the cross-over reaction. Triethylamine could catalyze this reaction in chloroform at a concentration of 115 mM, while pyridine, 2,6-lutidine, and ethyl nicotinate were unreactive under these conditions. Triethylamine did not catalyze the analogous cross-over experiment between dimethyl disulfide ad di-*n*-propyl disulfide, so this catalyst (like pyridine) is most effective on substrates with a sulfur rank of 3 or higher. Full details of these crossover experiments are provided in the ESI (ESI page S58–S62).†

The assessment of repair of polymers with varying sulfur rank and the tests with different nucleophilic catalysts revealed that S–S bond metathesis is an important step in the repair mechanism. Next, the affect of the glass transition temperature on repair was investigated because the repair might only occur when the polymer domains at the interface can flow after S–S bond cleavage. If this were the case, then the repair would need to be carried out above the *T*_g_. To test this hypothesis, a polymer was prepared with a higher feed ratio of DCPD, as we have previously shown that there is often a linear correlation between the DCPD feed ratio and glass transition temperature for this class of polymers.^28^ Accordingly, inverse vulcanization was used to prepare a polymer made of 50 wt% sulfur, 15 wt% canola oil, and 35 wt% DCPD (ESI page S63†). At this composition, the average sulfur rank is 2.7. The *T*_g_ for this material was 60 °C, as determined by DSC (ESI page S64†). This polymer was very brittle and had a much higher tensile modulus (1092 MPa) than the polymer made from 50 wt% sulfur, 35 wt% canola oil, and 15 wt% DCPD (2 MPa) (ESI page S64†). When dogbone molds of this higher *T*_g_ polymer were cut and repair was attempted by application of either pyridine or tributylphosphine (10 µL), no repair was observed after 24 hours and the polymer remained in two pieces. This result suggests that *T*_g_ is indeed an important factor in the polymer repair mechanism.

These computational and mechanistic studies are enlightening in several respects. First, crossover experiments provided direct evidence that pyridine can break S–S bonds in polysulfides. This result explains why pyridine can “dissolve” many polymers made by inverse vulcanization: the S–S crosslinks are cleaved and break the polymer network into soluble fragments. The crossover experiments and computational results also revealed that the weakly nucleophilic pyridine requires a sulfur rank > 2 to provoke S–S metathesis, so the repair of the polymers will likely only be catalyzed by pyridine at room temperature when the polymer contains sulfur chains with at least 3 sulfur atoms. For the terpolymer prepared in Fig. 2, the feed ratios correspond to an average sulfur rank of approximately 4 (see ESI page S4†), so the S–S metathesis and polymer repair is consistent with the model crossover experiment in Fig. 4 and the computational considerations. The more nucleophilic phosphine, however, can rapidly induce S–S metathesis of disulfides so this nucleophile is the more appropriate reagent for adhesion and repair of polysulfide polymers with a sulfur rank of 2. These conclusions are also consistent with the repair of polymer with varying sulfur rank shown in Fig. 5. Finally, the variation in catalyst effectiveness between ethyl nicotinate, 2,6-lutidine, pyridine and triethyl amine is also consistent with a polymer repair mechanism that requires S–S bond cleavage. As mentioned above, ethyl nicotinate is electron poor compared to pyridine and therefore less nucleophilic. Similarly, 2,6-lutidine is less nucleophilic because of the steric hindrance near the nitrogen lone pair. Triethylamine, in contrast, is more nucleophilic than pyridine, consistent with the more rapid cross-over in the trisulfide models and the more effective repair in the polymer dogbone. Finally, it was found that repair was not observed when the catalyst were applied to the material below the glass transition temperature.

Taking into account this data, we propose that the mechanism of polymer repair proceeds through S–S metathesis reactions initiated by nucleophilic attack by the catalyst and cleavage of S–S bonds. Breaking the S–S bonds enables disentanglement and reorganization of the polymer network at the interface when the repair is carried out above the glass transition temperature. Solvation alone cannot account for the repair, because no repair is observed if common solvents such as acetone, chloroform, ethanol, toluene, THF or DMF are added to the polymer interface. Furthermore, polymers with an average sulfur rank < 1.5 were not repairable with pyridine or tributylphosphine. Repair is only observed when the liquid applied to the polymer can cleave S–S bonds, which requires an appropriate sulfur rank (Fig. 5) and a sufficiently potent nucleophilic catalyst (Fig. 6). The repair process ends when the amine catalyst is regenerated and lost to evaporation. For phosphines, the catalyst ultimately decomposes to the corresponding sulfide. Additional discussion of this mechanistic proposal is found in the ESI (ESI page S66 and S67†).

### Latent adhesives, additive manufacturing, and polymer recycling

**Fig. 7:**
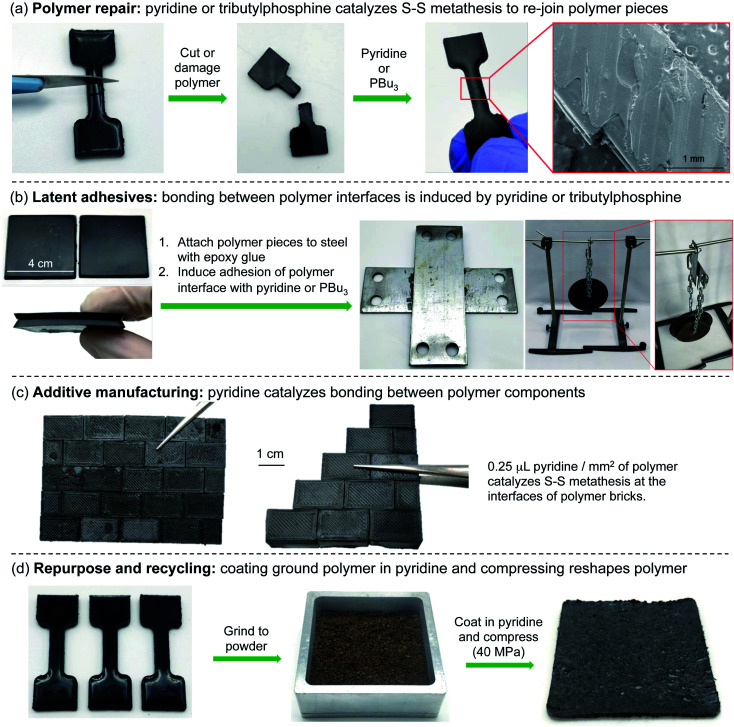
Applications enabled by pyridine or phosphine catalyzed S–S metathesis on polymers made by inverse vulcanization. (A) Polymer repair. (B) Latent adhesives. (C) Additive manufacturing. (D) Polymer repurposing and recycling.

With a mechanistic model of the polymer repair established, we next set out to explore the contexts in which this reaction might be useful in processing polymers made by inverse vulcanization. In addition to polymer repair (Fig. 3 and 7a), adhesion, additive manufacturing, and recycling were also examined. For adhesion, two 4 cm^2^ polymer pieces were attached to metal adherends using a commercial epoxy glue. Then, the polymer faces were bonded together by the application of 200 µL of tributylphosphine or pyridine. Pyridine was selected as the amine in these applications, because of our extensive preliminary work and computational studies with this catalyst. However, we note that triethylamine could also be used for these applications based on the dogbone repair studies described in Fig. 6. After 24 hours of induced adhesion at room temperature, the joined polymer pieces were tested in in-house shear and peel tests (Fig. 7b and ESI page S68–S70†). In triplicate experiments, the pyridine-bonded interface could support an average of 22 kg of weight before failure in the shear tests, corresponding to a shear strength of 12 N cm^–2^. In the peel test, the polymer supported an average of 11 kg of weight before failure, corresponding to an adhesive strength of 25 kg cm^–1^. Similarly, the polymer bonded by treatment with tributylphosphine could support an average of 21 kg of weight before failure in the shear tests (shear strength of 11 N cm^–2^) and 8 kg of weight in the peel tests (adhesive strength of 16 kg cm^–1^).

While the formulation of the polymer is not optimized for shear or adhesive strength, it should be noted that this is a promising demonstration of using these sulfur polymers as latent adhesives, where the bonding is provoked chemically by a catalyst such as a phosphine or an amine such as pyridine. This chemically induced bonding also has potential in additive manufacturing and assembly. For instance, polymer components could be molded separately and then assembled into chemically bonded objects. In the demonstration of this concept, pyridine was used as a “catalytic mortar” in the assembly of wall of polymer bricks (Fig. 7c and ESI page S71†). In this assembly, 0.25 µL of pyridine per mm^2^ of polymer was used to bond the bricks together. This amount of pyridine is unoptimised and was selected so that a thin film of pyridine covered the entire polymer surface. However, it should be noted that less pyridine can be used if it is applied as a solution in chloroform to the polymer interface, as demonstrated in the repair of cut dogbone samples (ESI page S72†).

Finally, pyridine was examined as a catalyst for polymer recycling and repurposing (Fig. 7d and ESI page S73–S75†). In the event, several polymer dogbone molds were ground into a crumb and passed through a 1 mm sieve to obtain relatively uniform particles. Next, 10 g of the polymer powder was dip-coated with pyridine before compressing at 20 °C for 30 minutes at 40 MPa in a hydraulic press. This process resulted in the formation of a rubber mat. A control experiment in which pyridine was omitted did not result in the formation of a mat, indicating the pyridine catalyst is required for this reactive processing (ESI page S75†). This polymer recycling and repurposing technique is distinct from most other recycling methods because the material is not simply heated and reshaped. Instead, the polysulfide groups on the polymer surface react in the presence of pyridine at room temperature and undergo S–S metathesis to join together the polymer pieces into a new shape.

## Conclusions

Chemically-induced repair, adhesion, and recycling of polymers made by inverse vulcanization was demonstrated at room temperature for the first time. Tributylphosphine was consumed in the reaction, but its high nucleophilicity led to rapid S–S metathesis between polymer faces. Pyridine and triethylamine, in contrast, were catalysts for the S–S metathesis, provided that the sulfur rank was >2. Understanding the fundamental mechanisms of this chemistry opens up new opportunities for processing polysulfide polymers in additive manufacturing, recycling, and latent adhesives. We are currently pursuing these leads in industrial contexts.

## Conflicts of interest

There are no conflicts of interest to declare.

## Supplementary Material

Supplementary information
