# Supplementary material for: Chemically induced repair, adhesion, and recycling of polymers made by inverse vulcanization
Source: Chem Sci. 2020 May 15;11(21):5537–46. doi: 10.1039/d0sc00855a (PMC7441575; doi:10.1039/d0sc00855a)
Supplement: Supplementary file 1 [file SC-011-D0SC00855A-s001.pdf]

Electronic Supplementary Material (ESI) for Chemical Science.
This journal is © The Royal Society of Chemistry 2020

Electronic Supplementary Information

Chemically induced repair, adhesion, and recycling of polymers made by inverse vulcanization

Samuel J. Tonkin,[†] Christopher T. Gibson,[‡] Jonathan A. Campbell,[†] David A. Lewis,[†] Amir Karton,[§] Tom Hasell,[¶] Justin M. Chalker*,[†]

[†] Institute for Nanoscale Science and Technology, College of Science and Engineering, Flinders University, Bedford Park, South Australia 5042, Australia.

[‡] Flinders Microscopy and Microanalysis, College of Science and Engineering, Flinders University, Bedford Park, South Australia 5042, Australia.

[§] School of Molecular Sciences, University of Western Australia, Perth, Western Australia 6009, Australia

[¶] Department of Chemistry, University of Liverpool, Liverpool L69 7ZD, United Kingdom

E-mail: justin.chalker@flinders.edu.au

Table of Contents

Canola oil composition	S2
Dogbone mold design and manufacture	S3
Polymer synthesis	S4
Glass transition temperature by dynamic mechanical analysis	S5
Tensile testing of polymer	S6
Compression testing of polymer	S7
SEM and EDX characterization of polymer	S8
AFM analysis of the polymer surface	S10
Thermal analysis of polymer (TGA and DSC)	S11
Raman spectroscopy characterization of polymer	S12
IR characterization of polymer	S14
NMR characterization of polymer	S15
Repair of dogbone sample using pyridine and tributylphosphine	S18
Polymer solubility and adhesion controls using conventional solvents	S22
SEM and EDX of repaired polymer interface	S25
Mechanistic study of polysulfide metathesis	S27
Extracted products formed after treating polymer with tributylphosphine or pyridine (GC-MS)	S27
EDX of polymer treated with pyridine or tributylphosphine (before and after chloroform wash)	S29
NMR of products formed when polymer treated with pyridine or tributylphosphine	S32
Disulfide crossover experiments using pyridine and tribuytlphosphine catalysts	S33
Trisulfide crossover experiments using pyridine and tributylphosphine catalysts	S39
Computational study of polysulfide metathesis using pyridine as a catalyst	S44
Repair of polymers with varying sulfur rank (1, 1.5, 2, 2.5, 3)	S52
Repair of polymers using triethylamine, 2,6-lutidine, and ethyl nicotinate	S57
Disulfide crossover experiments using triethylamine, 2,6-lutidine, and ethyl nicotinate catalysts	S58
Trisulfide crossover experiments using triethylamine, 2,6-lutidine, and ethyl nicotinate catalysts	S60
Synthesis and attempted repair of a polymer with a higher glass transition temperature (T_g = 60 °C)	S63
Mechanistic proposal for polymer repair with tributylphosphine	S66
Mechanistic proposal for polymer repair with pyridine	S67
Shear and peel tests after using the polymer as a latent adhesive	S68
Polymer assembly and additive manufacturing demonstration	S71
Repair of polymers using solution of pyridine in chloroform	S72
Polymer recycling and reforming using a pyridine catalyst	S73
References	S76

Canola oil composition

The composition of the canola oil used in the key polymerization was recently reported in a separate study by our lab.[1] Briefly, the canola oil triglyceride was reacted with sodium methoxide in methanol to convert the fatty acids to their corresponding methyl esters via transesterification. The resulting products were extracted and analyzed by GC-MS to identify the relative amounts of each fatty acid in the canola oil triglyceride. The results are summarized below:[1]

**Compound (fatty acid methyl ester)**		**Canola oil (% content)**
Saturated fatty acids	Tridecylic acid C13:0	-
	Palmitic acid C16:0	3.82
	Stearic acid C18:0	2.07
	Arachidic acid C20:0	0.49
Mono-unsaturated fatty acids	Palmitoleic acid C16:1 (9)	0.02
	Oleic acid C18:1 (9)	73.79
	Gadoleic acid C20:1 (9)	-
	Gondoic acid C20:1 (11)	0.92
Poly-unsaturated fatty acids	Linoleic acid C18:2 (9,12)	18.24
	α-Linolenic acid C18:3 (9,12,15)	0.65
Mono-unsaturated fatty acids with hydroxyl group	Ricinoleic acid C18:1 (9Z, 12-OH)	-
	Ricinelaidic acid C18:1 (9E, 12-OH)	-
	Total	100%

From the table above, the average molar mass of the canola oil can be calculated, weighting each fatty acid component by its relative amount.

Average canola oil molar mass = 882.6 g/mol

Average number of alkenes in canola oil = 3.4 alkenes / molecule canola oil

Dogbone mold design and manufacture

For mechanical testing of the polymer, controlling the size and shape are important for consistent results. Molding the polymer (rather than cutting it out of a polymer sheet) provided the most reliable access to uniform dogbone samples for mechanical testing. The mold was designed such that 12 pieces can be prepared in parallel. The molds were produced by 3D printing a mold negative in which a heat resistant silicone can be poured to produce a mold. A Creality Cr-10s Pro 3D printer was used with a poly(lactic acid) filament. Standard poly(lactic acid) printing settings were used with a nozzle temperature of 195 °C and a bed temperature of 60 °C. The 3D printed mold negative was designed using Autodesk Fusion 360 with dimensions shown in the figure below. Ultimaker Cura 4.0.0 was used to splice the model with 20% infil, a layer thickness of 0.1 mm (fine) and a wall thickness of 1 mm. 12 of the dogbone shapes of the stated dimensions were placed in a six by two formation with a four millimetre gap between each both vertically and horizontally. Each dogbone shape was printed to a depth of 2 mm on a base extending 10 mm on each side of the six by two grid with a depth of 2 mm. An offset of 2 mm along all outer sides of the base was used to raise a wall to a height of 4 mm. This wall was designed such that when liquid silicon was poured into the mold negative, it could be filled to the top of the wall such that it will cover all dogbone shapes and the molds will be produced to a consistent depth. The silicone used was pinkysil, produced by Barnes. When mixed, the silicon can be processed as a liquid for six minutes before it will begin to set to become a soft, shape persistent polymer. The mold was set for at least two hours before use.

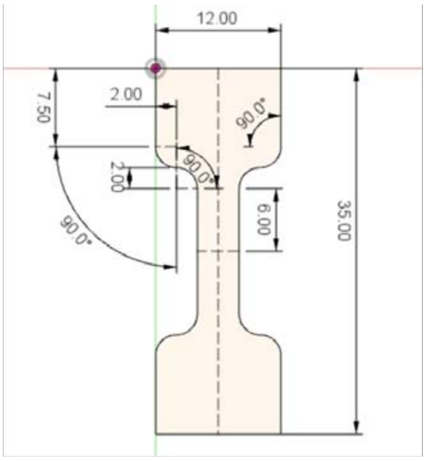

Dog bone design sketch
(dimensions in mm)

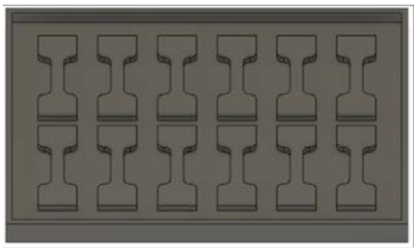

3D mold negative model

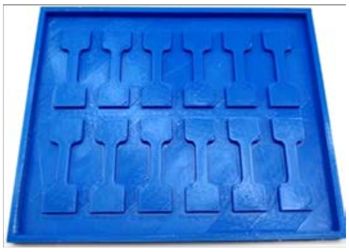

3D printed mold negative

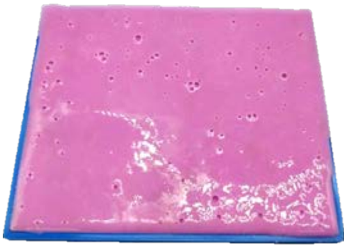

Silicone mixture in mold negative

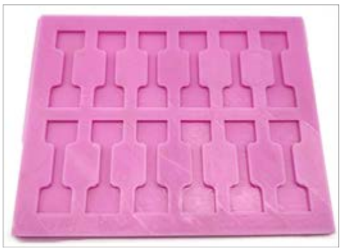

Final silicone mold

Polymer synthesis

The polymer was prepared by the copolymerization of sulfur, dicyclopentadiene (DCPD) and canola oil. The mass ratio of the monomers was 50% sulfur, 15% DCPD and 35% canola oil. The sulfur (5.00 g, 156 mmol) was added to a 25 mL glass vial with a magnetic stirrer. The sulfur was then heated at 170 °C for 2.5 minutes on an aluminium hot block to melt the sulfur and initiate ring-opening polymerisation. Constant stirring at 400 rpm was maintained during heating. Separately, canola oil (3.50 g, 3.97 mmol) and DCPD (1.50 g, 11.3 mmol) were added to a second 25 mL glass vial. The canola oil and DCPD mixture was heated at 170 °C for 30 seconds to provide a homogenous liquid mixture. The canola oil and DCPD were then poured into the glass vial containing the sulfur while heating and stirring were maintained. The reaction was heated at 170 °C for an additional 13 minutes. This time was found to be the point at which the pre-polymer appeared as a homogeneous dark liquid. Note that insufficient reaction time results in unreacted monomers (sulfur and alkenes) that are visible in the heterogeneous reaction mixture. Longer heating time can result in premature vitrification of the polymer mixture. After the 13 minutes of heating, the pre-polymer liquid was poured directly into a silicon mold. The mold with polymer was then added to a preheated oven at 130 °C and cured for 24 hours. After this time, the mold was removed from the oven and allowed cooled to room temperature before polymer was removed.

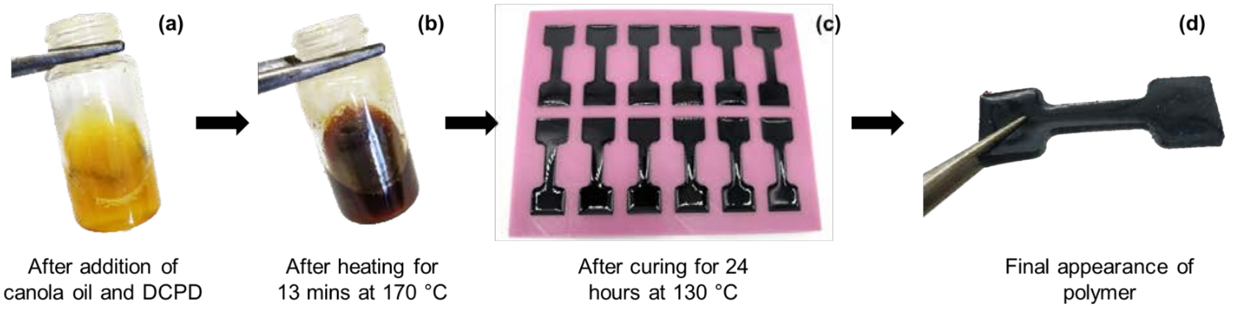

Based on the feed ratios, the molar ratio of sulfur : canola oil : DCPD = 156 : 3.97 : 11.3.

DCPD has 2 alkenes per molecule and canola oil has an average of 3.4 alkenes per molecule. At the feed ratios in the reaction, there are 22.6 mmol of alkenes from the DCPD and 13.5 mmol of alkenes from the canola oil (total of 36.1 mmol of alkenes in the reaction). Because there is 156 mmol of sulfur, there is an average of 4.3 sulfur atoms per alkene. The sulfur rank in the polymer is therefore 4.3.

Glass transition temperature using dynamic mechanical analysis

A dynamic mechanical analyzer (TA Instruments Q800) was used to determine the glass transition temperature of the polymer. This was achieved using the film tension clamp and the DMA multi-frequency strain setting. A 10 mm x 25 mm x 2.5 mm rectangular piece of polymer (cut from a polymer molded into a 40 x 40 x 2.5 mm square piece) was added to the DMA clamp. The polymer piece was then oscillated with an amplitude of 5 µm and a frequency of 1 Hz. The temperature was then scanned from -80 °C to 100 °C at a temperature ramp rate of 3 °C/min. The glass transition temperature corresponds to the maximum in the tan delta, given by the ratio of the loss modulus to the storage modulus. The glass transition temperature was -9.1 °C.

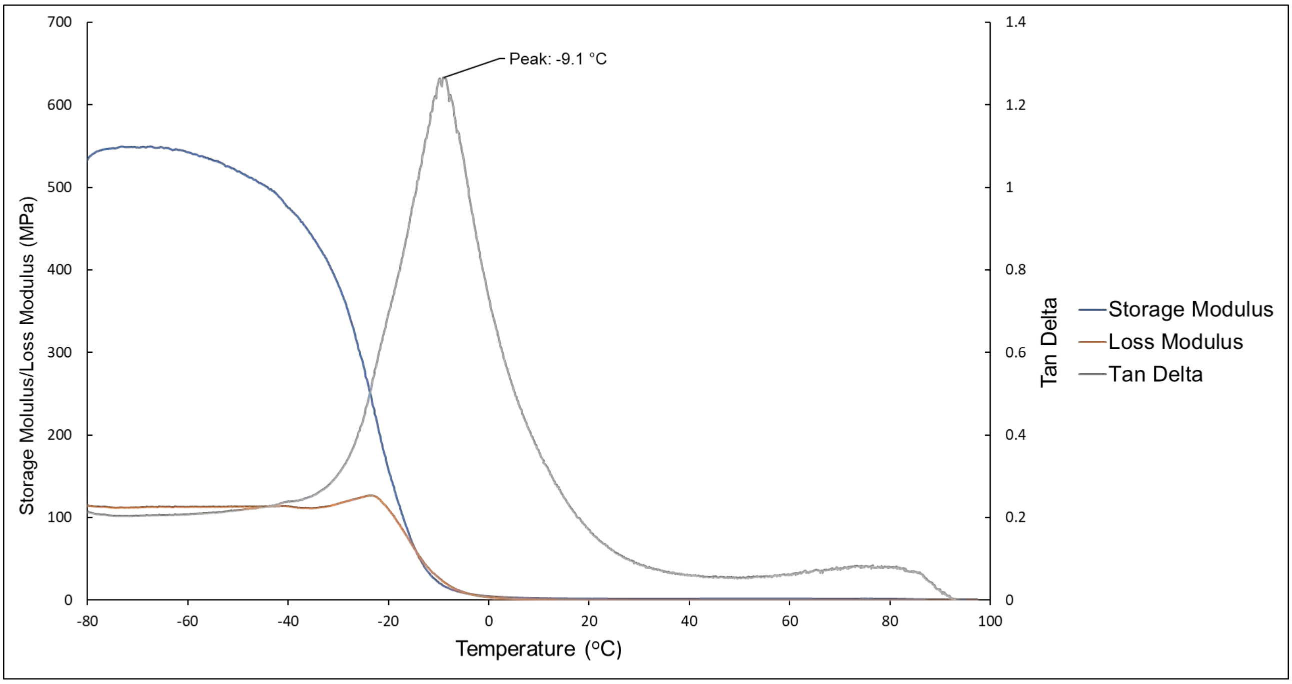

Tensile testing of polymer

The tensile strength of the polymers was tested using a dynamic mechanical analyser (TA Instruments Q800). The film tension clamp was used, with polymer pieces clamped at the wider clamping section. Care was taken to ensure that the clamp was not in contact with the gauge section as the strain caused by the clamp can lead to premature failure of the polymer. The DMA controlled force module was used. The force was ramped at a rate of 0.2 N/min with a maximum force of 18 N. However, the force never reached this point as the polymer would fail at lower force. A graph of the stress strain was produced for each of the samples. From this data, the tensile modulus was calculated using the slope of the stress-strain curve up to the yield point, which is determined by the point at where the stress strain curve loses linearity. The tensile modulus was 2.11 ± 0.09 MPa. The maximum tensile strength was determined by the stress at the yield point. The average tensile strength was 0.182 ± 0.001 MPa.

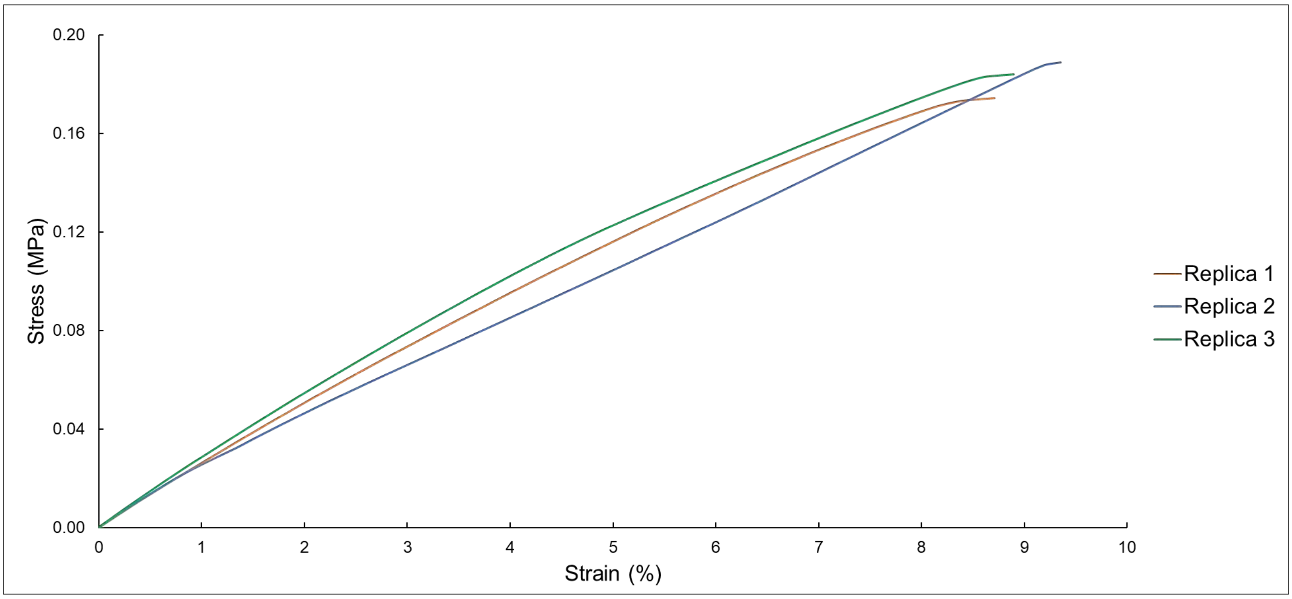

Compression testing

To measure the compression modulus of the polymer, a dynamic mechanical analyser (TA Instruments Q800) was used with a two-plate compression apparatus. The polymer was cut from the dog bone shaped pieces prepared above. Two samples were prepared with sides of 7.5 mm and a depth of 2 mm. The polymer was compressed with a force ramp rate of 3 N/min to a maximum of 18 N at room temperature. A stress-strain curve was then produced with the stress reported in MPa and the strain as a percent of original height. The compression modulus was calculated using the slope of this stress-strain curve, ignoring the initial section which is caused by sample irregularity and initial loading of the sample. The average compression modulus was 2.6 ± 0.5 MPa.

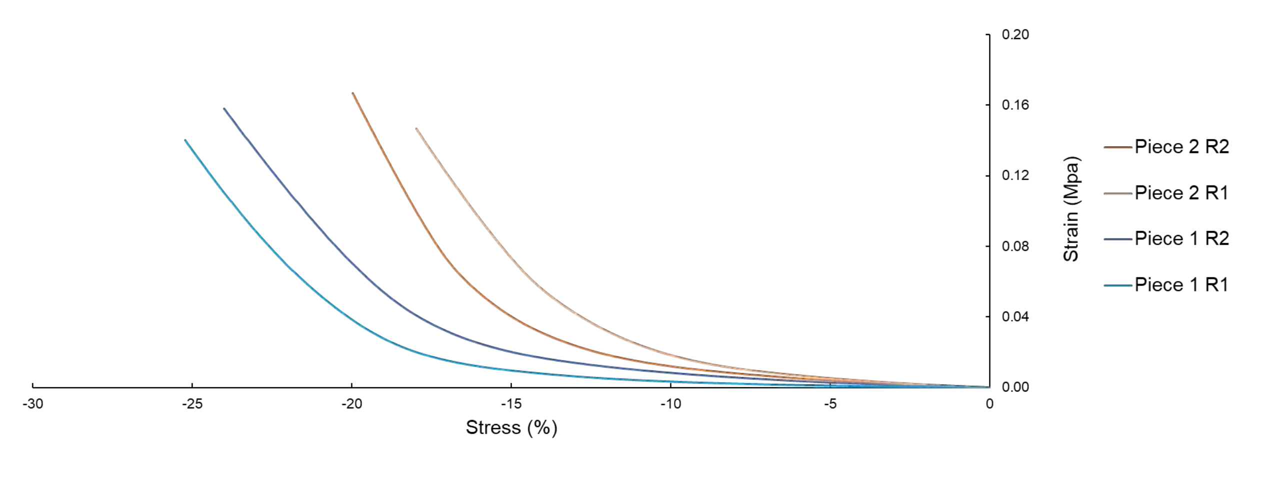

SEM characterization of the polymer

Scanning electron microscopy (SEM) images were obtained using an FEI F50 inspect system. EDX spectra were obtained using the EDAX Octane Pro detector. Samples were coated in a 20 nm layer of chromium using a Q300T-D Dual Target Sputter Coater to prevent charging of the polymer. All images were taken from the gage section of dog bone shaped polymers produced above. Both the surface and a cross-section of the polymer were imaged. The cross-section sample was obtained by cutting with a scalpel.

SEM images of the surface of the polymer:

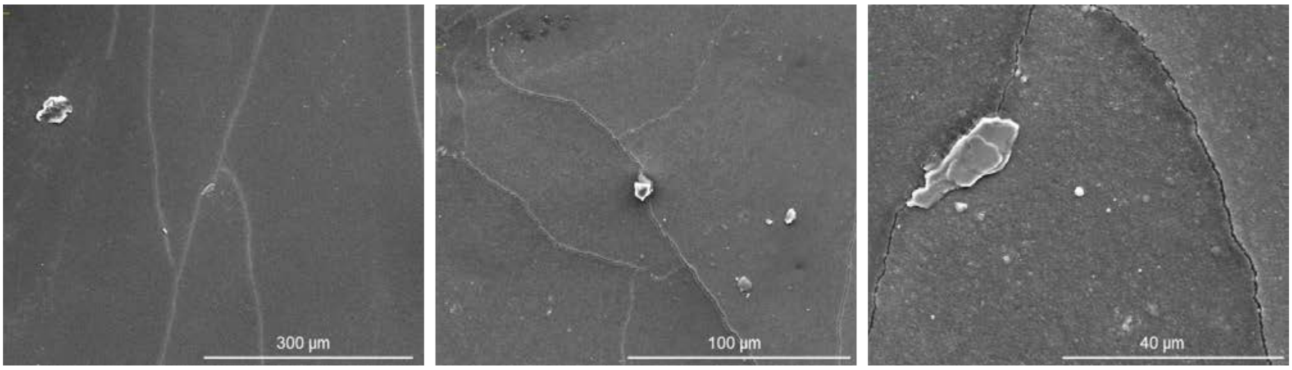

SEM images showing the cross section of the polymer:

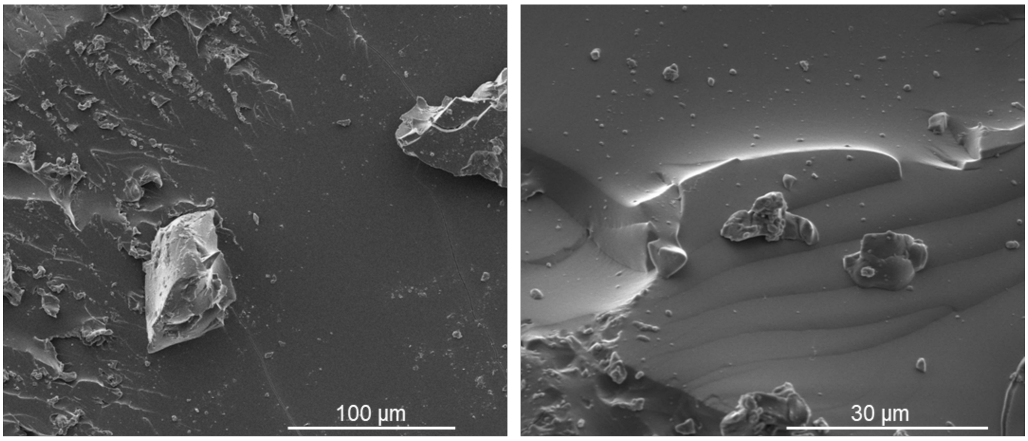

EDX characterisation of the polymer

EDX was used to assess the elemental composition of the polymer. The surface of the polymer showed uniform distribution of sulfur, carbon, and oxygen:

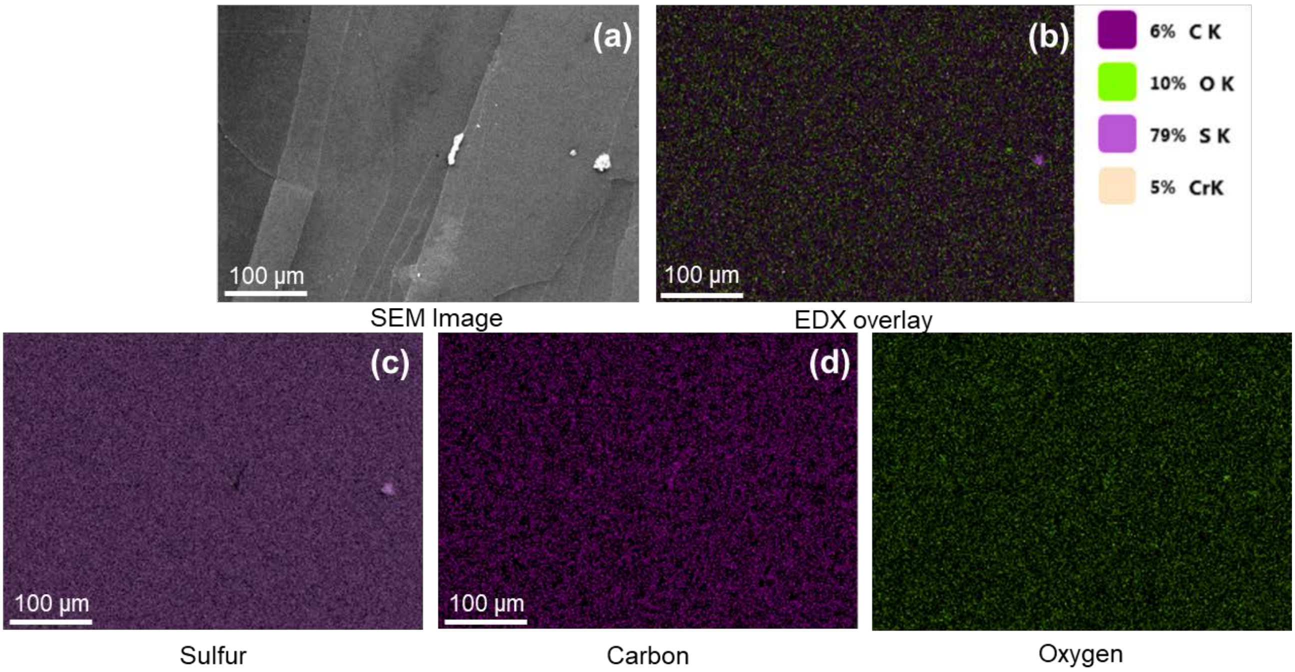

EDX images and analysis of the cross section of the polymer:

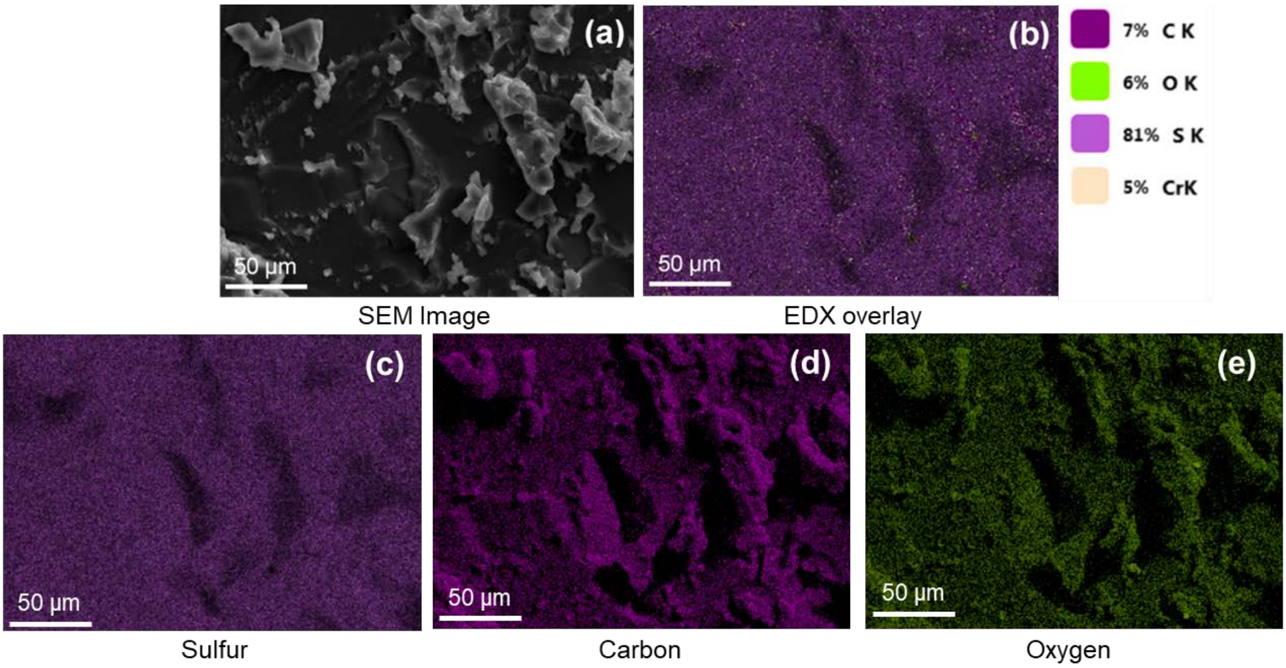

AFM analysis of the polymer surface

AFM images were acquired using a Bruker Multimode 9 AFM with a Nanoscope V controller using tapping mode in air, with all parameters including set-point, scan rate and feedback gains adjusted to optimize image quality. In order to minimize tapping force amplitude set-points, during scanning, were kept at 80 to 90% of the cantilever free amplitude. As a result no image or adhesion artefacts were observed in the AFM images indicating no tip contamination occurred due to the polymer surface.[2,3] The AFM probes used were Mikromasch HQ: NSC15 Si probes with a nominal spring constant of 40 N•m^{-1} and a nominal tip diameter of 16 nm. The scanner was calibrated in x, y and z directions using a silicon calibration grid (Bruker model number VGRP: 10 µm pitch, 180 nm depth). All analysis of AFM images was performed using Nanoscope analysis software version 1.4.

A small section of the polymer (approximately 5 × 5 mm) was cut using a scalpel and mounted on a stainless-steel disc (10 mm diameter) using double sided tape. The top surface of the polymer that was exposed to air when curing was analysed using AFM. Ten 10 × 10 µm images were acquired on the polymer sample at distinctly separate locations (i.e. the tip was disengaged from the surface and moved some hundreds of microns in the X and Y directions before re-engaging). The AFM topography image shown below has been flattened and the displayed cross section was generated using the section tool of the AFM analysis software and corresponds to the position of the white line in the AFM image. Roughness analysis was performed on each AFM image and an average taken with the error in the roughness measurements reported representing one standard deviation in the data. The average roughness was Ra = 8.84 ± 1.28 nm and the root mean square roughness was Rq = 11.59 ± 1.71 nm.

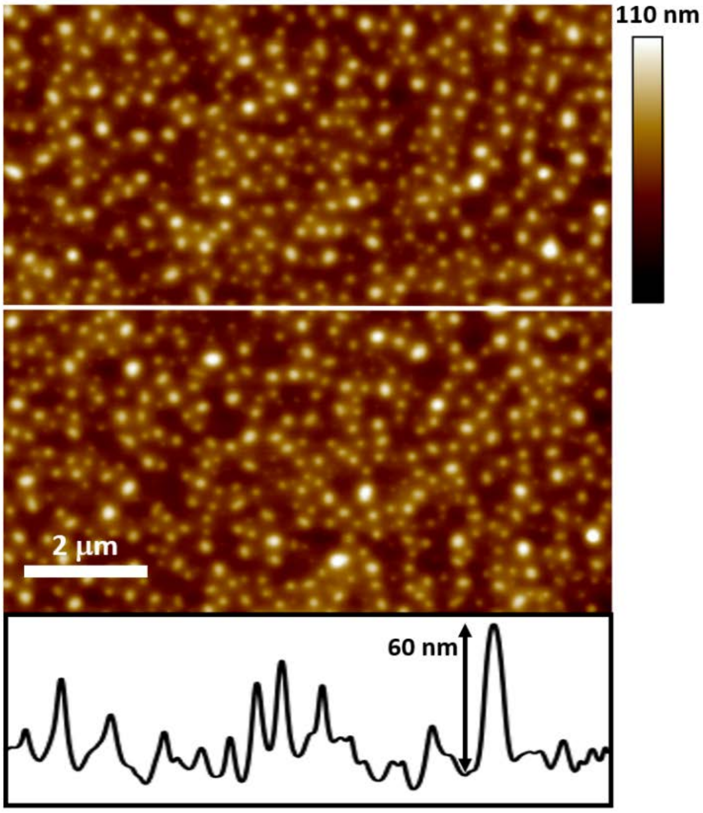

Thermal analysis of the polymer (TGA and DSC)

The DSC heat flow and the TGA mass loss of the polymer were determined using a Perkin Elmer STA8000 simultaneous thermal analyser. 14.4 mg of the polymer was ground into a powder and added to the STA. The temperature was scanned from 50 °C to 600 °C at a rate of 20 °C/ min under nitrogen. After the temperature reached 600 °C, oxygen was added to burn off any remaining products. The characteristic phase changes of sulfur between 100 and 130 °C (transition from orthorhombic to monoclinic crystalline state and then melting) was not observed, indicating that the sulfur monomer was completely consumed in the reaction.

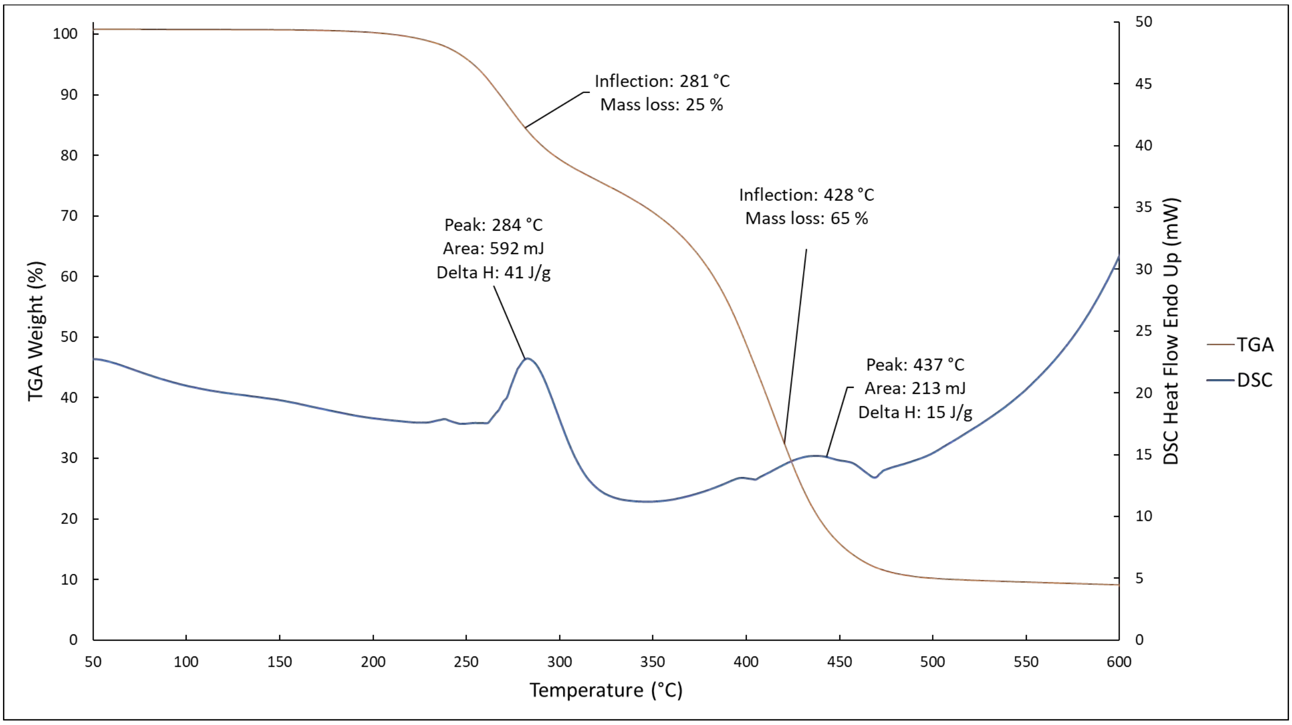

Raman spectroscopy characterization of polymer

Raman spectra were collected using a Witec alpha300R Raman microscope at an excitation laser wavelength of 532 nm with a 40X objective (numerical aperture 0.60). Typical integration times for Raman spectra were 5 to 10 s. Raman data was acquired on a cross section of a polymer sample that was cut using a clean scalpel. Approximately 100 Raman spectra were acquired at five distinct locations with each region separated by hundreds to thousands of microns. None of the Raman spectra collected on the cross section of the polymer corresponded to that expected for pure elemental sulfur.

Raman analysis was also performed on the top surface of a polymer sample at five distinct locations with each region separated by hundreds to thousands of microns. In this instance the Raman spectra for pure elemental sulfur was observed at certain locations. We attribute this sulfur to that formed by sublimation during the curing process.

Raman spectrum for pure elemental sulfur for reference:

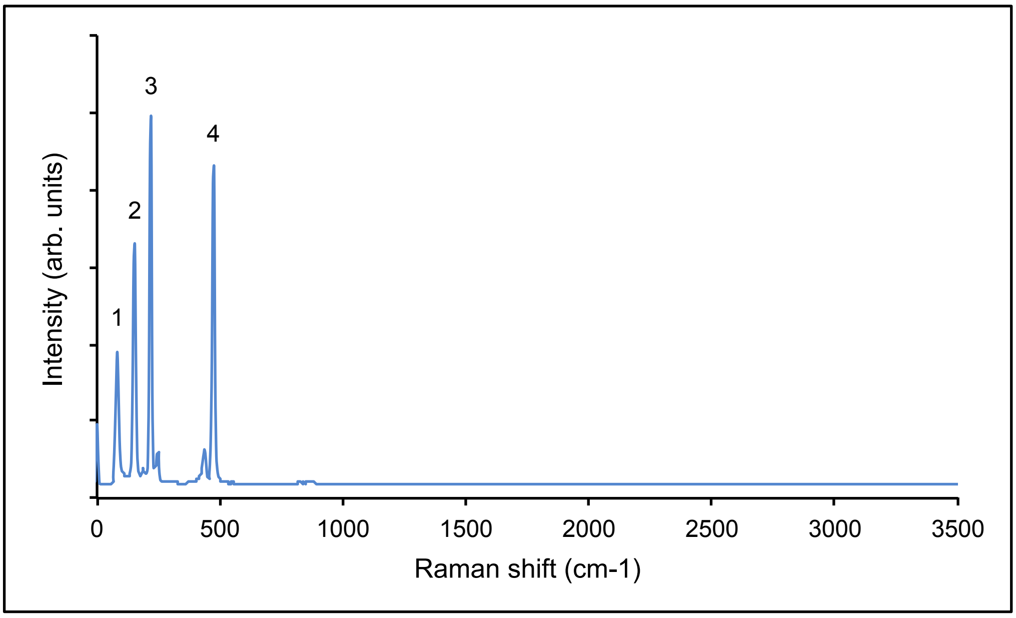

Peak number	***Raman shift (cm^{-1})***
1	82
2	154
3	220
4	474

Raman spectrum for polymer cross section:

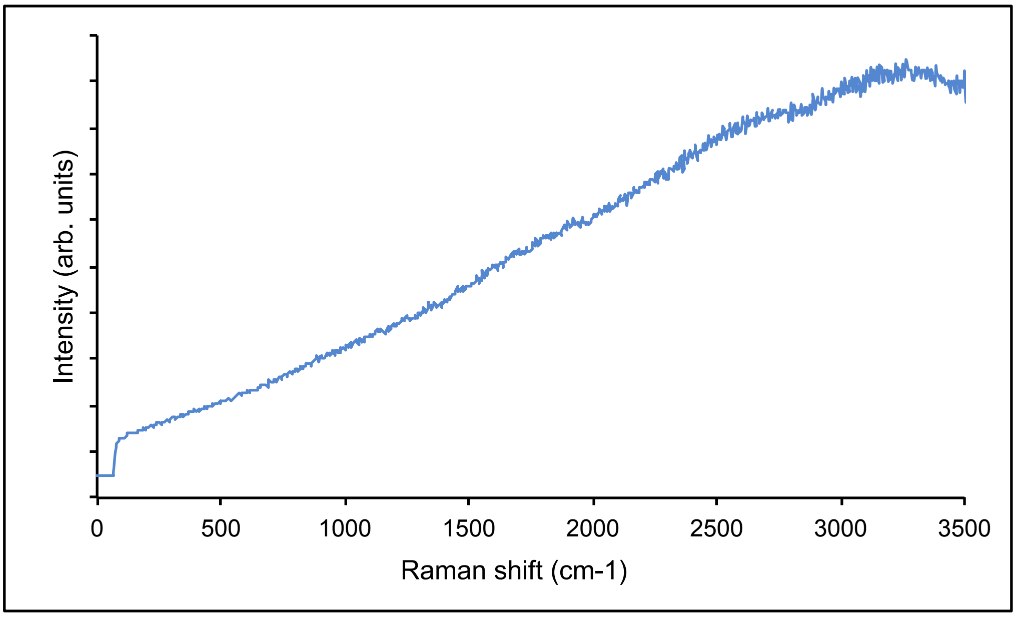

Raman spectrum for polymer surface:

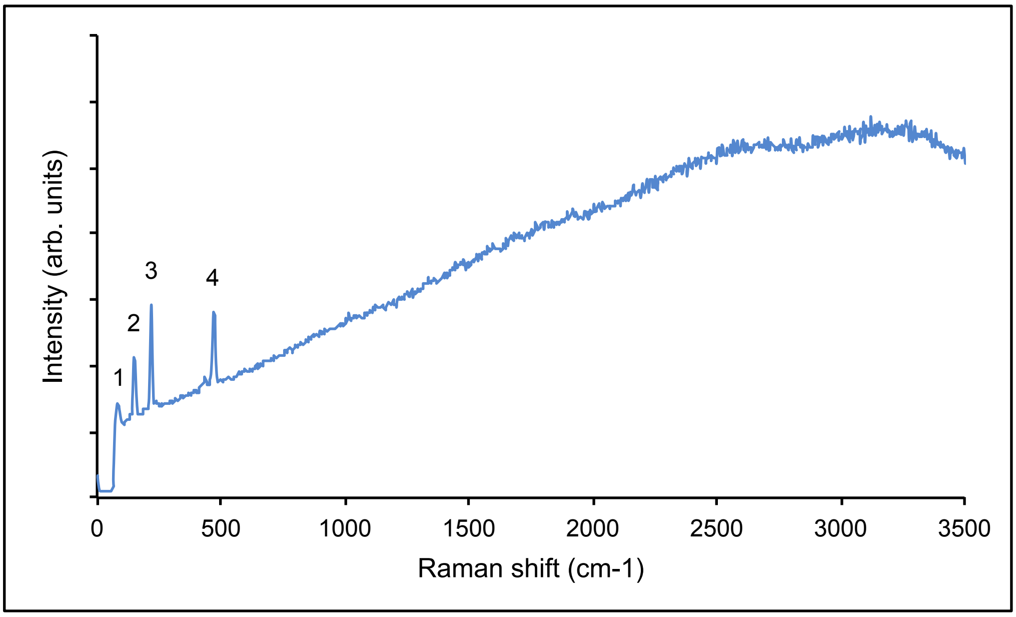

The strength and Raman shift for each of the numbered peaks is shown below.

Peak number	***Peak Raman shift (cm^{-1})***
1	86
2	151
3	219
4	472

IR characterization of polymer

An infrared spectrum of the polymer was produced with a Perkin Elmer Spectrum 100 Fourier Transform spectrophotometer using the ATR method. The energy of the electromagnetic radiation is reported in wavenumbers and the intensity is given as percent transmittance. The polymer was cut and ground before being placed directly on the ATR crystal. Characteristic signals appear at 2923 cm^{-1} (C-H groups in the DCPD and canola oil) and 1742 cm^{-1}(C=O group in the canola oil trigyceride).

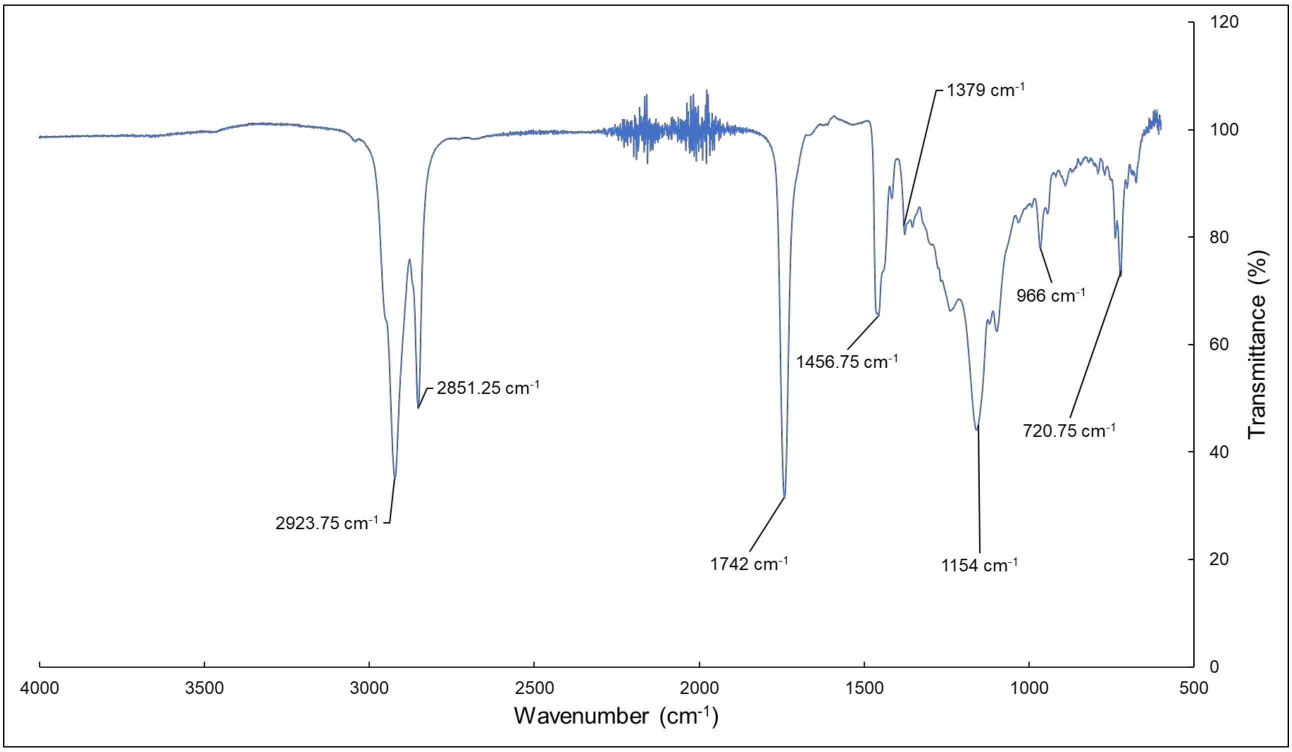

NMR characterization of the polymer

^{1}H NMR spectra were acquired using a 600 MHz spectrometer using deuterated pyridine-d5 as a solvent. A small amount of polymer was cut from a dogbone sample for this analysis. The polymer was placed directly in an NMR tube with deuterated pyridine and sonicated for 30 minutes to break down and dissolve the polymer. While the ^{1}H NMR spectrum acquired is for the product of the reaction of the polymer with pyridine, rather than the polymer itself, the spectra is still useful in calculating the alkene conversion. This was done by assessing the change in the ratio of alkene protons to the methine signal of the triglyceride before and after the polymerization. NMR spectra of both canola oil and DCPD in pydidine-d5 are also provided for reference, as well as an NMR spectrum containing unreacted DCPD and canola oil at the same molar ratio as used in the polymerization.

^{1}H NMR spectrum of canola oil (600 MHz, pyridine-d5):

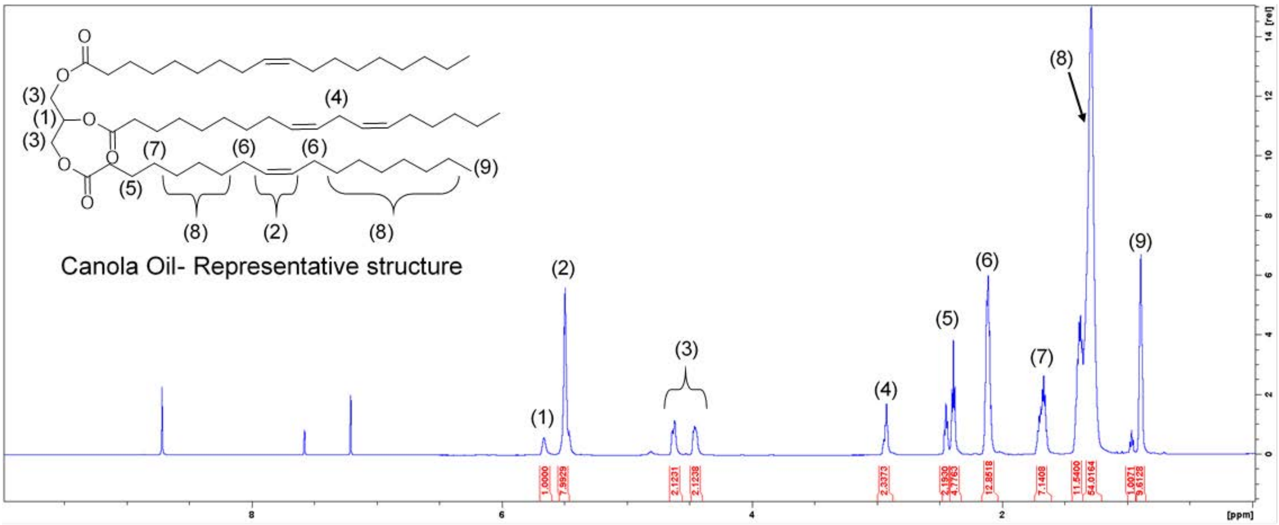

^{1}H NMR spectrum of DCPD (600 MHz, pyridine-d5):

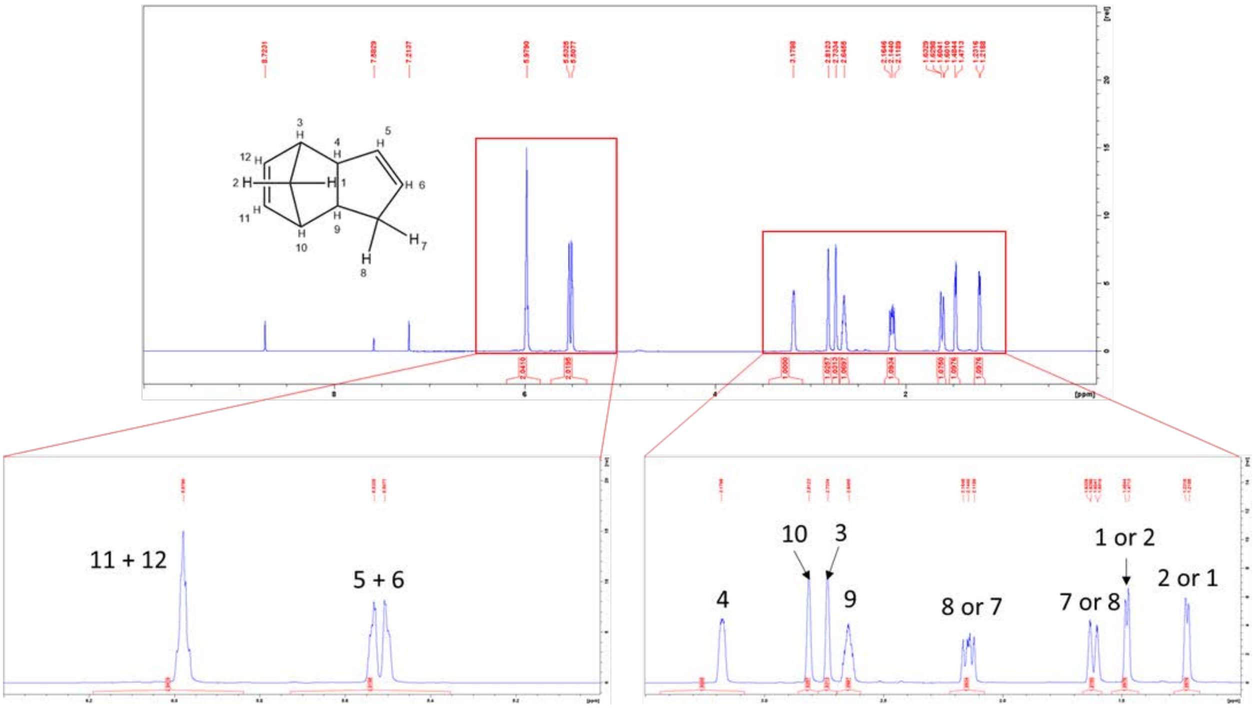

^{1}H NMR spectrum (600 MHz, pyridine-d5) of unreacted canola oil and DCPD, prepared in the same ratio as used in the polymerization (mass ratio of 35:15 canola oil:DCPD). This corresponds to a molar ratio of canola oil to DCPD of 1 : 2.85. The portion of the spectrum between 5 ppm and 6.5 ppm was zoomed in to show alkene peaks along with the methine (CH) group in the glycerol group of the canola oil triglyceride. This CH group does not react in the polymerization, so it can be used as an internal standard. The ratio of the alkene peaks to the CH group of the glycerol can then be used to calculate alkene conversion (see next page).

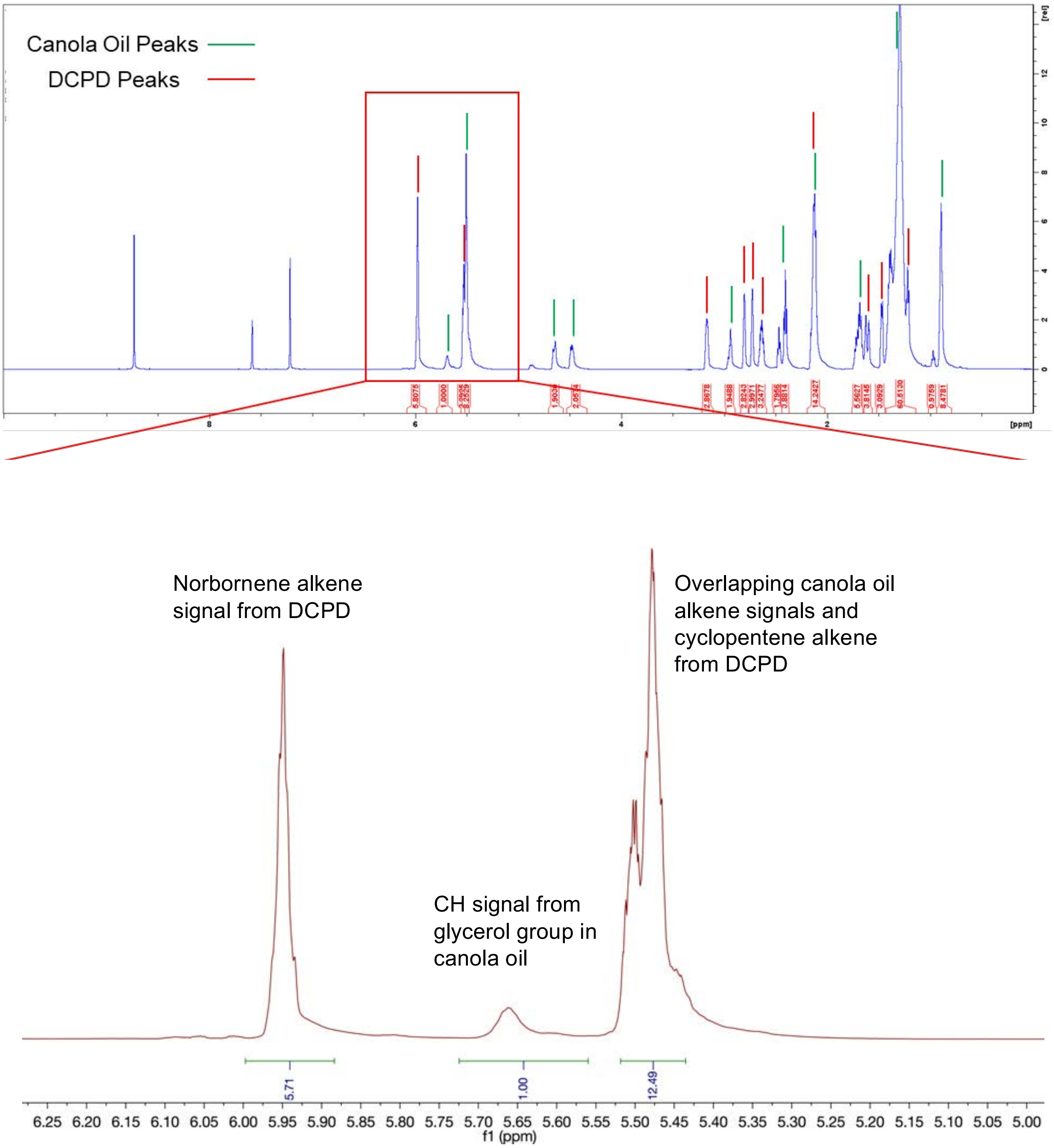

^{1}H NMR spectrum of terpolymer made from sulfur (50 wt%), canola oil (35 wt%) and DCPD (15 wt%). (600 MHz, pyridine-d5). The norbornene signal from DCPD was completely consumed. The ratio of unreacted alkenes to the CH group from the glycerol in canola oil was 0.71 : 1.00. Before the polymerization, this ratio was 12.49 : 1.00 (see previous page). This means that a combined 94% of the alkenes in the canola oil and the cyclopentene group of DCPD reacted in the polymerization.

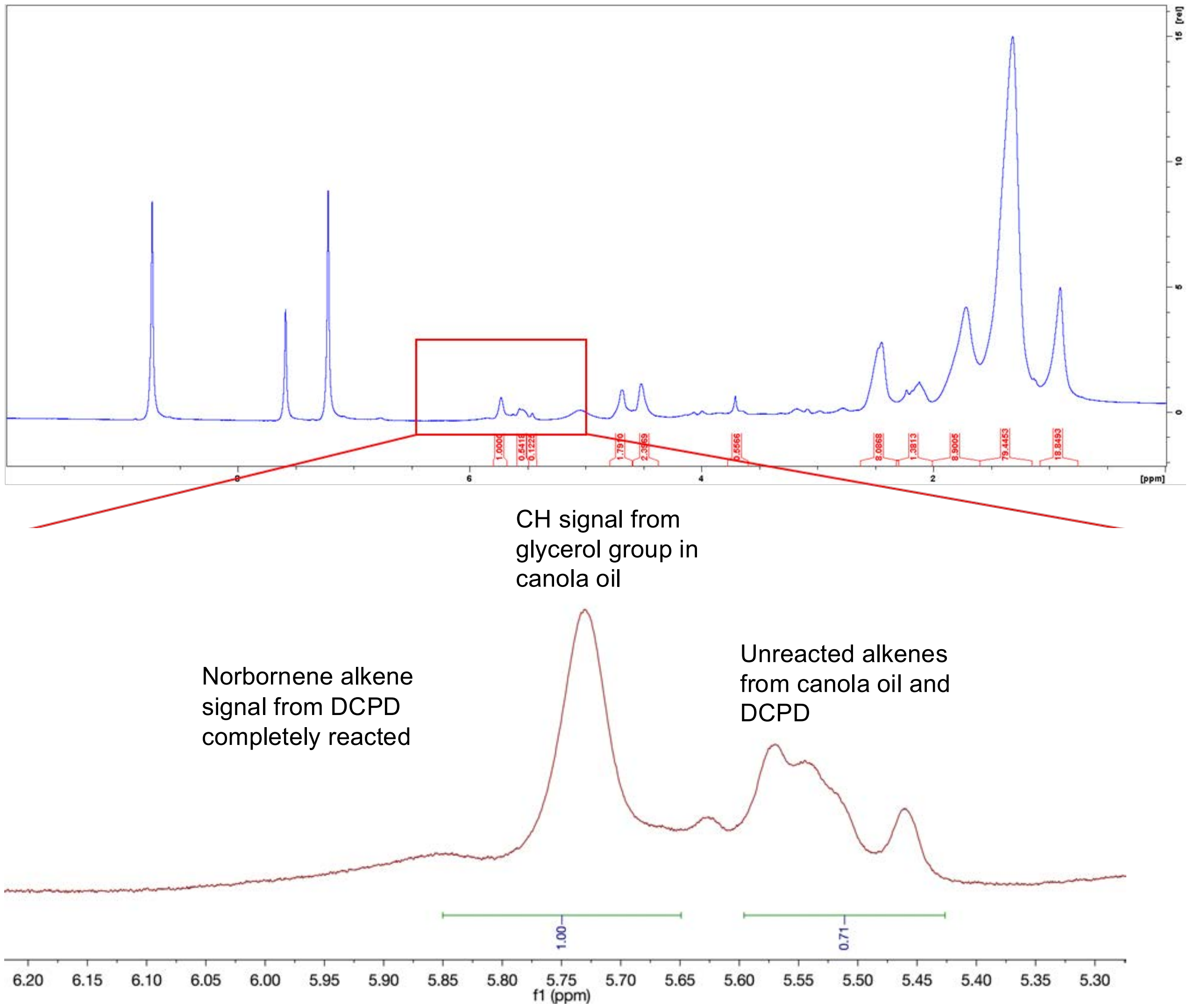

Repair of dogbone sample using pyridine and tributylphosphine

The dogbone polymer pieces were cut the entire way through the centre of the gage section using a scalpel to produce two halves. To one of the polymer pieces either pyridine and tributylphosphine was added by pipette to the cut surface. The two polymer pieces were then returned to the mold so that the cut interfaces were in passive contact (no pressure was applied to force the pieces together). The volumes of inducer tested were 1 µL, 5 µL, 10 µL and 15 µL of both pyridine and tributylphosphine. Each volume was tested in triplicate. These volumes correspond to 12.4 µmol, 62.1 µmol, 124 µmol and 186 µmol for pyridine and 4.05 µmol, 20.3 µmol, 40.5 µmol and 60.8 µmol for tributylposphine. After 24 hours, the pieces were removed from the mold and all pieces showed adhesion. The full process is shown in the images below:

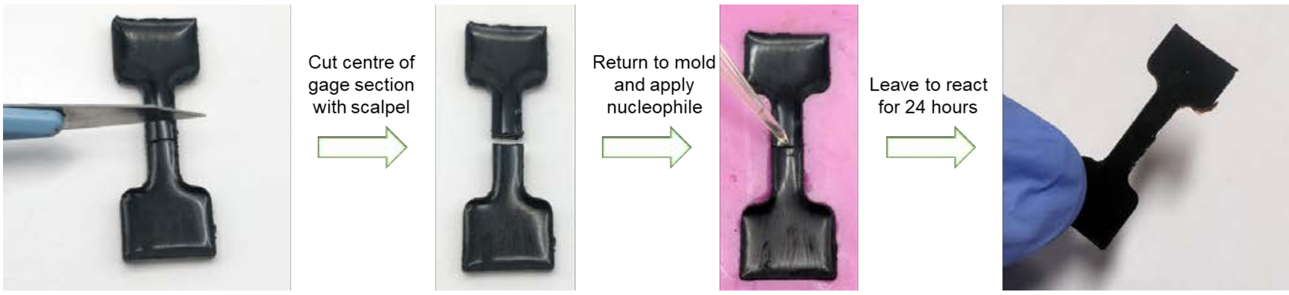

The strength of adhesion at the repaired interface was tested using dynamic mechanical analysis. The film tension DMA clamp was used with the DMA controlled force module. Care was taken to ensure that the clamp was not in contact with the gage section as the strain caused by the clamp can lead to premature failure of the polymer. The force was ramped at a rate of 0.2 N/min with a maximum force of 18 N. No temperature control was used and a stress strain curve was produced for each of the samples. From this, the strain at failure could be determined. The pieces failed at the repaired interface. Therefore, the strain at failure indicates the strength of adhesion caused by the tributylphosphine or pyridine induced reaction. Three replicas of each of the volumes of pyridine or tributylphosphine were tested as well as three replicas of undamaged polymer pieces as controls. A control of the cut polymer with no catalyst was also tested but showed no adhesion and could therefore not be tested using DMA. The results on tabulated below and on the following page.

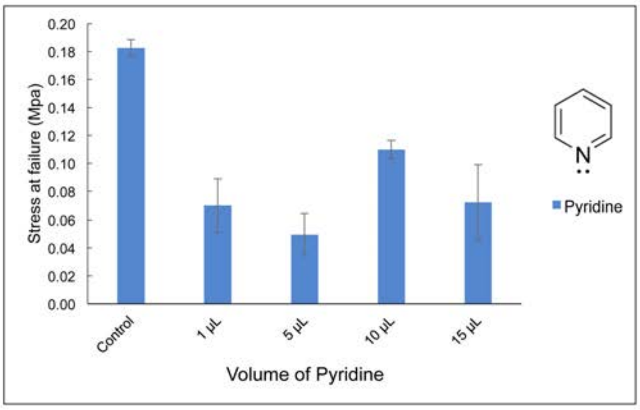

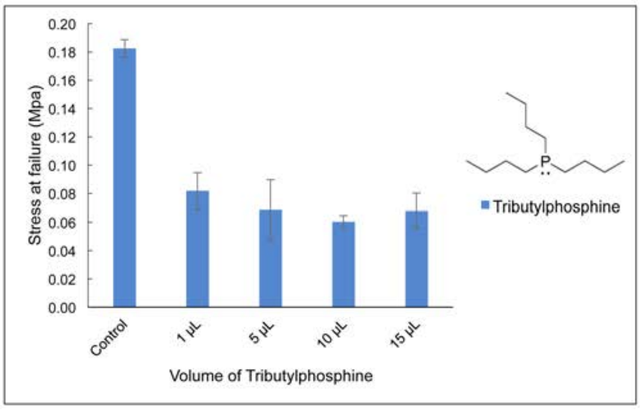

Average of three replicas					
	Volume	Max strain (MPa)	Standard deviation (MPa)	Strain at yield of repaired sample / strain at yield for control *100%	Tensile stress at yield (%)
Control	N/A	0.182	0.006	N/A	9.0
Pyridine	1 µL	0.07	0.02	38	4.7
	5 µL	0.05	0.02	27	4.6
	10 µL	0.110	0.006	60	6.3
	15 µL	0.07	0.03	38	4.5
Tributylphosphine	1 µL	0.08	0.01	44	3.8
	5 µL	0.07	0.02	38	5.1
	10 µL	0.060	0.004	33	4.5
	15 µL	0.07	0.01	38	4.9

Time course of polymer repair

To gain an understanding into the time required to repair the polymer with pyridine and tributylphosphine, the adhesion strength was determined for several different reaction times. Dogbone shaped polymer pieces were cut through the centre of the gage section and repaired as described above using the volume of pyridine and tributylphosphine that corresponded to the greatest adhesion strength. This volume was 10 µL of pyridine and 1 µL of tributylphosphine, as determined in the previous experiment. Three replicas were prepared and left to react without pressure for time periods of 1, 2, 4, 6, 12 and 24 hours for both pyridine and tributylphosphine. After the reaction had been left for the appropriate amount of time, the repaired polymer pieces were tested for tensile strength using dynamic mechanical analysis with the same method as described earlier.

The samples that were repaired with pyridine reached a maximum adhesion strength after two hours which then remained constant up to 24 hours. The one hour sample had approximately half the adhesion strength of the 24 hour sample, indicating that it takes between one and two hours for the pyridine induced reaction to reach a maximum adhesion strength. The samples that were repaired with tributylphosphine reached a maximum adhesion strength after one hour. The adhesion strength then remained relatively constant for all other time periods, up to 24 hours. This indicates that the reaction with tributylphosphine is faster than that of pyridine, reaching maximum adhesion strength after one hour. Note that all but the one hour sample of pyridine showed a stronger adhesion than tributylphosphine.

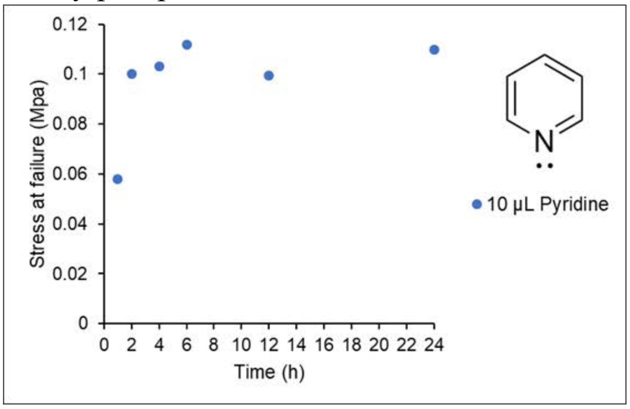

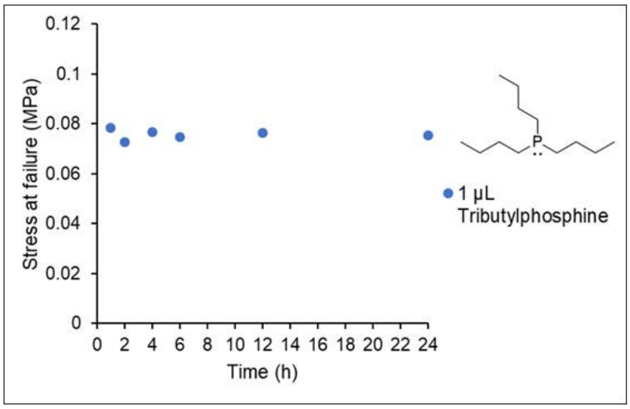

Optimization of adhesion strength using pressure

For pyridine or tributylphosphine to induce polymer repair, the polymer interfaces must be directly in contact. By applying pressure between the interfaces, contact between the polymer interfaces throughout the reaction could be ensured. The polymer pieces were compressed with a 3D printed apparatus which could apply controlled and consistent compression to twelve dogbone shaped polymer pieces. The compression apparatus was designed on Autodesk fusion 360. The shape of the dog bone polymer as described above was used to make a six by two grid of indentations with a height that corresponded to the desired final compressed height of the polymer pieces. The indentations were placed 4 mm away from each other, both vertically and horizontally. The base was extended an additional 5 mm from the sides of the outer indentations and extruded 4mm below the indentations. Another flat piece was also 3D printed with a size of 102 mm by 84 mm with a depth of 4 mm, matching the other piece but without the indentations. The polymer could then be added to the indentations and the second piece could be clamped on top. The pieces were clamped together using 4, 50 mm C clamps. This caused the pieces to be compressed to the width of the indentation while maintaining their shape. Several indentation thicknesses were tested. Indentation thicknesses of 10 %, 20 %, 30 %, 40 % and 50 % were produced but damage to the polymer became evident with compression greater than 30 %. A compression of 10 % corresponds to a reduction of 10 % of the original thickness of the polymer. The indentations for the 10 %, 20 %, 30 %, 40 % and 50% apparatus corresponded to depths of 1.8 mm, 1.6 mm, 1.4 mm, 1.2 mm and 1 mm respectively. The 3D printed apparatus can be seen below:

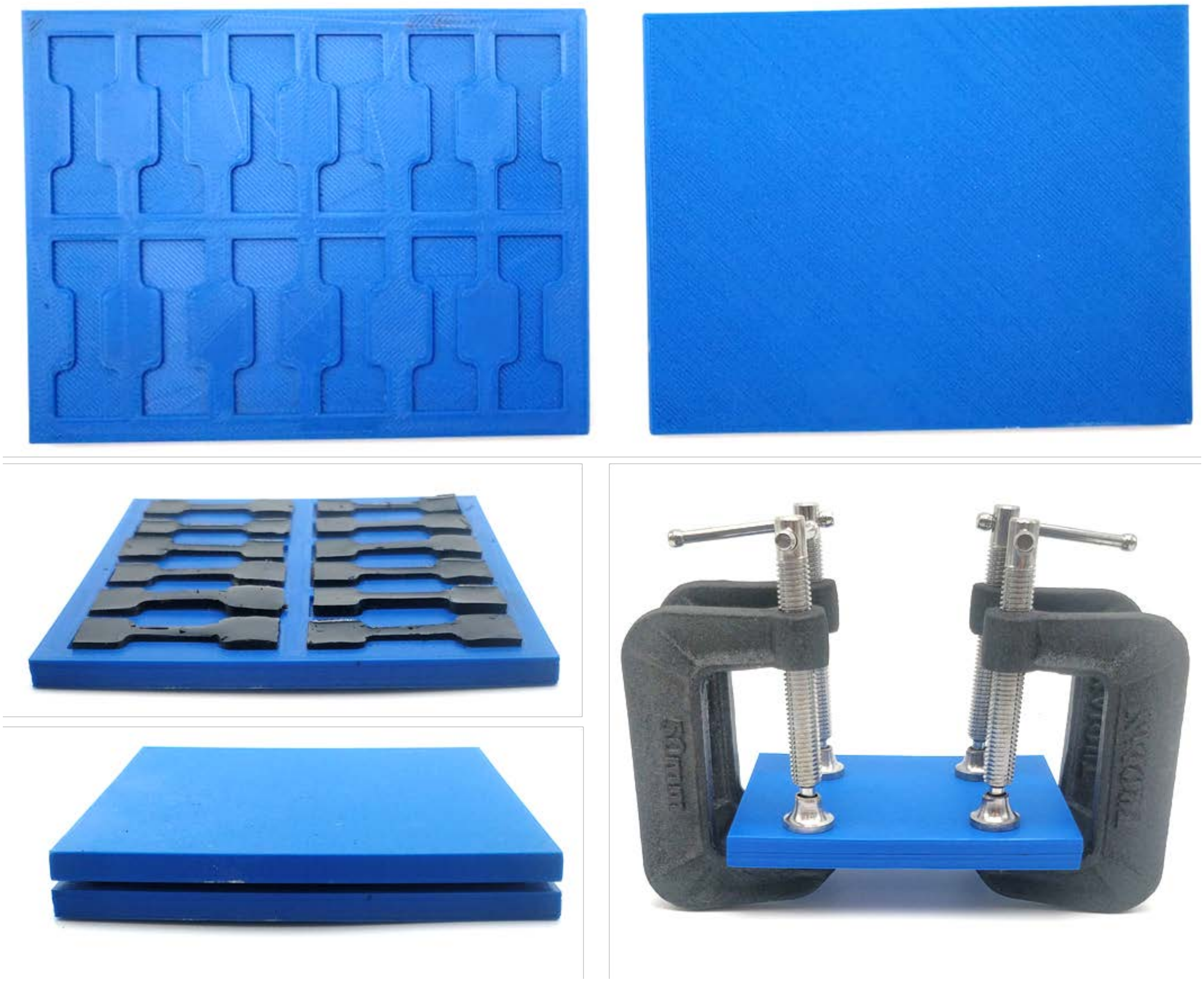

The volume of pyridine and tributylphosphine which corresponded to the greatest adhesion strength were selected for use in the compression tests. For pyridine, it was 10 µL and for tributlyphosphine, it was 1 µL. The polymer pieces were cut with a scalpel in the centre of the gage section as with earlier tests. They were then placed in the compression mold and the pyridine and tributylphosphine was applied to the cut interface using a micropipette. Several controls were also included which were compressed but were not cut and others that were cut but not had no pyridine or tributylphosphine. The top 3D printed piece was then placed on top of the polymers and secured in place using four 50 mm C clamps. The C clamps were tightened until the top piece was flat against the piece with the indentations. The polymer was then left for 24 hours under compression. After 24 hours, the repaired pieces were removed from the indentations and tested for tensile strength using a dynamic mechanical analyzer with the same method as earlier tests. All controls which had no pyridine or tributylphosphine applied showed no adhesion, while the undamaged control samples showed no decrease in tensile strength for compression of less than 30 %. For compression over 30 %, significant damage occurred, and the pieces were not appropriate for mechanical testing. The strain at failure was used as a measure of the adhesion strength of the repaired pieces. The results are compiled below. The repair for pyridine was increased to 73% of the strain observed in the control dogbone pieces. For tributylphosphine, not as much improvement was observed. It appears that for pyridine, which is less reactive than tributylphosphine, requires more time and compression to ensure efficient contact and reaction time at the interface of the damaged polymer.

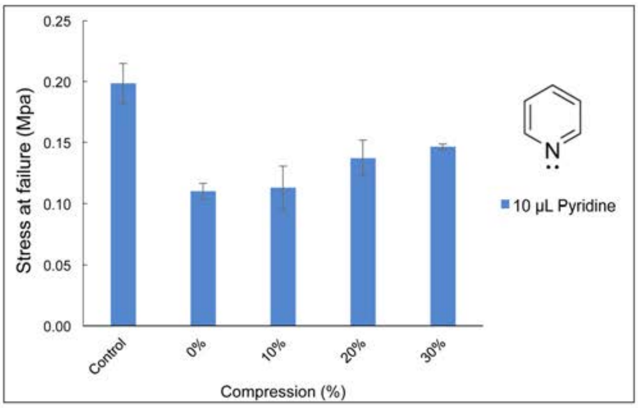

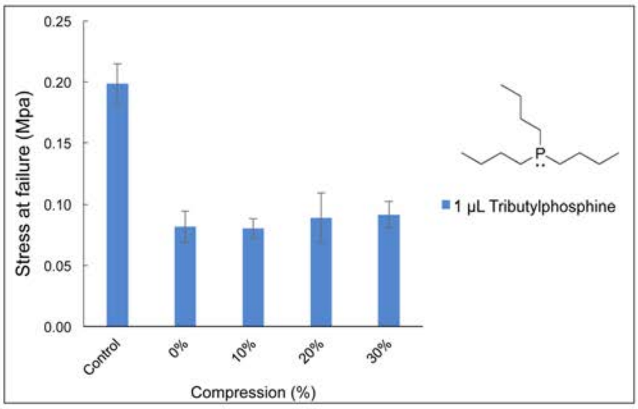

Average of three replicas					
	Compression	Stress at Failure (MPa)	Standard deviation (Mpa)	Strain at yield of repaired sample / strain at yield for control *100%	Tensile strain at Yield (%)
Control	Control	0.20	0.02	N/A	10.3
Pyridine	0%	0.110	0.006	55	6.3
	10%	0.11	0.02	55	4.8
	20%	0.14	0.01	70	5.5
	30%	0.146	0.002	73	6.1
TBP	0%	0.08	0.01	40	3.8
	10%	0.080	0.008	40	4.2
	20%	0.09	0.02	45	4.4
	30%	0.09	0.01	45	4.2

Polymer solubility and adhesion with conventional solvents

To differentiate the adhesion induced by pyridine and tributylphosphine catalyzed S-S metathesis with simple polymer welding, solubility tests and the repair using conventional solvents was tested. This experiment was done to rule out polymer adhesion as a result of dissolution and entanglement of the polymers at the interface. For linear polymers with no crosslinking, solvents like acetone or chloroform can often dissolve the polymer. However, highly crosslinked polymers will not readily dissolve without first breaking the crosslinking chains. To test the solubility of the polymer, 100 mg of the polymer was cut into pieces and placed in 1 ml of solvent. The polymer was left for 4 hours before being filtered and washed with acetone. The polymer was then left for an additional two hours to allow the acetone to evaporate before being weighed to find any change in mass due to dissolved polymer. The solvents that were tested were acetone, chloroform, tetrahydrofuran (THF), dimethylformamide (DMF), toluene and ethanol. These were compared with pyridine and tributylphosphine (TBP in the images below) which were suspected to break the crosslinking S-S bonds in the polymer. The only solvents that showed a significant change in mass were pyridine and tributylphosphine. This indicates that a significant amount of the polymer has been dissolved in these solvents. It was also evident that pyridine and tributylphosphine dissolved the polymer as they became a dark brown colour over time. This colour change was not observed in any of the other solvents, indicating minimal solubility of the polymer. As the polymer only showed significant solubility in pyridine and tributylphosphine and not the other solvents, it can be deduced that the polymer is not simply dissolving, rather, the crosslinking S-S bonds are being broken down in the pyridine and the tributylphosphine, providing a lower molecular weight product that is soluble. Below are images of the polymer in a range of solvents showing the dark colour of the pyridine and tributylphosphine samples. Tabulated solubility data is provide on the next page.

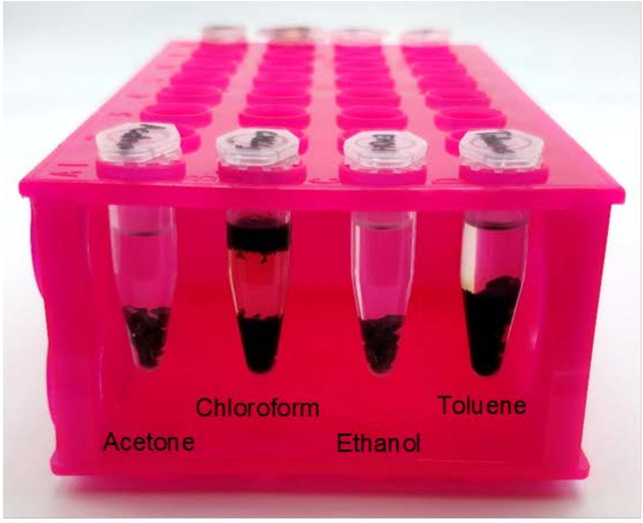

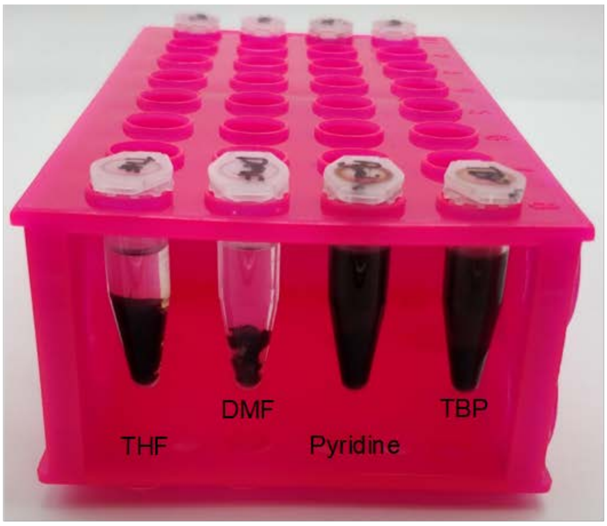

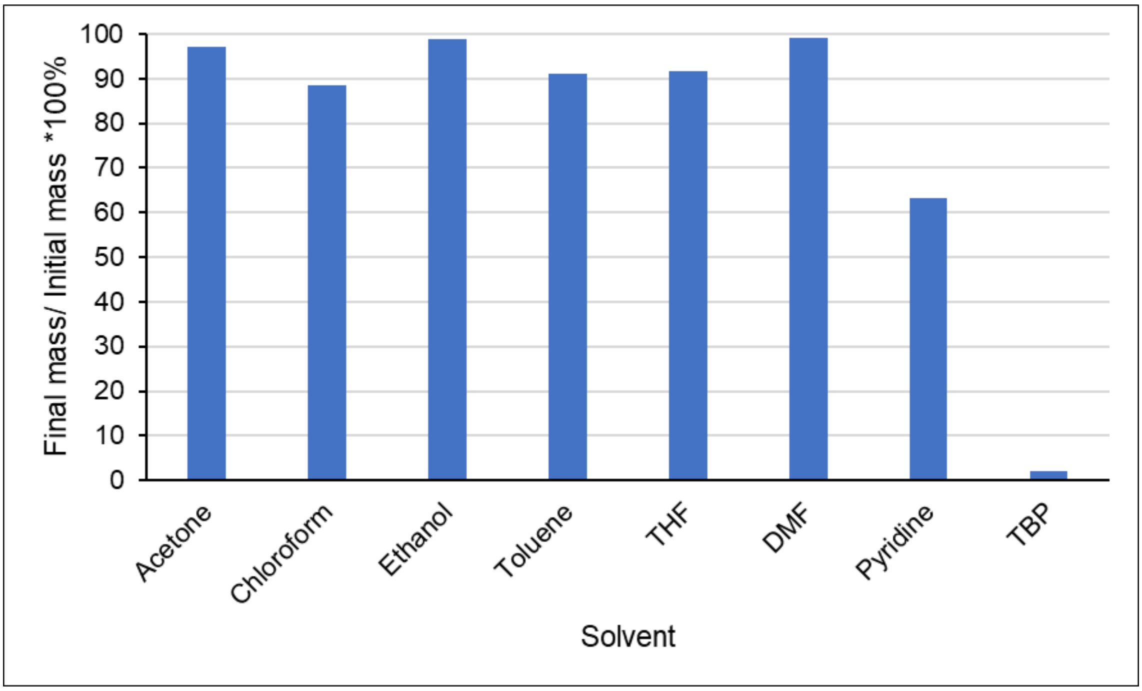

Solvent	*Initial mass of polymer (mg)*	*Final mass of polymer (mg)*	*Change in mass of polymer (mg)*	*Percent of polymer remaining (%)*
Acetone	100.2	97.2	3.0	97
Chloroform	100.7	88.6	12.1	88
Ethanol	100.1	99.1	1.0	99
Toluene	101.4	92.3	9.1	91
THF	99.6	91.6	8.0	92
DMF	99.9	98.9	1.0	99
Pyridine	100.5	63.3	37.2	63
TBP	101.2	2.0	99.2	2

To further show that the adhesion is not due to polymer welding, the adhesion of the polymer was tested using the conventional solvents outlined above. Six dogbone shaped polymer pieces were cut down the middle with a scalpel in the same way as the earlier adhesion tests. The polymer pieces were then placed in the 10 % compression apparatus. Using the same method as previous adhesion tests with pressure, 10 μL of the corresponding solvent was applied with a micropipette to the cut interface of the polymer piece. This was done for all six solvents. The top piece of the compression apparatus was placed on top and clamped with four, 50 mm C clamps. Compression was used to ensure good contact between the polymer interfaces. The polymer pieces were then left for 24 hours like the pyridine and tributylphosphine samples in earlier tests. After 24 hours, the clamps and top piece of the compression apparatus were removed. All polymer pieces showed no evidence of adhesion. When lifted from one side, the polymer pieces separated with no resistance. Photos of the polymer pieces after solvents were applied and left for 24 hours are provided on the next page.

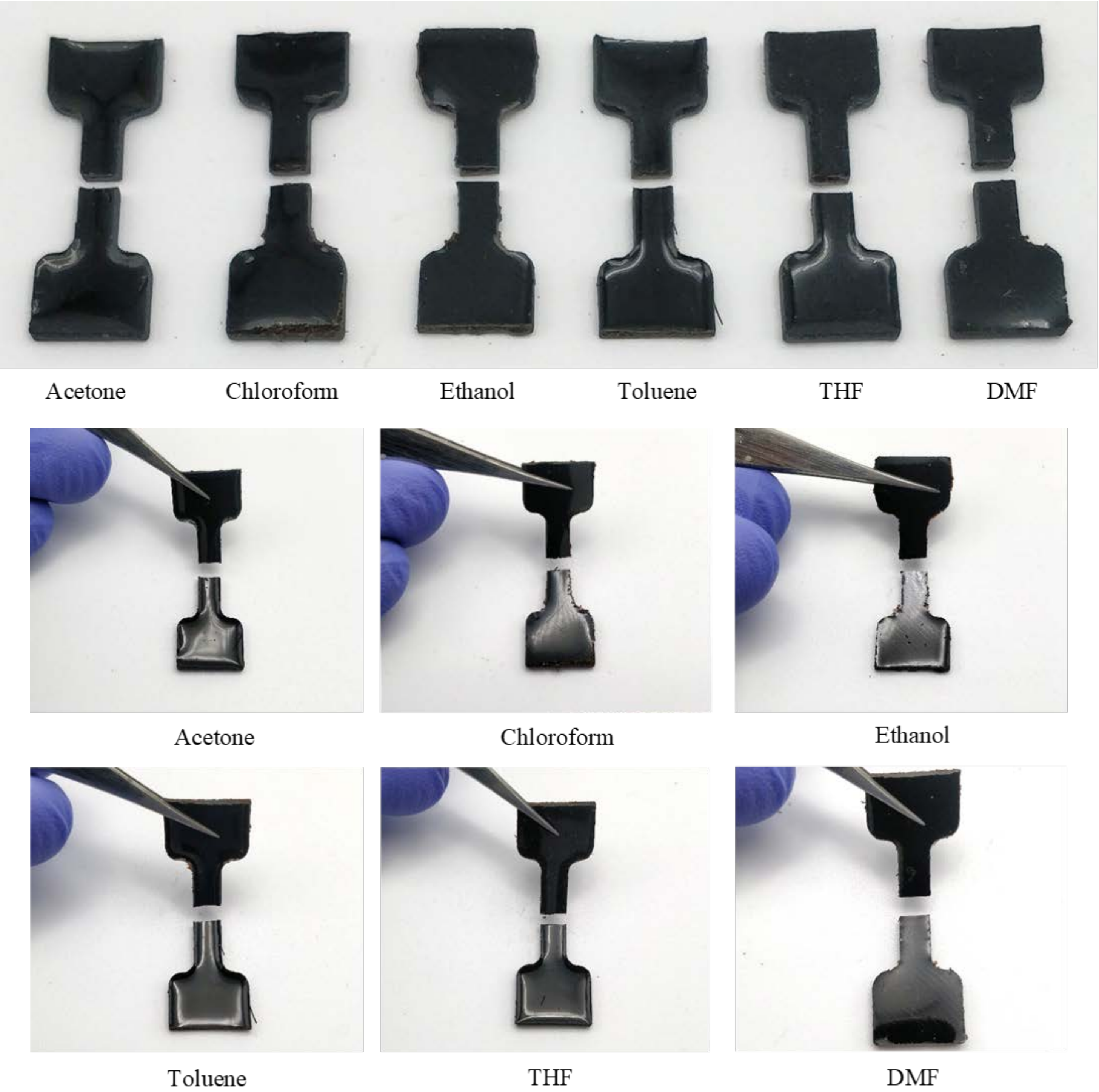
Acetone
Chloroform
Ethanol
Toluene
THF
DMF
Acetone
Chloroform
Ethanol
Toluene
THF
DMF

SEM and EDX of repaired interfaces

Dogbone shaped polymer pieces were cut in the centre of the gage section and repaired using 1 µL of tributyphosphine and 10 µL of pyridine according to the procedure used in adhesion strength testing. This was performed for the range of compressions tested in the adhesion strength testing. The gage section was then cut with a scalpel along the length of the dogbone so that a cross section of the repaired surface could be imaged. The cut polymer cross sections were coated in 20 nm of chromium using a Q300T Dual Target Sputter Coater and analyzed using SEM and EDX to investigate the adhesion and identify the presence of the inducers used. The adhesion was clearly visible in the SEM images, which showed areas where the polymer had reacted at the interface to form a continuous bulk polymer material. This reaction would only occur where the interface was in contact and a gap was still visible where the interfaces were not in contact. The proportion of the reacted surface to the unreacted surface at the interface increased significantly when the polymer pieces were compressed throughout the repair. This can be seen in the images below for two samples that were repaired with pyridine, one with no compression and one with 10% compression. The increase in reacted surface area from the uncompressed samples was obvious and significant when 10% compression was applied, however, only minimal improvements were observed for increases in compression from 10% to 30%.

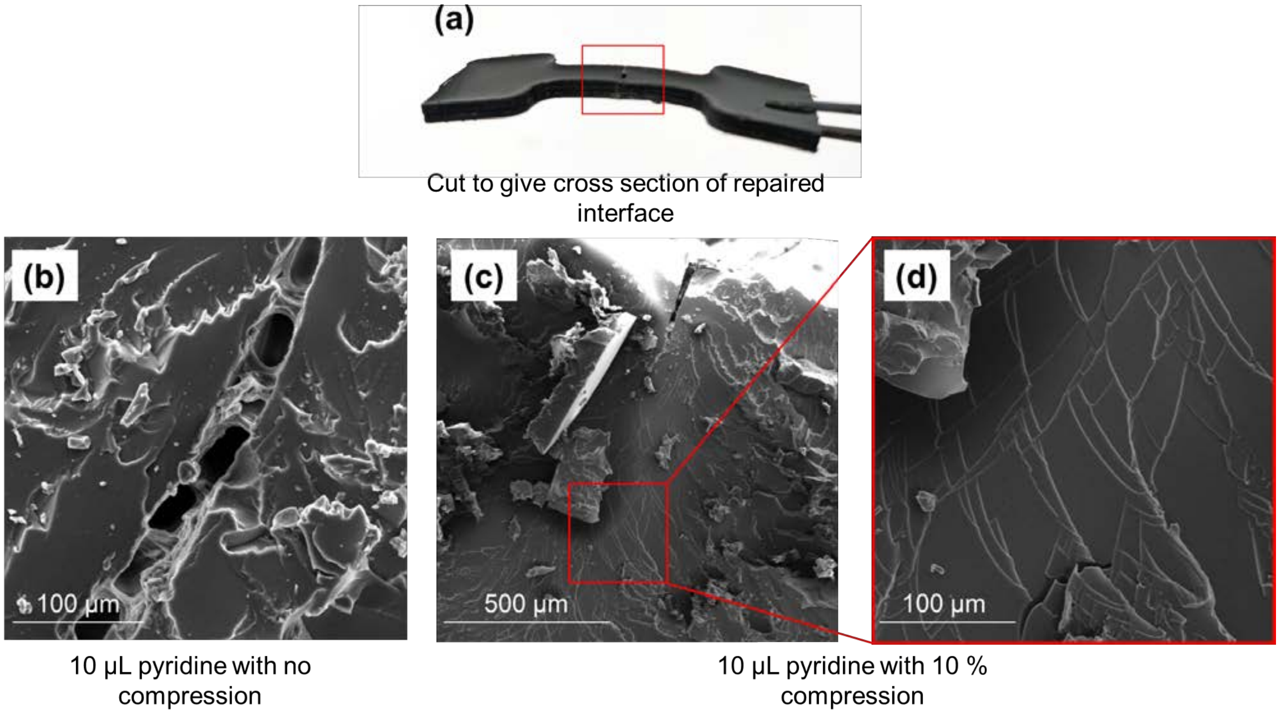

SEM images of the cross section of the interface. (a) Image of repaired polymer piece highlighting reacted interface. (b) SEM image of the cross section of repaired interface with no compression. Pieces were left to repair for 24 hours with 10 µL pyridine. Clear gaps in the polymer are seen where repair did not occur. (c) SEM image of the cross section of the repaired interface with 10 % compression. Pieces were left to repair for 24 hours with 10 µL pyridine. The repaired interface appears largely as a monolithic polymer. (d) magnified image from cross section in (c) showing completely repaired interface.

The presence of the catalysts in the polymer was analyzed using EDX. The polymer does not contain any phosphorus or nitrogen so the presence of the tributylphosphine and pyridine can be determined by tracing these atoms respectively. As can be seen in the images below, pyridine could

not be detected using EDX as no nitrogen atoms were visible at the interface. This is believed to be due to the volatility of pyridine, therefore, this method could be considered traceless if the pyridine is evaporated. Phosphorus could be found at the repaired interface on both sides of the damaged area and in the reacted polymer bridging the interface.

EDX mapping of cross section of interface repaired with 10 µL of pyridine. The repaired region is indicated by the red box. The repaired version appears as bulk polymer and no nitrogen was detected, indicating pyridine is traceless in the polymer repair:

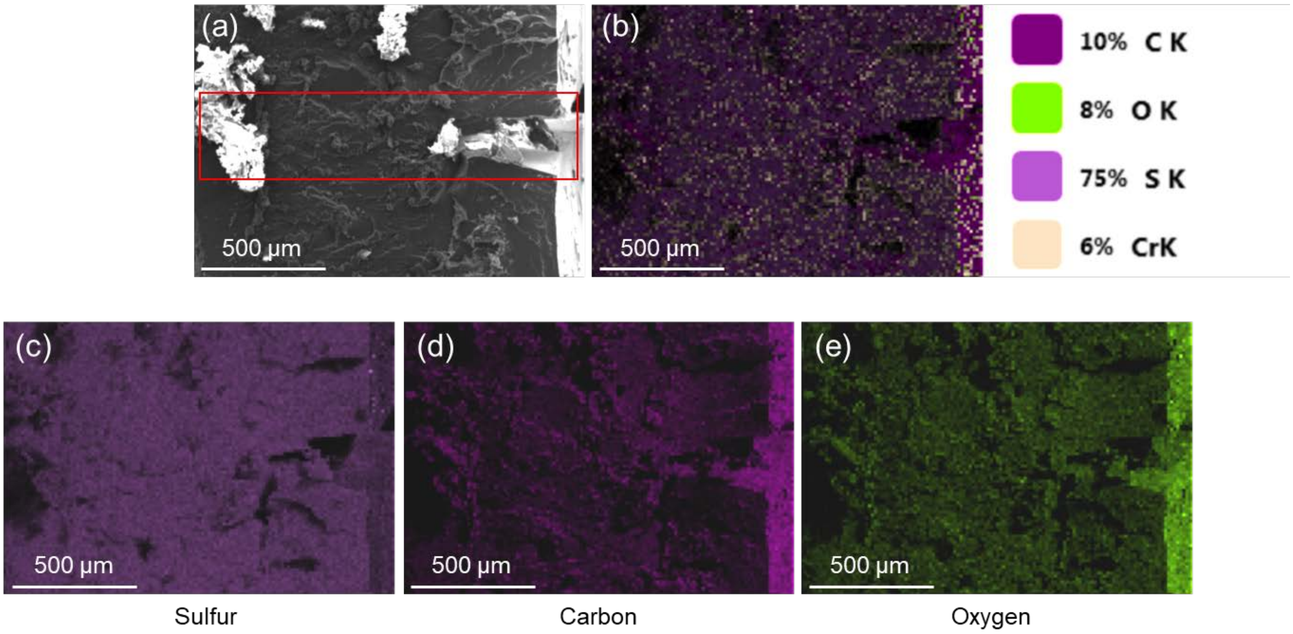

EDX mapping of cross section of interface repaired with 1 µL of tributylphosphine. The repaired region is indicated by the red box. The phosphorous from the tributylphosphine (and tributylphosphine sulfide product) can be detected at the interface and in the bulk polymer. This result shows that the phosphine reacts and migrates through the bulk polymer.

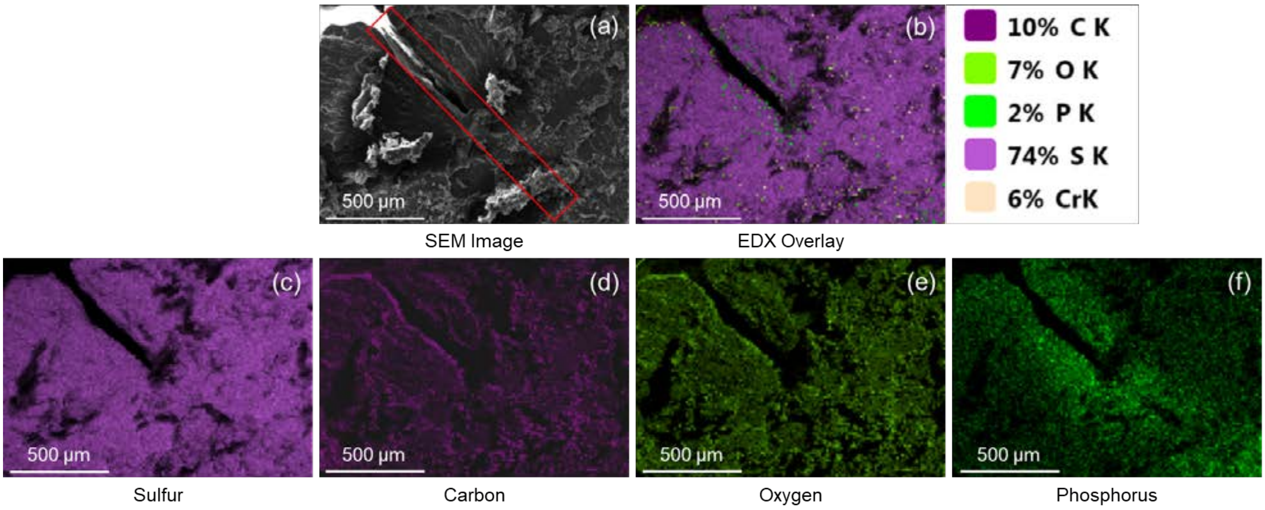

Mechanistic studies of polysulfide metathesis

Extracted products formed after treating polymer with tributylphosphine or pyridine (GC-MS analysis)

Tributylphosphine (73 µL) and pyridine (73 µL) were applied separately to the top of two undamaged polymer dogbone pieces. The tributylphosphine and pyridine-treated polymer pieces were left to react for 24 hours. After this time, the polymer pieces were cut into small pieces with a scalpel and added to 10 ml of chloroform in 25 ml glass vials and left for another hour. The polymer did not dissolve in the chloroform but floated on the surface. After an hour, the polymer was filtered from the chloroform and 1 ml of the chloroform solution was analysed directly using GC-MS. An agilent GC-MS with a single quadrupole mass spectrometer was used for all GC-MS experiments. An injection volume of 1 µL with a split ratio of 60:1 was used for all injections. An initial temperature of 60 °C was used which increased at a rate of 20 degrees per minute to 280 °C, where it was held for an additional two minutes. This gave a total run time of thirteen minutes. A HP-5MS 5 % Phenyl Methyl Silox column was used with dimensions of 29.4 m x 250 µm x 0.25 µm using helium gas with a flow rate of 1.2 ml/ min as the mobile phase. Controls of tributylphosphine and pyridine in chloroform were also run along with a control of the polymer in chloroform without any catalyst. No unreacted tributylphosphine was observed when reacted with the polymer, which has a retention time of approximately 5.6 mins using this method. This indicates that the tributylphosphine is reacting to completion to form the corresponding phosphineoxide or phosphinesulfide. The only other small molecule observed was a cyclic trisulfide byproduct formed from the reaction of sulfur and DCPD.

GC trace of extracted products after polymer treatment with tributylphosphine. The major product was the terminated inducer, tributylphosphine sulfide:

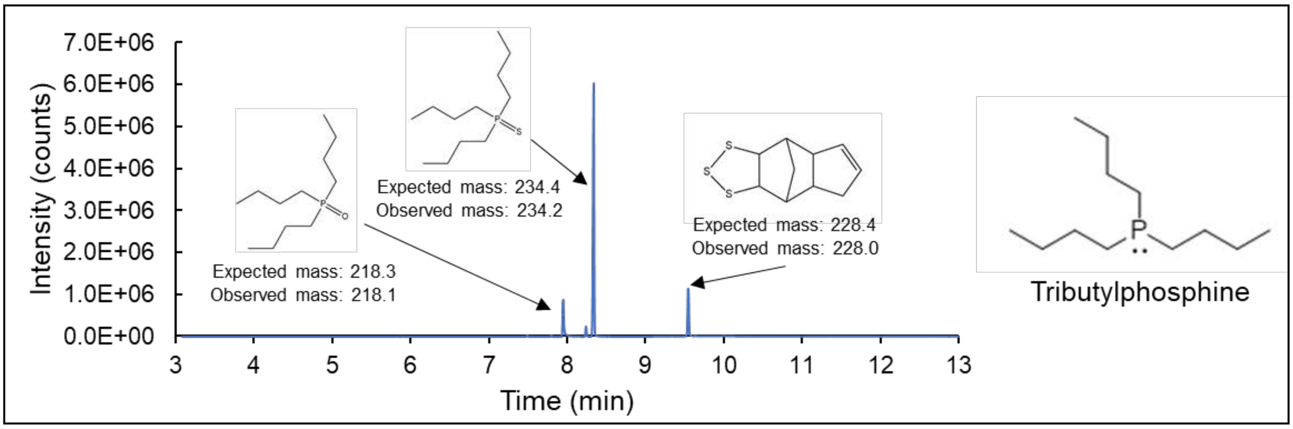

GC trace of extract after treating polymer with pyridine. With this method no pyridine was detected because of its low boiling point.

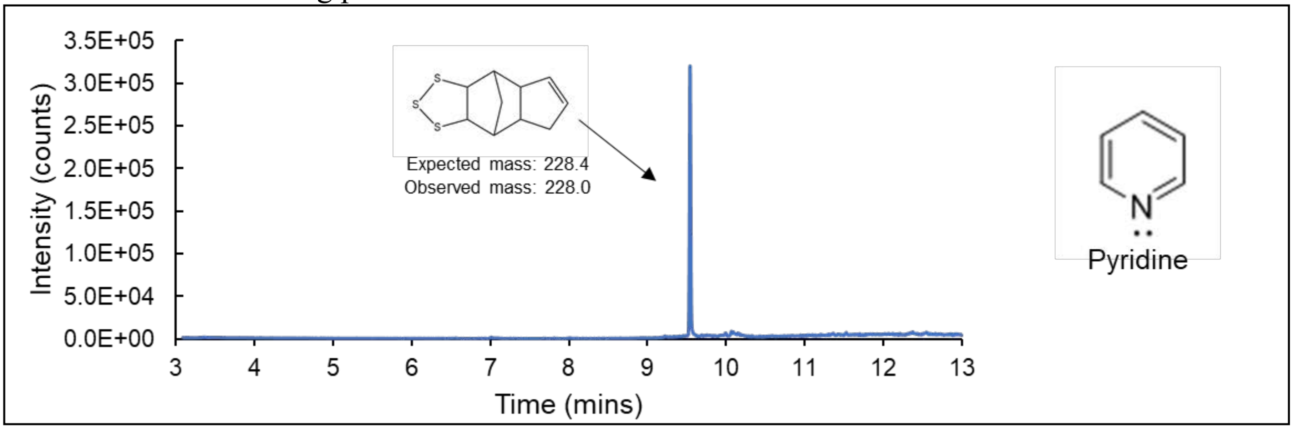

GC trace of extracted products from a polymer treated with neither pyridine nor tributylphosphine. The only compound detected was the trace byproduct formed during the polymerization from the reaction of sulfur and DCPD.

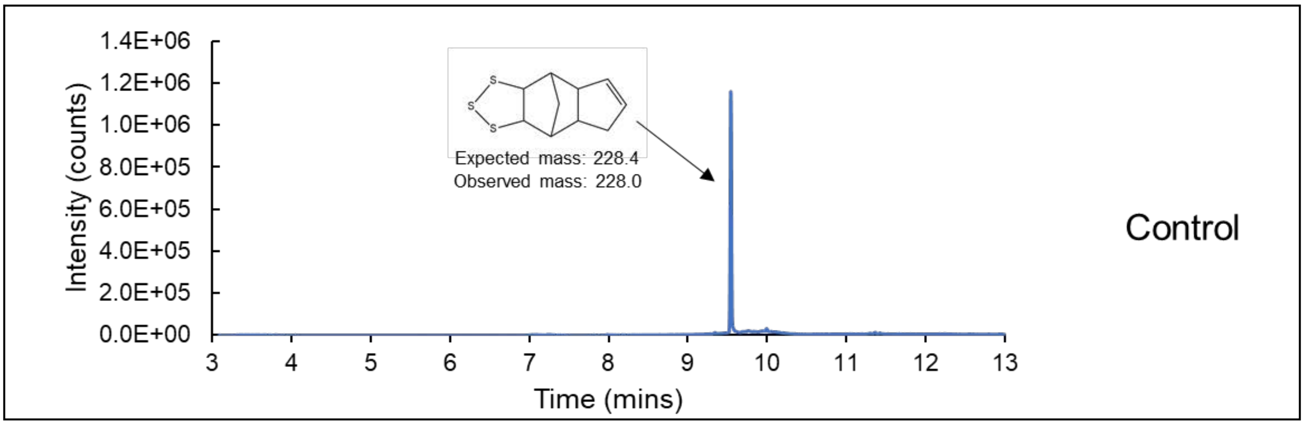

The GC method was altered such that the pyridine could be resolved from the solvent to determine if the sulfide or oxide termination species are formed in the reaction with pyridine. Using the method described above, pyridine had a retention time of less than three minutes and would be eluted from the column before the solvent delay of the detector. The method was altered to ensure that the pyridine had a retention time of greater than three minutes. The final method maintained the same column, injection volume, split ratio, mobile phase and gas flow rate but altered the initial temperature and the temperature ramp rate. An initial temperature of 40 °C was used and was held for two minutes. The temperature was then increased to 45 °C with a ramp rate of 5 °C/ min. After this point, the temperature was ramped to 260 °C at a ramp rate of 20 °C/ min. This gave a total run time of 13.75 minutes. Using this method, the pyridine could be resolved with a retention time of 3.25 mins. No oxide or sulfide species was observed, showing only unreacted pyridine.

GC trace of extracted products after polymer treatment with pyridine. Unreacted pyridine was detected as well as the trace byproduct formed during the polymerization from the reaction of sulfur and DCPD.

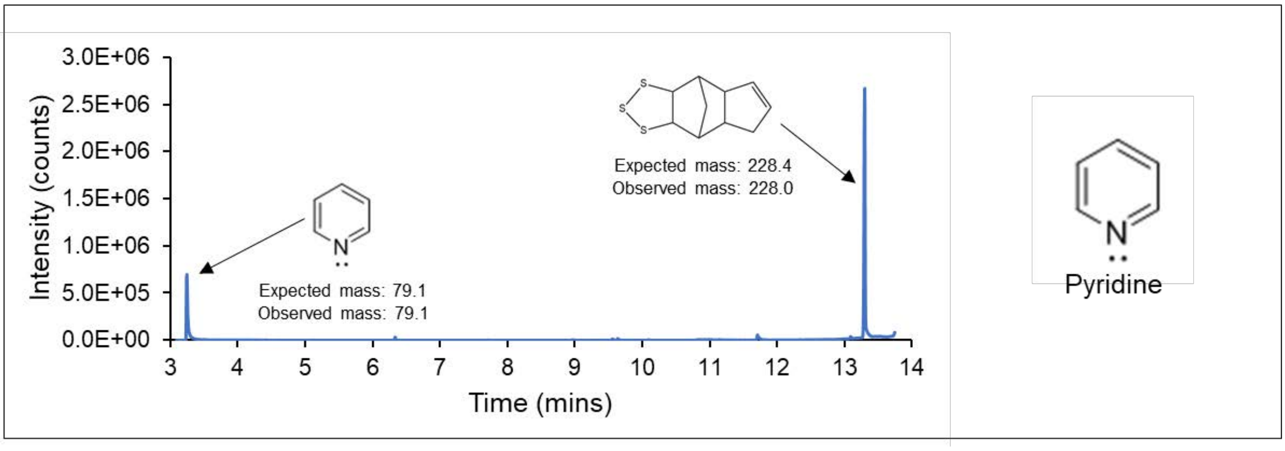

EDX of polymer treated with pyridine or tributylphosphine (before and after chloroform wash)

The same reaction as above was repeated for four additional pieces of polymer. Two were treated with tributylphosphine and two with pyridine. All pieces were left for 24 hours. One piece reacted with tributylphosphine and one piece reacted with pyridine were added to separate vials containing 10 ml of chloroform for an additional hour. After this time, they were removed and left for another hour to dry. All pieces were then coated in 20 nm of chromium and investigated using EDX. No nitrogen was visible in either of the pyridine pieces, indicating that it had evaporated. Phosphorus was only observed in the piece that was not added to chloroform. This indicates that no tributylphosphine (or any derivatives) became incorporated into the polymer and all tributylphosphine sulfide was removed by the chloroform.

SEM and EDX images of pyridine treated polymer without extraction by chloroform. (a) SEM image of area analysed by EDX. (b) EDX overlay mapping. (c) EDX spectrum for polymer. No nitrogen was detected so no pyridine or pyridine derivative remained bonded to the polymer.

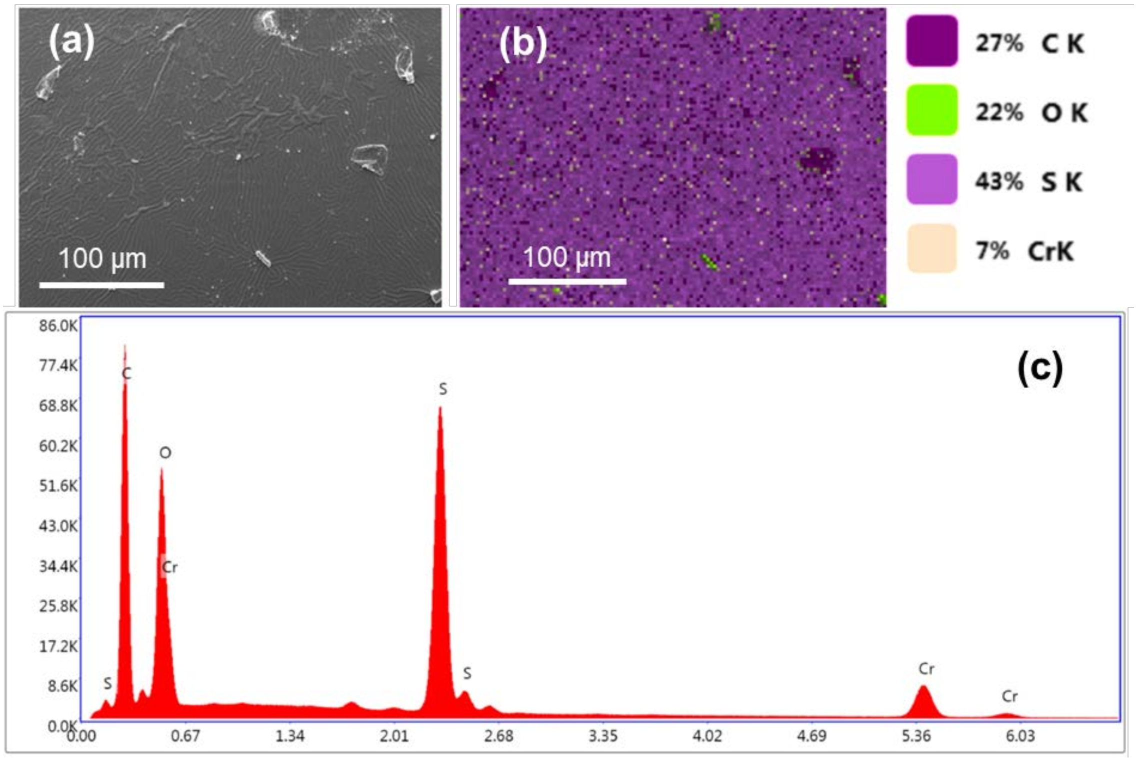

SEM and EDX images of pyridine treated polymer after extraction with chloroform. (a) SEM image of area analysed by EDX. (b) EDX overlay mapping. (c) EDX spectrum for polymer. No nitrogen or pyridine derivatives were detected:

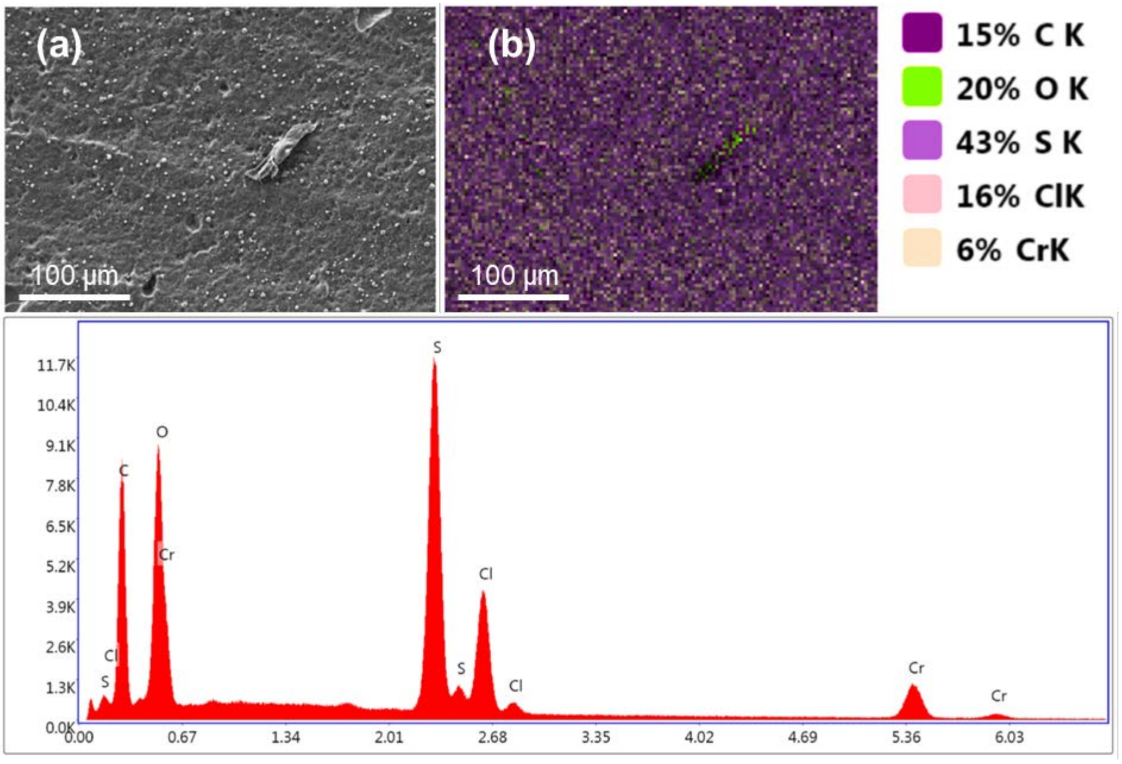

EDX of tributylphosphine treated polymer without extraction with chloroform. (a) SEM image of area analysed by EDX. (b) EDX overlay mapping. (c) EDX spectrum for polymer. The phosphorous of the tributylphosphine and/or tributylphosphine sulfide is clearly visible on the polymer surface:

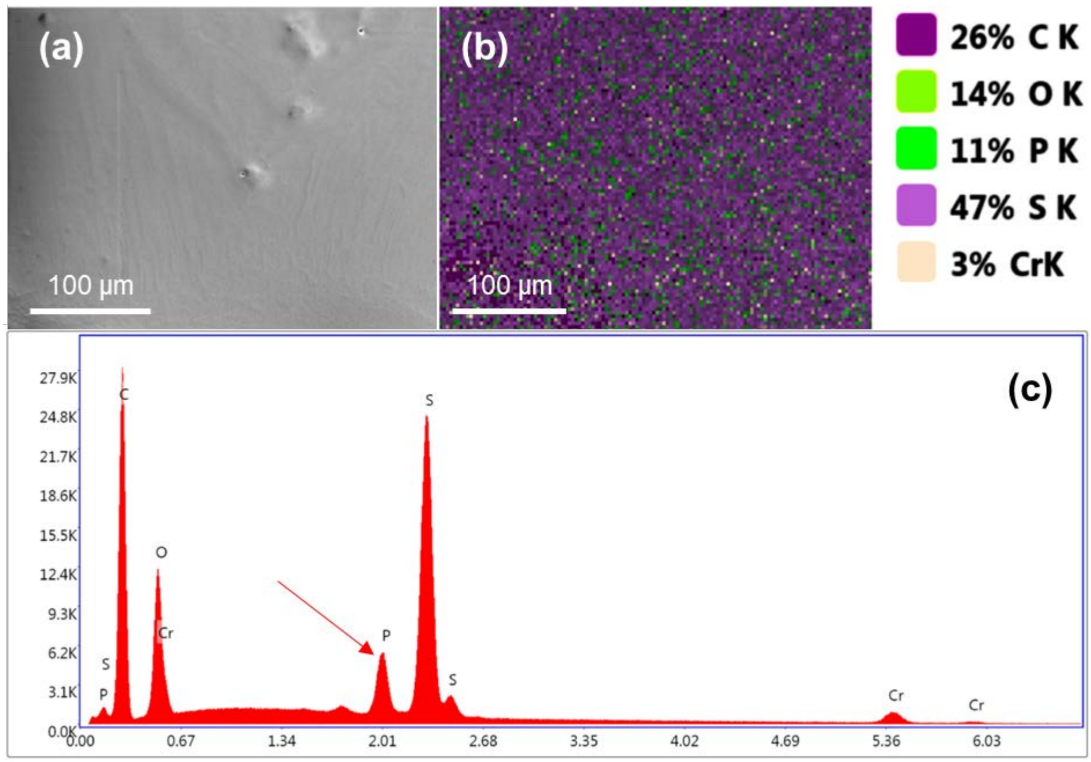

Tributylphosphine treated polymer after extraction with chloroform. (a) SEM image of area analysed by EDX. (b) EDX overlay mapping. (c) EDX spectrum for polymer. Very little phosphorous was detected, indicating the tributylphosphine and tributylphosphine sulfide were efficiently extracted from the polymer surface:

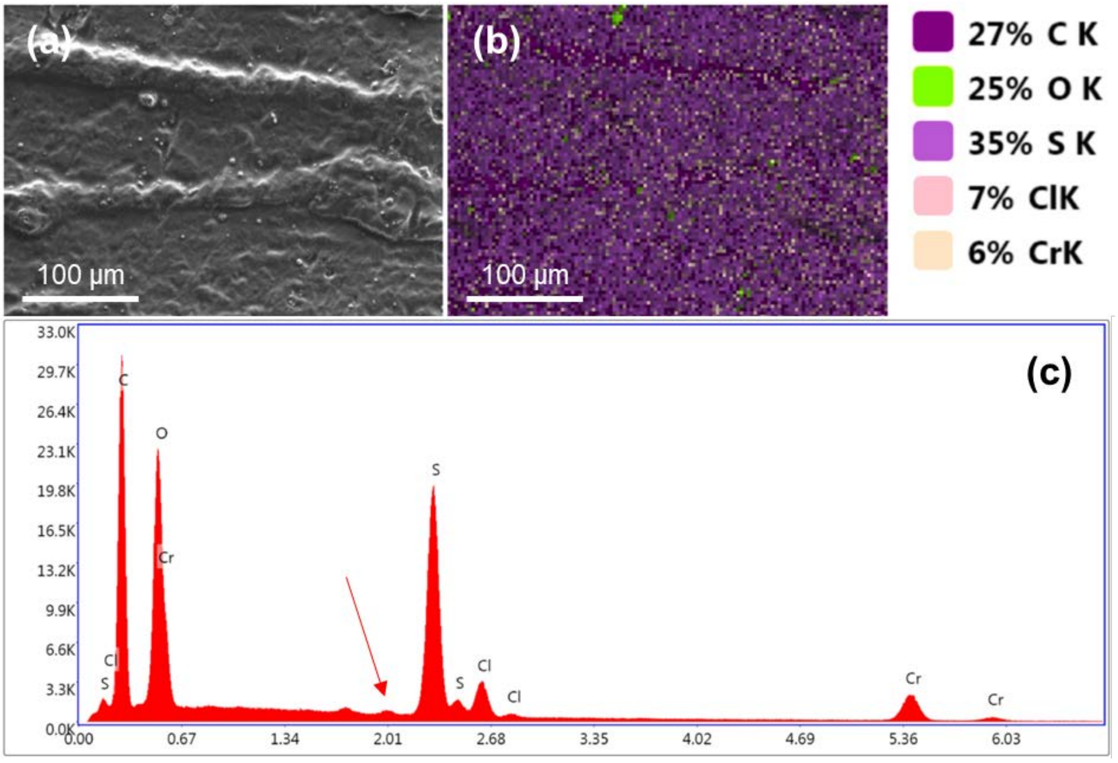

NMR of products formed when polymer treated with pyridine or tributylphosphine

Pyridine (73 μL) was added to the polymer and incubated for 24 hours. The polymer was then placed in $CDCl_3$ (10 mL) to extract the products. A 500 μL aliquot of this solution was analyzed by ^1H NMR spectroscopy. Unreacted pyridine was detected, indicating it is not consumed in its reaction with the polymer. Other peaks correspond to lower molecular weight and soluble fractions of the polymer and also the cyclic trisulfide byproduct formed from the reaction of DCPD and sulfur.

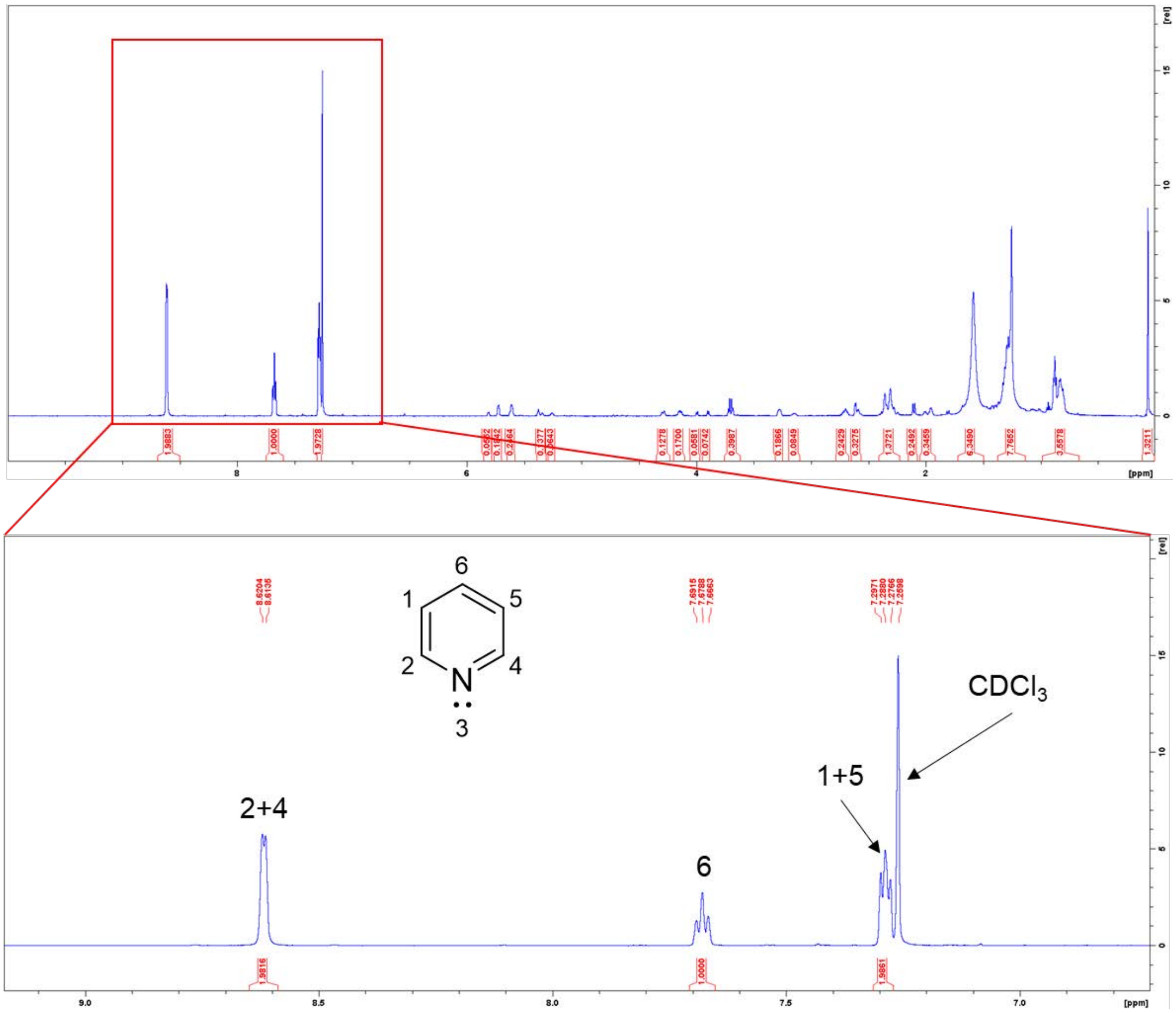

Tributylphosphine (73 μL) was added to the polymer and incubated for 24 hours. The polymer was then placed in $CDCl_3$ (10 mL) to extract the products. A 500 μL aliquot of this solution was analyzed by ^{31}P NMR spectroscopy. The major products were tributylphosphine sulfide and tributylphosphine oxide (peaks at 48.8 and 48.9 ppm), which is consistent with the GC-MS analysis on page S27. No unreacted tributylphosphine was detected (-32.5 ppm).

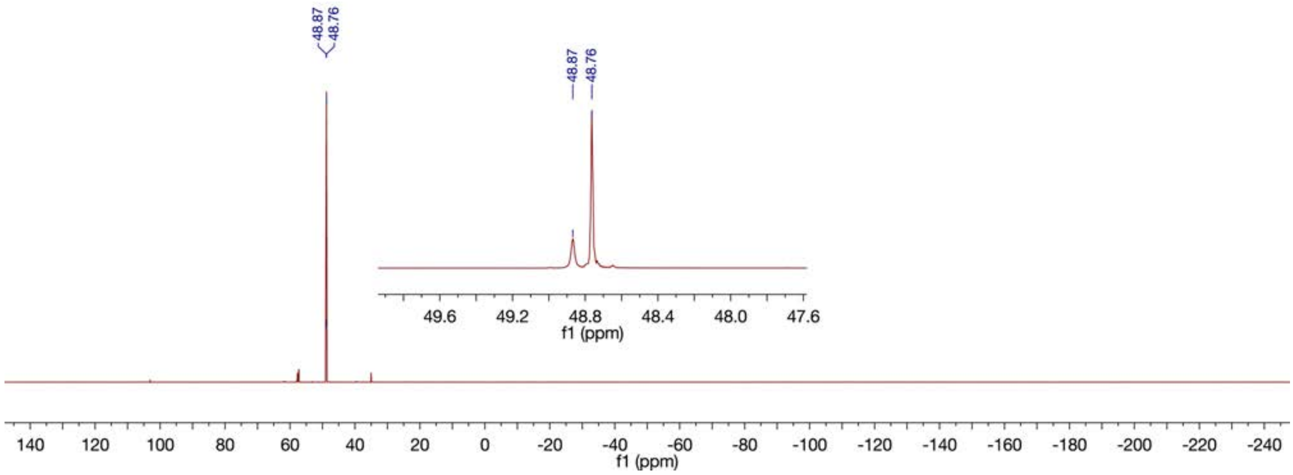

Disulfide crossover experiments using pyridine and tribuytlphosphine catalysts:

GC-MS vials containg 115 mM dimethyl disulfide, 115 mM dipropyl disulfide and 115 mM tributylphosphine or pyridine in chloroform were prepared, along with a control which did not contain pyridine or tributylphosphine. Another sample was prepared which used pyridine as a solvent for the reaction. This sample was prepared as it more closely matched the conditions used in the reaction with the polymer as pyridine is applied neat to the polymer surface. It was not possible to have a sample in neat tributylphosphine as it had a longer retention time then methyl propyl disulfide. Therefore, it would not be possible to determine if the crossover reaction had occurred as the methyl prophyl disulfide would be eluted during the solvent delay of the detector. The same GC method from the pyridine reaction products extraction was used. This method had an initial temperature of 40 °C which was held for two minutes. The temperature was then increased to 45 °C with a temperature ramp rate of 5 °C/min. The temperature was then increased to 260 °C at a temperature ramp rate of 20 °C This lead to a total run time of 13.75 minutes. A solvent delay of 3 minutes was maintained to prevent overloading the detector. Using this method, pyridine was eluted at 3.25 minutes while dimethyl disulfide was eluted at 3.35 minutes. All samples were ran twelve times over 24 hours to trace the reaction over time. GC traces and control experiments are shown on the following pages.

Experiment 1: Negative control with no pyridine and no tributylphosphine.

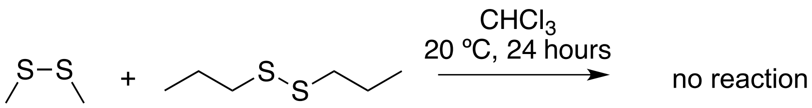

Dimethyl disulfide (10.2 µL, 0.115 mmol) was added to a GC-MS vial by micropipette, followed by chloroform (971.8 µL). Finally, dipropyl diisulfide (18.0 µL, 0.115 mmol) was added to the solution by micropipette and the vial was capped. The solution was analyzed by GC-MS several times over a 24 hour period. No reaction was observed and only unreacted dimethyl disulfide (calculated = 94.2 Da; found = 94.2 Da) and dipropyl trisulfide (calculated = 150.3 Da; found = 150.3 Da) were detected. This result shows that the S-S metathesis does not occur spontaneously under these conditions and that a catalyst is required.

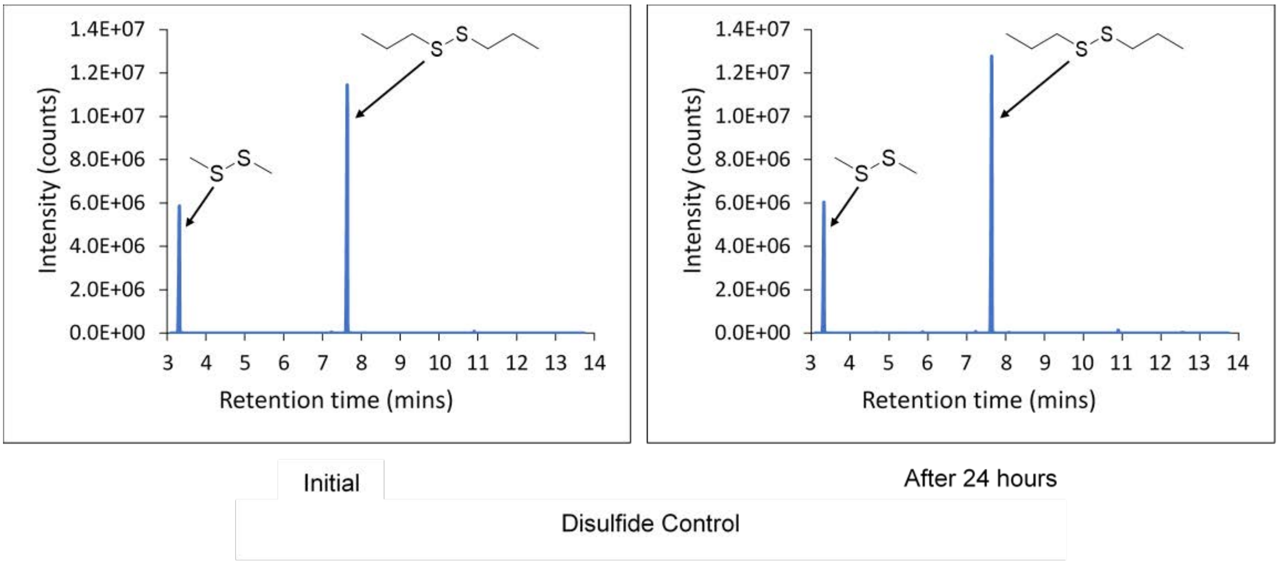

Peak Number	*Retention Time (min)*	*Calculated mass (Da)*	*Observed mass (Da)*	*Identity*	*Structure*
1	*3.35*	*94.2*	*94.2*	Dimethyl disulfide	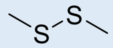
2	7.63	150.3	150.3	Dipropyl disulfide	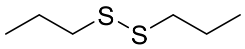

Experiment 2: Reaction of disulfides with tributylphosphine

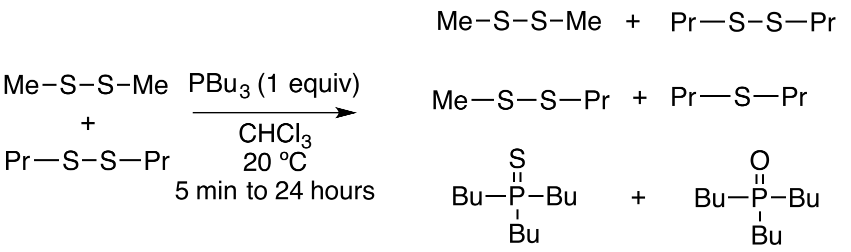

Dimethyl disulfide (10.2 µL, 0.115 mmol) was added to a GC-MS vial by micropipette, followed by chloroform (943.5 µL). Next, dipropyl diisulfide (18.0 µL, 0.115 mmol) was added to the solution by micropipette. Finally, tributylphosphine (28.3 µL, 0.115 mmol) was added and the vial was capped. Rapid reaction and metathesis was observed, with the ratio between the two added disulfides and the crossover disulfide (methyl propyl disulfide) reacting equilibrium within 5

minutes and remaining relatively constant. Over 24 hours, a dipropyl sulfide and tributylphosphine sulfide peak emerged. This is indicative of desulfurization. Note that other sulfides likely formed but had a retention time of less than 3 minutes and were therefore undetected. The tributylphosphine peak decreased over 24 hours while the tributylphosphine oxide and sulfide peaks increased. As there was significant sulfur bond metathesis without desulfurization peaks being observed, it can be concluded that the desulfurization is a slower process and not required for S-S metathesis.

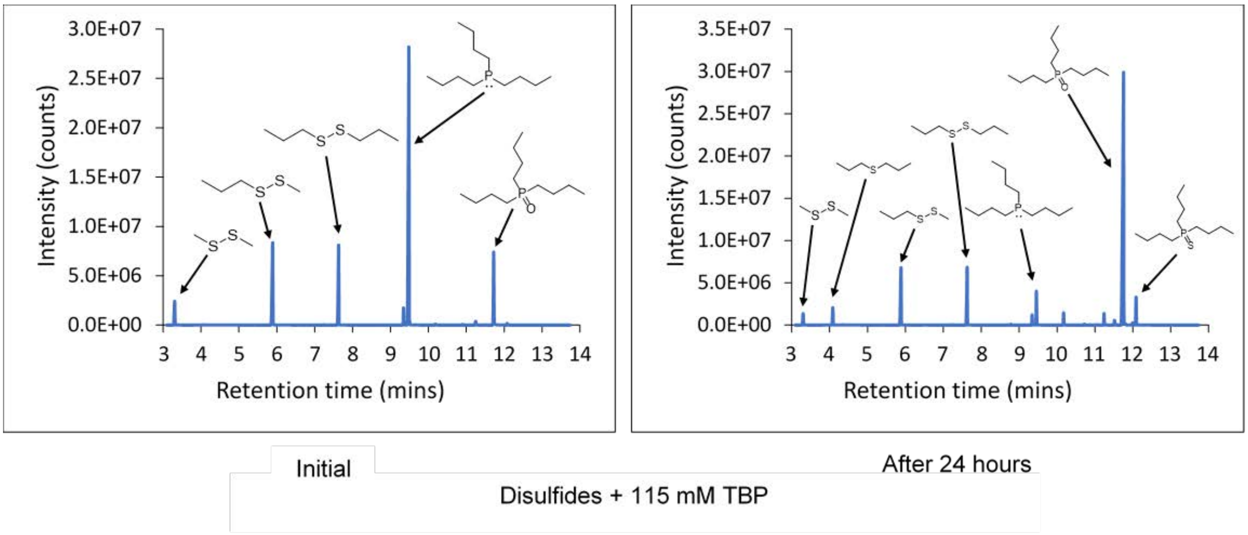

Peak Number	*Retention Time (min)*	*Calculated mass (Da)*	*Observed mass (Da)*	*Identity*	*Structure*
1	*3.35*	*94.2*	*94.2*	Dimethyl disulfide	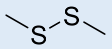
2	*4.07*	*118.2*	*118.1*	Dipropyl sulfide	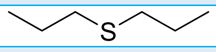
3	*5.90*	*122.2*	*122.1*	Methyl propyl disulfide	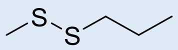
4	7.63	150.3	150.3	Dipropyl disulfide	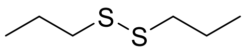
5	9.48	202.3	202.1	Tributylphosphine	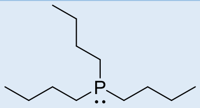
6	11.72	218.3	218.2	Tributylphosphine oxide	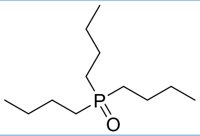
7	12.08	234.4	234.2	Tributylphosphine sulfide	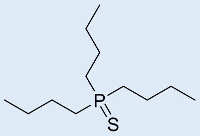

A mechanistic rationale for the observed products is shown on the next page.

Mechanistic rational for the crossover product formation:

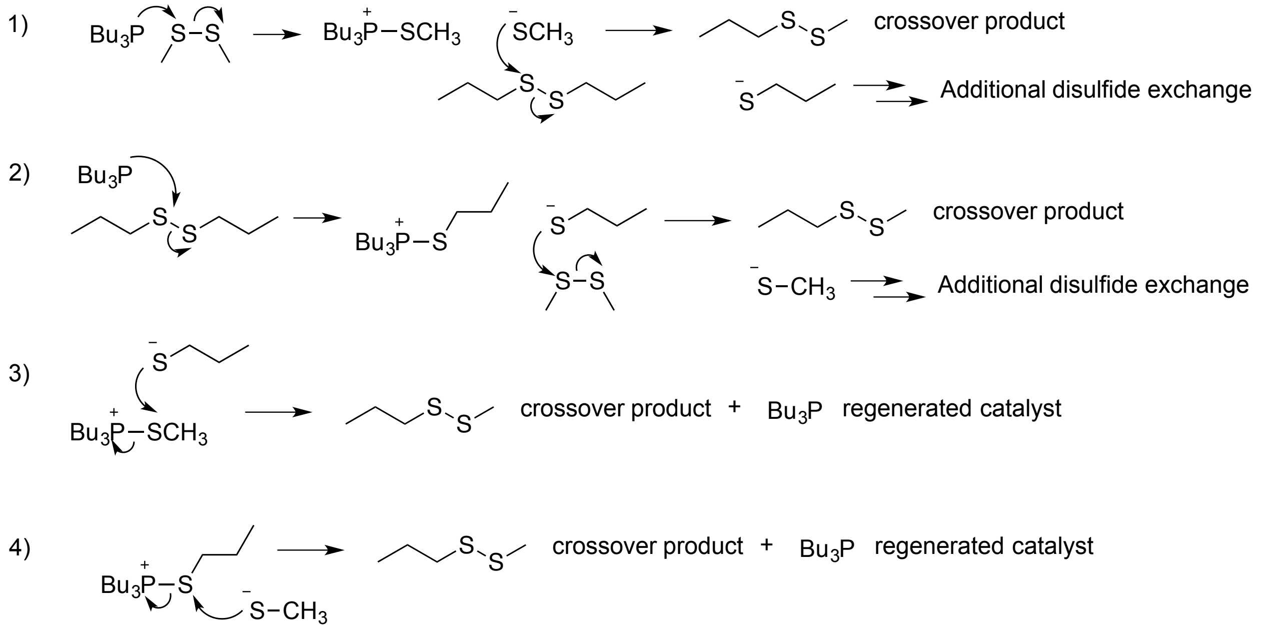

Mechanistic rationale for the desulfurization and tributylphosphine sulfide formation:

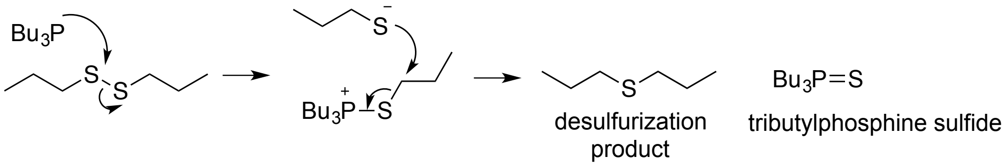

The tributylphosphine oxide can form from air oxidation of tributylphosphine or hydrolysis of tributylphosphine oxide.

Experiment 3: Attempted reaction of disulfides with pyridine in chloroform

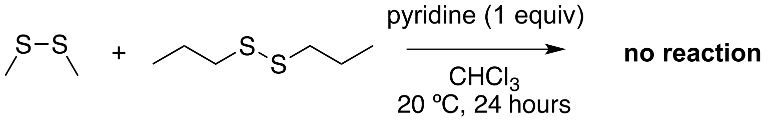

Dimethyl disulfide (10.2 µL, 0.115 mmol) was added to a GC-MS vial by micropipette, followed by chloroform (962.6 µL). Next, dipropyl disulfide (18.0 µL, 0.115 mmol) was added to the solution by micropipette. Finally, pyridine (9.2 µL, 0.115 mmol) was added and the vial was capped. No reaction was observed and only unreacted pyridine, dimethyl disulfide and dipropyl disulfide were detected. This experiment indicates that pyridine does not catalyze the S-S metathesis in disulfides at this concentration and temperature.

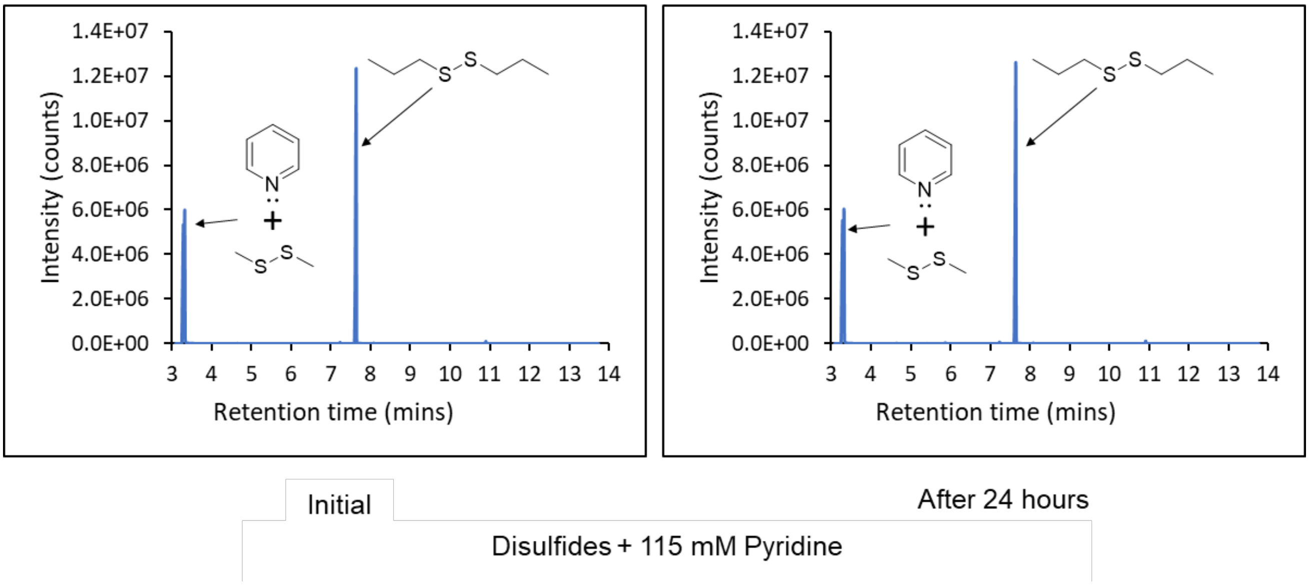

Peak Number	*Retention Time (min)*	*Calculated mass (Da)*	*Observed mass (Da)*	*Identity*	*Structure*
1	*3.25*	*79.1*	*79.1*	Pyridine (not fully resolved)	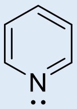
2	*3.35*	*94.2*	*94.2*	Dimethyl disulfide (not fully resolved)	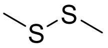
3	7.63	150.3	150.3	Dipropyl disulfide	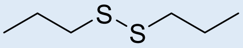

Experiment 4: Attempted disulfide exchange in neat pyridine

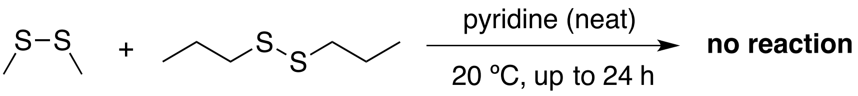

In the sample with neat pyridine as a solvent, the solvent delay had to be increased to 5 minutes to prevent overloading the detector as pyridine had a retiontion time of 3.25 minutes. This meant that dimethyl disulfide was not visible. However, the crossover product, methylpropyl disulfide, would have been visible using the method, with a retention time of 5.89 minutes. Dimethyl disulfide (10.2 µL, 0.115 mmol) was added to a GC-MS vial by micropipette, followed by pyridine (971.8 µL). Finally, dipropyl disulfide (18.0 µL, 0.115 mmol) was added to the solution by micropipette and the vial was capped. This sample showed only the dipropyl disulfide peak, indicating that pyridine did not catalyze disulfide metathesis under these conditions, even at high concentrations.

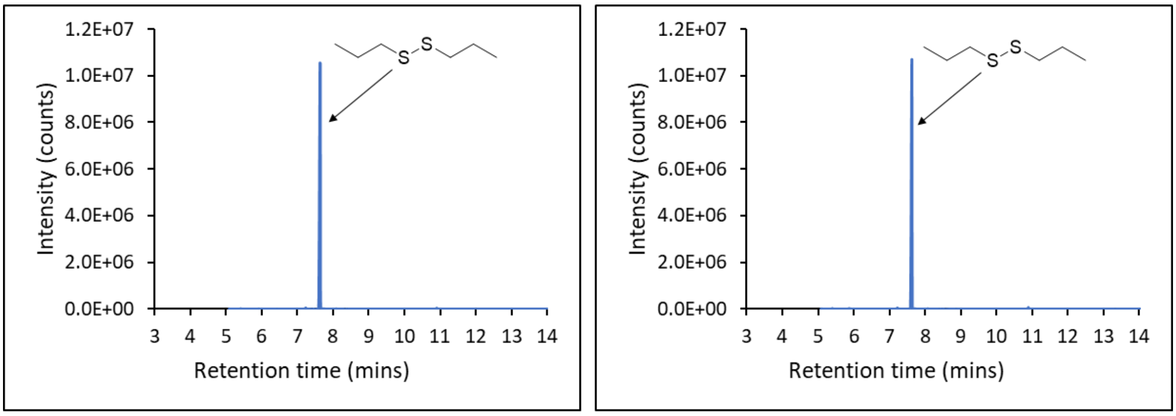

Initial | After 24 hours

Disulfides in neat pyridine

Peak Number	***Retention Time (min)***	***Calculated mass (Da)***	***Observed mass (Da)***	***Identity***	***Structure***
1	7.63	150.3	150.3	Dipropyl disulfide	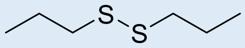

20 μL of the solution in neat pyridine was removed by micropipette and diluted 50 times to 1 mL using chloroform. This sample was then tested by GCMS so the same method as other samples could be used. Only pyridine and dipropyl disulfide peaks were visible, however, the right side of the pyridine peak showed a mass spectrum for dimethyl disulfide, indicating that there are two peaks that are not resolved. The absence of the methyl propyl disulfide peak further indicates that pyridine does not induce sulfur bond metathesis of disulfides.

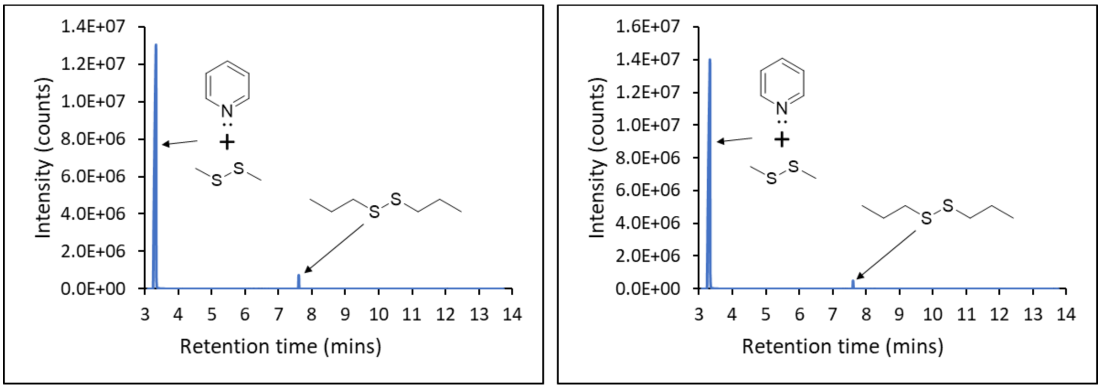

Initial | After 24 hours

Disulfides in neat pyridine diluted 50 times

Peak Number	***Retention Time (min)***	***Calculated mass (Da)***	***Observed mass (Da)***	***Identity***	***Structure***
1	*3.25*	*79.1*	*79.1*	Pyridine	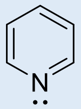
2	*3.35*	*94.2*	*94.2*	Dimethyl disulfide (not fully resolved)	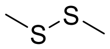
3	7.63	150.3	150.3	Dipropyl disulfide	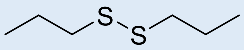

Trisulfide crossover experiments using pyridine and tributylphosphine catalysts

Dimethyl trisulfide and dipropyl trisulfide were used in a crossover experiment using GC-MS. The same concentration of trisulfides were used as the disulfide crossover experiments. 115 mM dimethyl trisulfide and 115 mM dipropyl trisulfide were used for all experiments with a total reaction volume of 1 mL. The samples were analyzed by GC-MS 12 times each over a 24 hour period. The column had an initial temperature of 60 °C which was held for three minutes. The temperature was then ramped at a rate of 20 °C/min to 300 °C.

Experiment 1: Negative control with no pyridine and no tributylphosphine

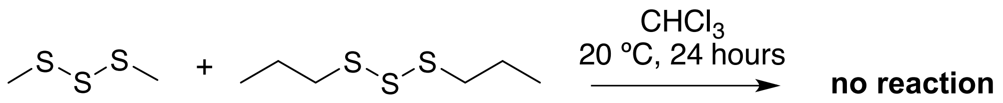

Dimethyl trisulfide (12.1 µL, 0.115 mmol) was added to a GC-MS vial by micropipette, followed by chloroform (970 µL). Finally, di-*n*-propyl trisulfide (17.9 µL, 0.115 mmol) was added to the solution by micropipette and the vial was capped. The solution was analyzed by GC-MS several times over a 24 hour period. No reaction was observed and only unreacted dimethyl trisulfide (calculated = 126.3 Da; found = 126.1 Da) and di-*n*-propyl trisulfide (calculated = 182.4 Da; found = 182.2 Da) were detected. This control experiment indicates that S-S metathesis does not occur spontaneously under these conditions and a catalyst is required.

GC traces are shown below for the first analysis (<5 min incubation) and also after 24 hours:

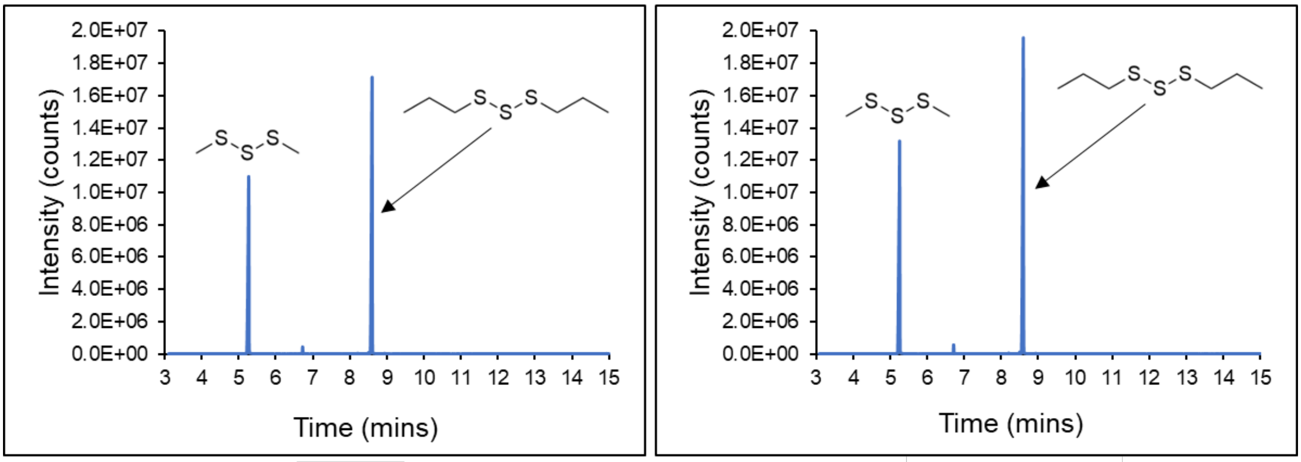

Initial | After 24 hours

Trisulfide Control

Peak Number	*Retention Time (min)*	*Calculated mass (Da)*	*Observed mass (Da)*	*Identity*	*Structure*
1	5.256	126.3	126.2	Dimethyl Trisulfide	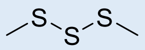
2	6.778	150.3	150.3	Dipropyl disulfide (impurity)	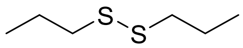
3	8.58	182.4	182.3	Dipropyl Trisulfide	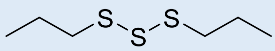

Experiment 2: Reaction of trisulfides with tributylphosphine

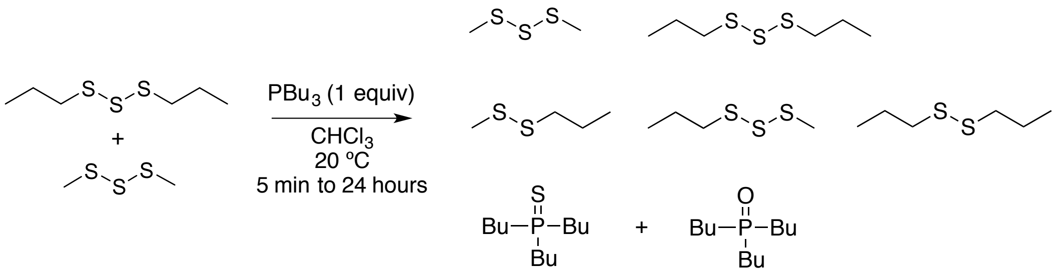

Dimethyl trisulfide (12.1 µL, 0.115 mmol) was added to a GC-MS vial by micropipette, followed by chloroform (941.7 mL). Next, dipropyl trisulfide (17.9 µL, 0.115 mmol) was added to the solution by micropipette. Finally, tributylphosphine (28.3 µL, 0.115 mmol) was added and the vial was capped. The solution was analyzed by GC-MS several times over a 24 hour period. Rapid reaction and S-S metathesis was observed in < 5 minutes, with little change over 24 hours. Disulfides and tributylphosphine sulfide were observed from first time period. This indicates that desulfurization occurs rapidly under these conditions. A mechanistic rationale for the formation of the observed products is shown on the next page.

GC traces are shown below for the first analysis (<5 min incubation) and also after 24 hours:

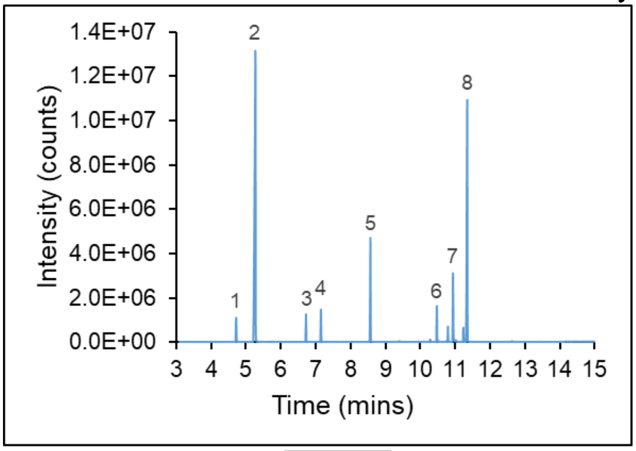

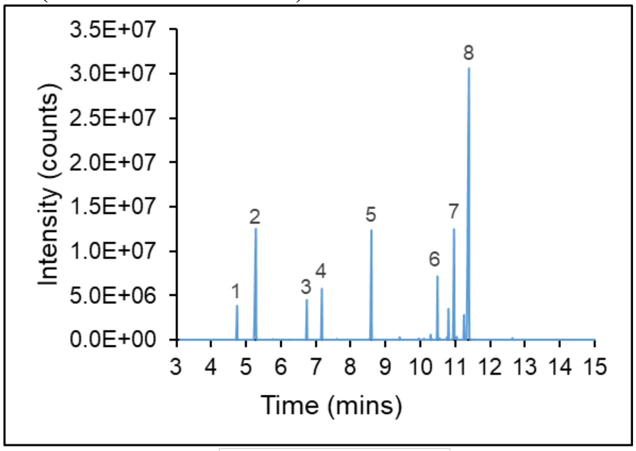

Initial After 24 hours

Trisulfides + 115 mM tributylphosphine

Peak Number	*Retention Time (min)*	*Calculated mass (Da)*	*Observed mass (Da)*	*Identity*	*Structure*
1	4.712	122.2	122.2	Methyl propyl disulfide	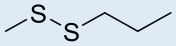
2	5.256	126.3	126.2	Dimethyl Trisulfide	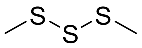
3	6.778	150.3	150.3	Dipropyl disulfide	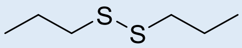
4	7.144	154.3	154.2	methyl propyl trisulfide	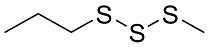
5	8.58	182.4	182.3	Dipropyl Trisulfide	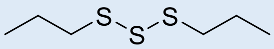
6	10.474	N/A	205.1	Unknown	
7	10.937	218.3	218.2	Tributylphosphine oxide	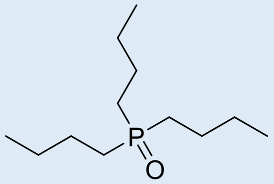

8	11.344	234.4	234.2	Tributylphosphine sulfide	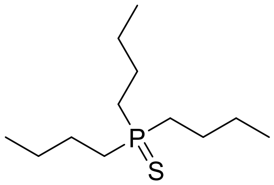

Mechanistic pathways to trisulfide crossover product

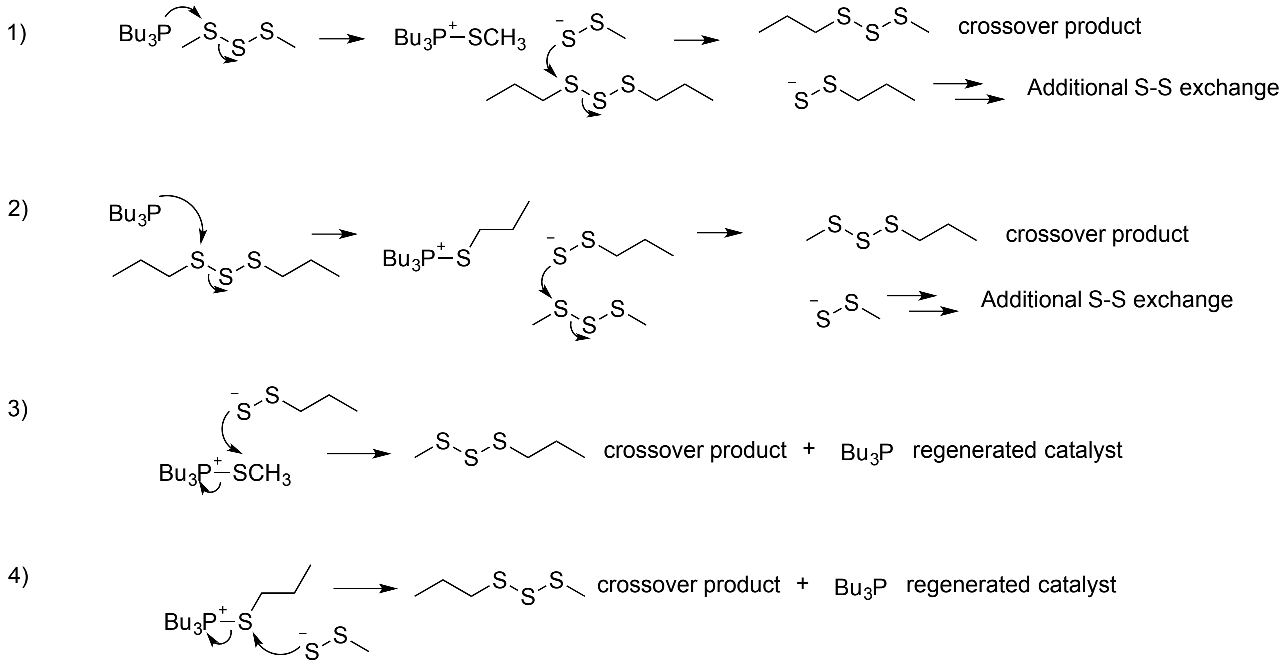

Mechanistic pathways to observed desulfurization products

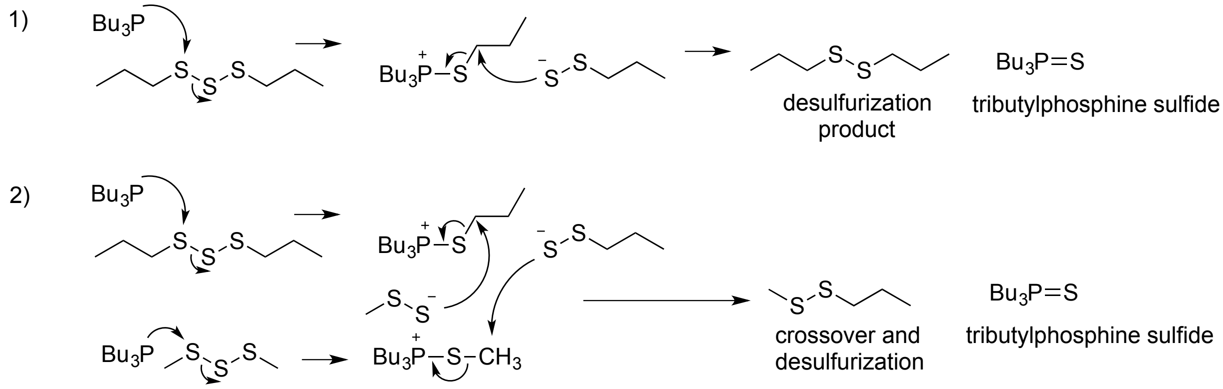

Experiment 3: Attempted reaction of trisulfides with 1 equivalent of pyridine in chloroform

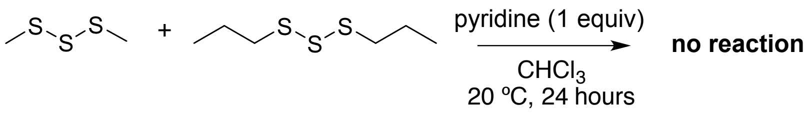

Dimethyl trisulfide (12.1 µL, 0.115 mmol) was added to a GC-MS vial by micropipette, followed by chloroform (960.8 µL). Next, dipropyl trisulfide (17.9 µL, 0.115 mmol) was added to the solution by micropipette. Finally, pyridine (9.2 µL, 0.115 mmol) was added and the vial was capped. The solution was analyzed by GC-MS several times over a 24 hour period. No reaction was observed and only unreacted dimethyl trisulfide and dipropyl trisulfide were detected. This experiment indicates that pyridine does not catalyze the S-S metathesis in trisulfides at this concentration and temperature.

GC traces are shown below for the first analysis (<5 min incubation) and also after 24 hours:

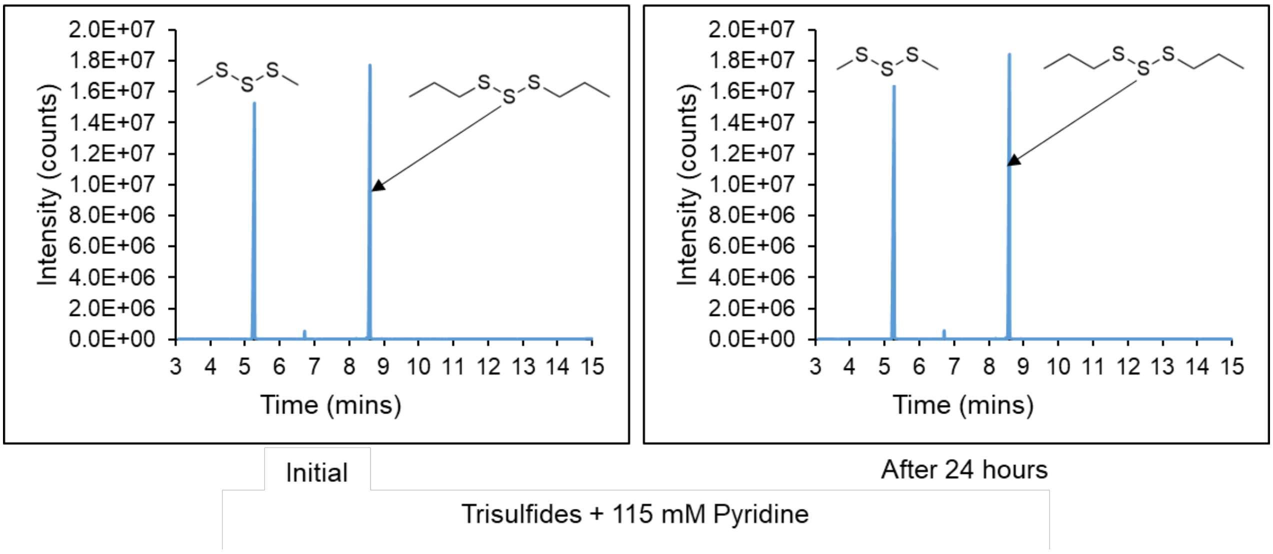

Peak Number	*Retention Time (min)*	*Calculated mass (Da)*	*Observed mass (Da)*	*Identity*	*Structure*
1	5.256	126.3	126.2	Dimethyl Trisulfide	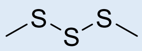
2	6.778	150.3	150.3	Dipropyl disulfide (impurity)	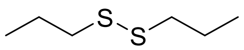
3	8.58	182.4	182.3	Dipropyl Trisulfide	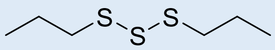

Experiment 4: Reaction of trisulfides in neat pyridine

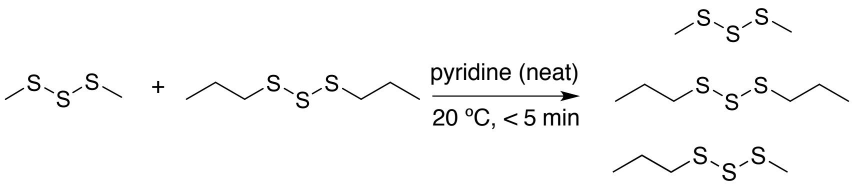

Dimethyl trisulfide (12.1 µL, 0.115 mmol) was added to a GC-MS vial by micropipette, followed by pyridine (970 µL). Finally, dipropyl trisulfide (17.9 µL, 0.115 mmol) was added to the solution by micropipette and the vial was capped. The solution was analyzed by GC-MS several times over a 24 hour period. Rapid crossover via S-S metathesis was observed within 5 minutes. The crossover was indicated by the detection of methylpropyl trisulfide (calculated = 154.3 Da; found = 154.3 Da)

GC traces are shown below for the first analysis (<5 min incubation) and also after 24 hours:

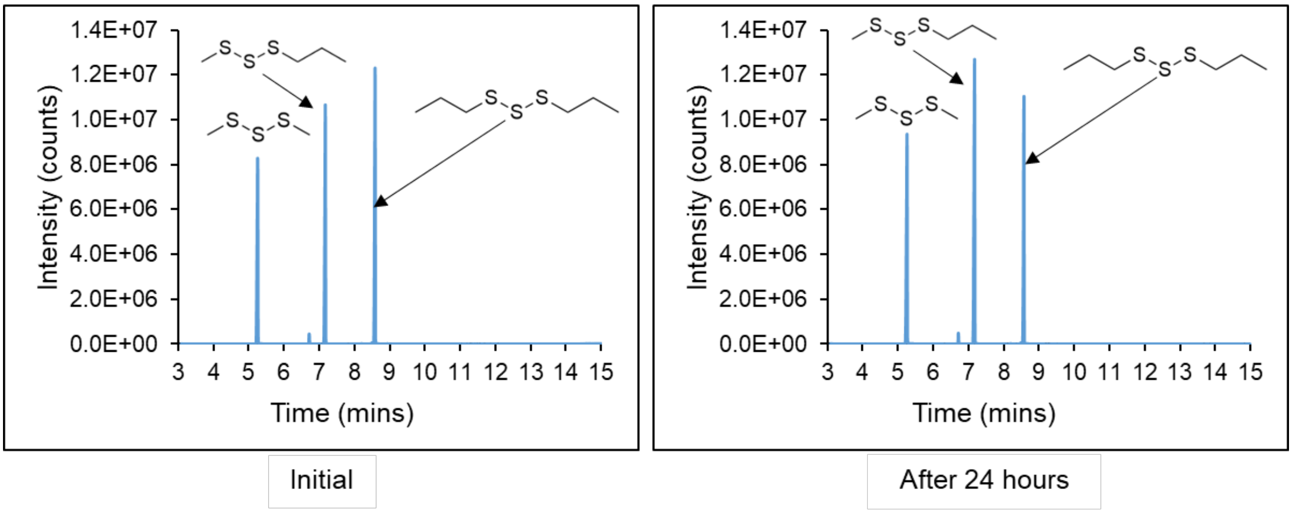

Trisulfides in Pyridine

Peak Number	*Retention Time (min)*	*Calculated mass (Da)*	*Observed mass (Da)*	*Identity*	*Structure*
1	5.256	126.3	126.2	Dimethyl Trisulfide	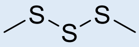
2	6.778	150.3	150.3	Dipropyl disulfide (impurity)	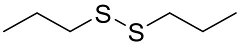
3	7.144	154.3	154.2	methyl propyl trisulfide	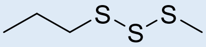
4	8.58	182.4	182.3	Dipropyl Trisulfide	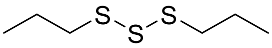

Computational study of pyridine catalyzed S-S metathesis using pyridine as a catalyst

The geometries and harmonic vibrational frequencies of all structures have been obtained from density functional theory (DFT) calculations at the B3LYP-D3BJ/6-31G(2df,p) level of theory.[4-6] Empirical D3 dispersion corrections[7] are included using the Becke–Johnson[8] damping potential as recommended previously.[9] Bulk solvent effects in pyridine in the geometry and frequency calculations were included using the charge-density-based SMD continuum solvation model,[10] with this level of theory denoted SMD(pyridine)-B3LYP/6-31G(2df,p). Zero-point vibrational energies (ZPVEs), enthalpic temperature corrections (H_{298}–H_0), and entropic corrections have been obtained from such calculations. The equilibrium structures were verified to have all real harmonic frequencies and the transition structures to have only one imaginary frequency. The connectivities of the transition and equilibrium structures were confirmed via intrinsic reaction coordinate (IRC) calculations.[11] Gas-phase Gibbs energies at 298 K were obtained using the G4(MP2) variant of the Gaussian-4 (G4) composite thermochemical protocol, using the SMD(pyridine)-B3LYP/6-31G(2df,p) optimized geometries. Relative Gibbs free energies in the text refer to the combination of an SMD(pyridine)-M05-2X/6-31G(d) solvation correction on top of the G4(MP2)//SMD(pyridine)-B3LYP/6-31G(2df,p) Gibbs free energies (where the SMD single-point calculations were carried out at the M05-2X/6-31G(d) level of theory, as recommended by Marenich, Cramer and Truhlar).[10] For the sake of brevity, this level of theory is denoted SMD(pyridine)-G4(MP2). All calculations were carried out using the Gaussian 16 rev. A.03 program suite.[12]

The G4(MP2) composite protocol is an efficient composite procedure for approximating the CCSD(T) energy (coupled cluster with singles, doubles, and quasiperturbative triple excitations) in conjunction with a large triple-z-quality basis set.[13,14] This protocol is widely used for the calculation of thermochemical and kinetic properties (for a recent review of the Gaussian-n methods see Ref. 13). G4(MP2) theory has been found to produce thermochemical properties (such as reaction energies, bond dissociation energies, and enthalpies of formation) with a mean absolute deviation of 4.4 kJ mol^{-1} from the 454 experimental energies of the G3/05 test set.[12,14] It has also been found that G4(MP2) shows a similarly good performance for reaction barrier heights.[15-18]

SMD(pyridine)-B3LYP-D3BJ/6-31G(2df,p) optimized geometries used in the G4(MP2) calculations.

Pyridine

N	0.000000	0.000000	1.415402
C	0.000000	1.145732	0.721482
C	-0.000000	-1.145732	0.721482
C	0.000000	1.197786	-0.671242
H	0.000000	2.062665	1.305338
C	-0.000000	-1.197786	-0.671242
H	-0.000000	-2.062665	1.305338
C	0.000000	0.000000	-1.382428
H	0.000000	2.155522	-1.179639
H	-0.000000	-2.155522	-1.179639
H	0.000000	0.000000	-2.467530

MeS_2Me

S	0.443157	0.932213	-0.508883
S	-0.443157	-0.932213	-0.508883
C	0.443157	-1.818437	0.819962
H	0.264179	-1.344988	1.787297
H	0.031393	-2.833100	0.832260
H	1.512232	-1.860257	0.602804
C	-0.443157	1.818437	0.819962
H	-0.264179	1.344988	1.787297
H	-0.031393	2.833100	0.832260
H	-1.512232	1.860257	0.602804

MeS_3Me

S	0.000000	1.675184	-0.161793
S	0.000000	0.000000	1.060474
S	-0.000000	-1.675184	-0.161793
C	-1.757622	-1.842187	-0.620313
H	-1.824114	-2.739203	-1.244378
H	-2.375983	-1.966396	0.270551
H	-2.079499	-0.974237	-1.199407
C	1.757622	1.842187	-0.620313
H	2.079499	0.974237	-1.199407
H	1.824114	2.739203	-1.244378
H	2.375983	1.966396	0.270551

MeS_2^-

S	-0.444649	-0.702664	-0.000001
S	1.415386	0.273400	-0.000000
C	-1.641937	0.673784	-0.000001
H	-1.516262	1.293590	-0.893431
H	-2.648266	0.237819	0.000467
H	-1.515635	1.294106	0.892985

MeS^-

S	0.000000	0.000000	0.713675

C	0.000000	0.000000	-1.134863
H	0.000000	1.020811	-1.536540
H	0.884048	-0.510405	-1.536540
H	-0.884048	-0.510405	-1.536540

Pyridine-S-Me$^+$

N	0.247859	-0.164350	0.000000
C	0.249439	0.495598	1.182520
C	0.249439	0.495598	-1.182520
C	0.249439	1.875580	1.204012
H	0.251269	-0.115932	2.074292
C	0.249439	1.875580	-1.204012
H	0.251269	-0.115932	-2.074292
C	0.249797	2.577523	-0.000000
H	0.251359	2.384696	2.159098
H	0.251359	2.384696	-2.159098
H	0.252830	3.661370	-0.000000
S	0.265352	-1.921576	0.000000
C	-1.523617	-2.216287	0.000000
H	-1.980554	-1.808995	-0.903500
H	-1.621231	-3.306791	0.000000
H	-1.980554	-1.808995	0.903500

Pyridine-S_2-Me$^+$

N	0.566683	-0.239332	-0.428107
C	1.251471	-1.179153	0.262923
C	2.515864	-0.897789	0.738414
H	0.755849	-2.128984	0.412669
C	2.350055	1.302561	-0.230040
C	3.074653	0.355707	0.491409
H	3.050302	-1.660063	1.290769
H	2.751257	2.285199	-0.442270
H	4.067338	0.591546	0.857288
S	-1.069357	-0.618538	-1.038958
S	-2.255464	-0.389235	0.585421
C	-2.585950	1.404780	0.644662
H	-2.993534	1.745437	-0.308002
H	-3.332365	1.532098	1.435393
H	-1.678175	1.949123	0.908772
C	1.085223	0.983752	-0.683402
H	0.461805	1.666185	-1.245076

Pyridine•••MeS_2Me reactant complex

S	3.070446	0.782481	0.030150
S	2.366918	-1.142166	0.281755
C	1.663999	1.820081	0.562334
H	1.423233	1.627250	1.609590
H	2.001690	2.856100	0.451292
H	0.793548	1.649026	-0.073253

N	-1.501151	0.509524	-0.534499
C	-2.688687	0.804936	-1.078124
C	-1.494884	-0.236328	0.577860
C	-3.902790	0.375660	-0.544849
H	-2.668107	1.410671	-1.980552
C	-2.654338	-0.712489	1.186069
H	-0.518742	-0.464014	0.997716
C	-3.884755	-0.399537	0.612327
H	-4.834904	0.645218	-1.028945
H	-2.587987	-1.312942	2.086530
H	-4.809319	-0.752294	1.057347
C	1.448082	-1.458710	-1.265146
H	0.638355	-0.737508	-1.386565
H	2.125694	-1.430141	-2.120812
H	1.027017	-2.464759	-1.164154

Pyridine + MeS_2Me transition structure

S	1.422885	1.077147	0.008944
S	1.566052	-1.408347	-0.009744
C	0.519726	2.677197	-0.009500
H	-0.097003	2.829366	0.879021
H	1.343473	3.405306	-0.004084
H	-0.074981	2.820193	-0.914472
N	-0.727982	0.354689	0.001800
C	-1.327846	0.083968	-1.158185
C	-1.327972	0.081780	1.161220
C	-2.595271	-0.487048	-1.200622
H	-0.775219	0.325897	-2.059600
C	-2.595559	-0.488773	1.202739
H	-0.774898	0.321970	2.062810
C	-3.238330	-0.778457	0.000769
H	-3.059127	-0.697398	-2.157050
H	-3.059578	-0.700482	2.158817
H	-4.225854	-1.226813	0.000159
C	3.362222	-1.223680	0.005332
H	3.582973	-0.139022	-0.000336
H	3.807800	-1.650240	0.908355
H	3.823463	-1.662315	-0.883957

Pyridine-S-Me^{+}•••MeS^{-} product complex

S	0.822110	1.293381	0.042379
S	1.919276	-1.547780	-0.065364
C	-0.023424	2.917276	0.146496
H	-0.611498	3.002761	1.063455
H	0.792924	3.646908	0.173056
H	-0.644113	3.107400	-0.732381
N	-0.671214	0.350675	0.019343
C	-1.314154	0.159565	-1.151001
C	-1.118821	-0.193789	1.169299

C	-2.465833	-0.602226	-1.196525
H	-0.870510	0.620592	-2.022312
C	-2.267430	-0.962442	1.173537
H	-0.528598	0.002839	2.052646
C	-2.950994	-1.173751	-0.021783
H	-2.962962	-0.746468	-2.147228
H	-2.609278	-1.389720	2.107476
H	-3.849528	-1.779653	-0.038868
C	3.545639	-0.699364	-0.102403
H	3.417340	0.392215	-0.029197
H	4.184266	-1.009532	0.732792
H	4.088383	-0.903303	-1.032793

Pyridine•••MeS_3Me reactant complex

S	-2.148019	-1.023922	0.757125
S	-2.426102	0.702817	-0.356265
S	-0.557554	1.514255	-0.733750
C	-0.195151	2.418751	0.806172
H	0.771992	2.906339	0.652682
H	-0.960709	3.173556	0.996034
H	-0.120733	1.722065	1.642853
C	-1.710074	-2.257406	-0.511798
H	-0.780030	-1.974584	-1.007709
H	-1.564023	-3.202303	0.021530
H	-2.521868	-2.365974	-1.233585
N	1.845799	-1.570151	-0.694293
C	2.559236	-0.596634	-1.273085
C	1.623865	-1.476210	0.622876
C	3.078182	0.491847	-0.574650
H	2.722500	-0.689962	-2.343773
C	2.102104	-0.429708	1.408015
H	1.035693	-2.272799	1.070120
C	2.847114	0.574752	0.796055
H	3.647910	1.251605	-1.098194
H	1.888948	-0.406732	2.470877
H	3.234880	1.407089	1.373940

Pyridine + MeS_3Me transition structure

S	-0.694568	1.357445	-0.190911
S	-1.160307	-1.196598	-0.439727
C	0.290427	2.875406	0.084101
H	1.021753	3.054445	-0.707618
H	-0.464082	3.674530	0.055480
H	0.779330	2.893106	1.061197
N	1.434960	0.410811	0.008575
C	1.872851	0.030695	1.209664
C	2.089357	0.048466	-1.095703
C	3.017249	-0.746959	1.355214
H	1.287276	0.353562	2.064543

C	3.243324	-0.727007	-1.036890
H	1.671607	0.383706	-2.039409
C	3.713150	-1.132382	0.210825
H	3.347330	-1.041644	2.344461
H	3.754570	-1.003483	-1.951617
H	4.606893	-1.741673	0.290485
S	-3.062189	-0.558939	-0.486404
C	-3.598373	-0.575543	1.259585
H	-4.597012	-0.128076	1.289852
H	-3.641446	-1.603787	1.625010
H	-2.905810	0.017052	1.859478

Pyridine-S_2-Me^+•••MeS^- product complex

S	-0.213575	1.240213	-0.182144
S	-1.482008	-1.522022	-0.560120
C	0.549245	2.879457	0.108744
H	1.287393	3.123947	-0.658939
H	-0.285564	3.584927	0.039228
H	0.990269	2.943160	1.106569
N	1.310386	0.337935	0.001808
C	1.592968	-0.230531	1.191776
C	2.121823	0.183069	-1.064511
C	2.737767	-0.988663	1.347765
H	0.876272	-0.062231	1.983227
C	3.277330	-0.567400	-0.958358
H	1.805815	0.663603	-1.980271
C	3.591864	-1.164395	0.260967
H	2.944374	-1.436318	2.311499
H	3.911278	-0.682445	-1.828265
H	4.490157	-1.762445	0.361700
S	-3.119029	-0.242023	-0.477589
C	-3.461337	-0.124710	1.310024
H	-4.300279	0.568560	1.441889
H	-3.733015	-1.105092	1.712435
H	-2.583563	0.259143	1.837462

MeS^- + MeS_2Me transition structure

S	-0.016311	-0.139298	0.271641
S	-2.352476	-0.823322	0.064715
C	-3.126202	0.824707	0.251450
H	-2.878003	1.474584	-0.594533
H	-4.215464	0.716214	0.293450
H	-2.788170	1.306689	1.174549
C	0.011830	0.650130	-1.390243
H	0.069356	1.736232	-1.292836
H	0.875082	0.292339	-1.954487
H	-0.902461	0.370310	-1.915114
S	2.346545	0.525882	0.618581
C	3.150314	-0.771815	-0.392255

H	2.828114	-1.767951	-0.072035
H	2.914078	-0.652733	-1.455350
H	4.237685	-0.706003	-0.276324

MeS^- + MeS_3Me transition structure

S	-0.572294	0.069556	-0.290518
S	-3.010986	1.011337	0.067841
C	-3.857456	-0.589953	-0.217072
H	-3.643612	-1.302311	0.587910
H	-4.942654	-0.444115	-0.258742
H	-3.535839	-1.036664	-1.164575
C	-0.705432	-0.938111	1.236177
H	-0.880557	-1.987986	0.990330
H	0.220339	-0.843532	1.808097
H	-1.536604	-0.543829	1.820667
S	1.474777	-0.856930	-0.855233
S	2.887450	-0.184559	0.506580
C	3.222607	1.520160	-0.052591
H	2.307966	2.116046	-0.012492
H	3.961065	1.941276	0.638097
H	3.628424	1.518092	-1.067111

MeS_2^- + MeS_2Me transition structure

S	0.573713	0.072529	0.294794
S	3.013488	1.009717	-0.057921
C	3.858486	-0.597399	0.196175
H	3.654126	-1.290247	-0.627971
H	4.942977	-0.451607	0.253155
H	3.526903	-1.066683	1.129192
C	0.703290	-0.929840	-1.235545
H	0.886915	-1.979342	-0.994202
H	-0.226960	-0.839378	-1.800775
H	1.527826	-0.528421	-1.824688
S	-1.475796	-0.852394	0.860816
S	-2.889675	-0.190797	-0.504850
C	-3.222387	1.519602	0.038056
H	-2.307982	2.115098	-0.012037
H	-3.963479	1.933244	-0.654316
H	-3.624335	1.528287	1.054093

MeS_2^- + MeS_3Me transition structure

S	0.008462	-0.032198	-0.256737
S	2.151228	-1.251836	-0.358228
C	-0.053716	-0.041760	1.574820
H	0.151605	0.956696	1.966578
H	-1.045410	-0.367746	1.895645
H	0.697333	-0.743078	1.938784
S	-2.099742	1.238237	-0.338197
S	-3.583110	0.058713	0.497529

C	-3.962578	-1.143421	-0.822927
H	-3.076528	-1.736213	-1.061950
H	-4.748266	-1.802811	-0.437925
H	-4.322322	-0.628630	-1.717521
S	3.582284	-0.025744	0.505919
C	3.918199	1.208872	-0.795692
H	3.008880	1.766401	-1.032583
H	4.672881	1.896272	-0.397757
H	4.304454	0.722222	-1.695062

Repair of polymers with varying sulfur rank (1, 1.5, 2, 2.5, 3)

Synthesis of polymer with average sulfur rank ranging from 1 to 3

The ratio of sulfur, canola oil and dicyclopentadiene (DCPD) was varied to synthesize 6 different polymers which ranged in sulfur rank from 1 to 3. The ratio of canola oil to DCPD was maintained at 7 to 3, which matched that of the original polymer used in this study. The ratio of sulfur was altered to adjust the number of sulfur atoms per alkene in the reaction (the average sulfur rank). A total batch size of 10 g was used for each polymer, which included the combined mass of the sulfur, canola oil and DCPD. The calculated composition of the polymers can be seen in the table below.

Mass of Sulfur per 10 g Batch (g)	Mass of DCPD per 10 g Batch (g)	Mass of Canola Oil per 10 g Batch (g)	mmol sulfur atoms per 10 g batch	mmol DCPD per 10 g batch	mmol canola oil per 10 g batch	mmol total alkenes per 10 g batch*	Theoretical average sulfur rank*
2	2.4	5.6	63	18.2	6.3	58	1
2.71	2.19	5.1	85	16.6	5.8	53	1.5
3.31	2.01	4.68	103	15.2	5.3	48	2
3.83	1.85	4.32	120	14.0	4.9	45	2.5
4.26	1.72	4.02	133	13.0	4.6	42	3

* Calculation assumes 2 alkenes for each molecule of DCPD and an average of 3.4 alkenes per molecule of canola oil. Sulfur rank is reported to nearest 0.5 unit.

All polymers were synthesised with the same method as the original polymer used in this study. The sulfur was added to a 25 mL glass vial with a magnetic stirrer. The sulfur was then heated at 170 °C for 2.5 minutes on an aluminium hot block to melt the sulfur and initiate ring-opening polymerisation. Constant stirring at 400 rpm was maintained during heating. Separately, the canola oil and DCPD were added to a second 25 mL glass vial. This vial was heated for 30 seconds and added to the sulfur while heating and stirring was maintained. The reaction was heated at 170 °C for 13 minutes before being poured directly into a preheated silicone mold. The mold and polymer were then added to an oven preheated to 130 °C and cured for 24 hours. All compositions of polymer had the same dark appearance but the lower sulfur rank polymers were noticeably more flexible and would bend under their own weight when a dogbone shaped piece was held at the clamping section of one end, as can be seen on the following page.

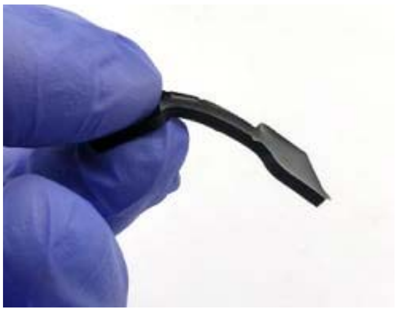
Sulfur Rank 1

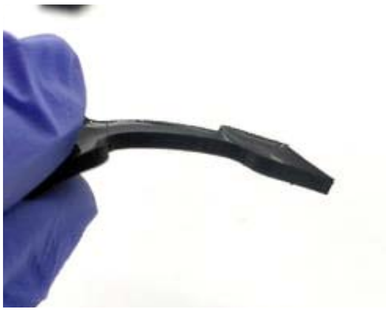
Sulfur Rank 1.5

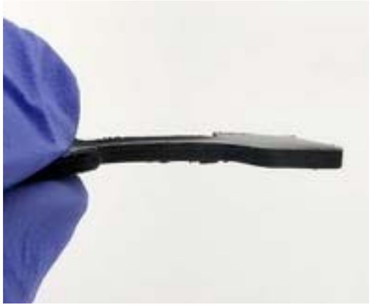
Sulfur Rank 2

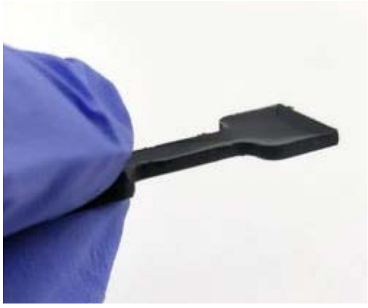
Sulfur Rank 2.5

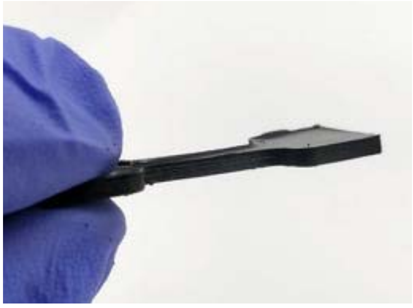
Sulfur Rank 3

STA of polymers with sulfur rank ranging from 1 to 3

Simultaneous thermal analysis (STA) was performed on all polymers using a Perkin Elmer STA8000 to identify if there was any unreacted sulfur and to observe any mass losses that correlated with polysulfide decomposition (first mass loss). Approximately 15 mg of each polymer was ground into a powder and added to the STA. The temperature was scanned from 50 °C to 600 °C at a rate of 20 °C/ min under nitrogen. After the temperature reached 600 °C, oxygen was added to burn off any remaining products.

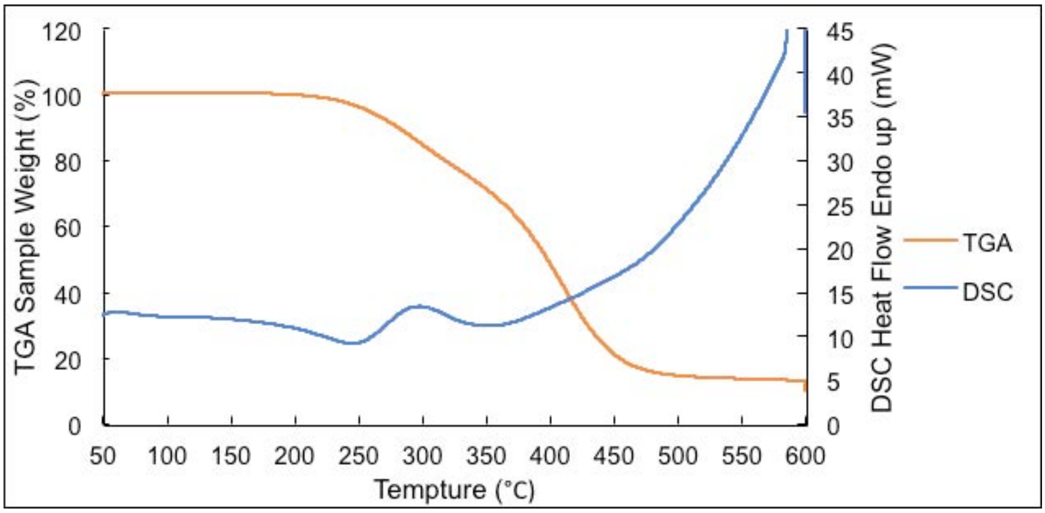

STA of polymer with a sulfur rank of 1.

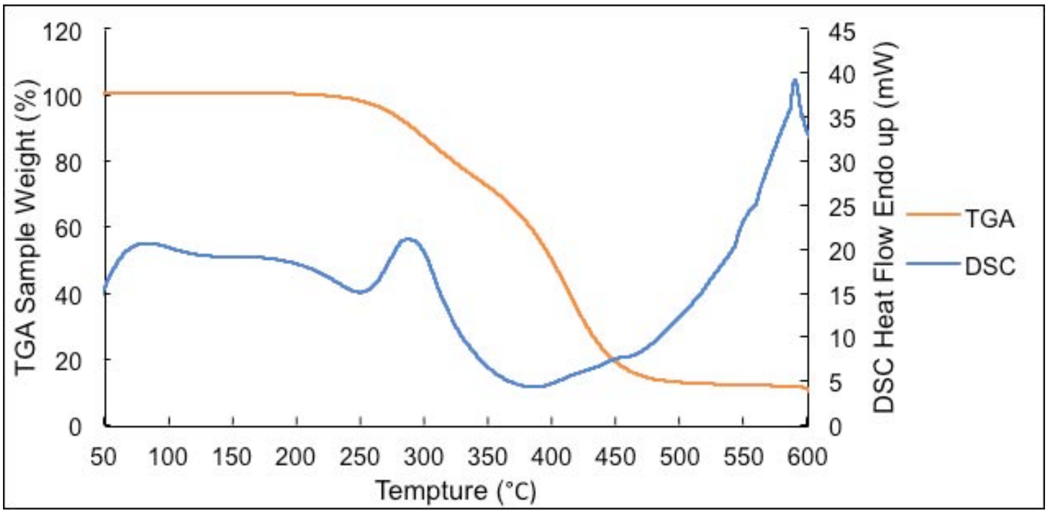

STA of polymer with a sulfur rank of 1.5.

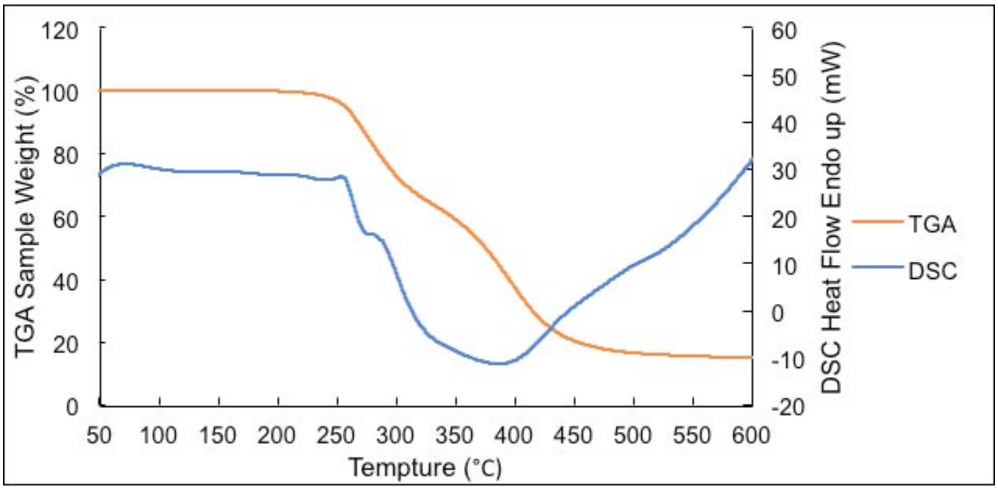

STA of polymer with a sulfur rank of 2.

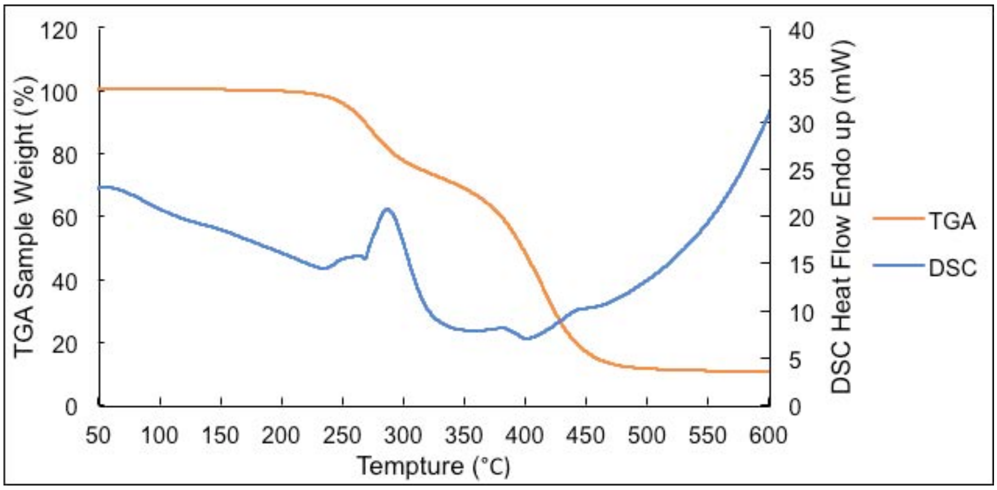

STA of polymer with a sulfur rank of 2.5

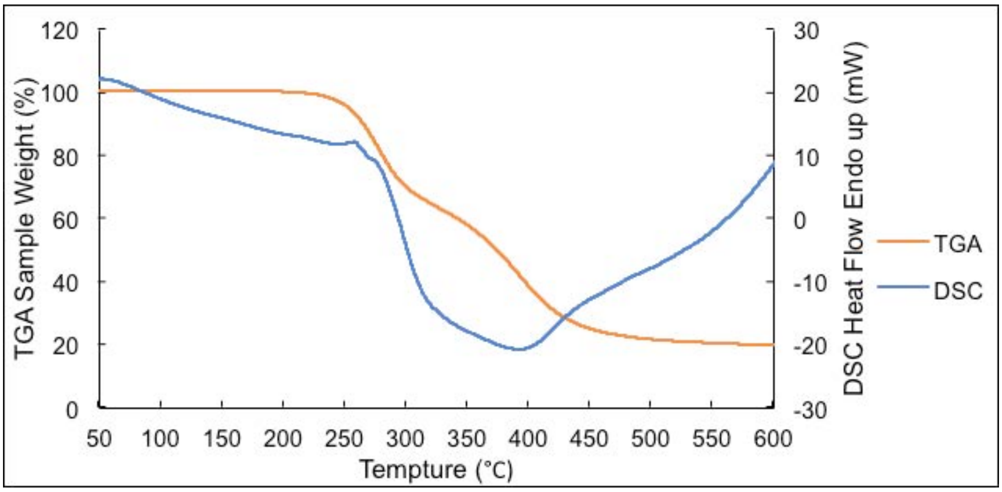

STA of polymer with a sulfur rank of 3

Repair of polymers with different sulfur rank

Six dogbone shaped polymer pieces for each sulfur rank from 1 to 3 were cut down the centre of the gauge section with a scalpel. Three of these pieces were replaced into the mold and repaired with 10 μL of pyridine and three were repaired with 10 μL of tributylphosphine. Another three samples were left undamaged and returned to the mold as controls. The polymer pieces were left for 24 hours to react with no compression before being removed from the mold and tested for tensile strength using dynamic mechanical analysis. The pieces were clamped at either end and a tensile force was applied which ramped at 0.2 N/min until failure of the polymer. No temperature control was used. The stress at failure indicates the strength of the repaired polymer. The stress at failure was compared to the undamaged control samples to indicate the extent of repair of the polymer (reported as % of recovered tensile strength compared to the uncut dogbone of the same sulfur rank):

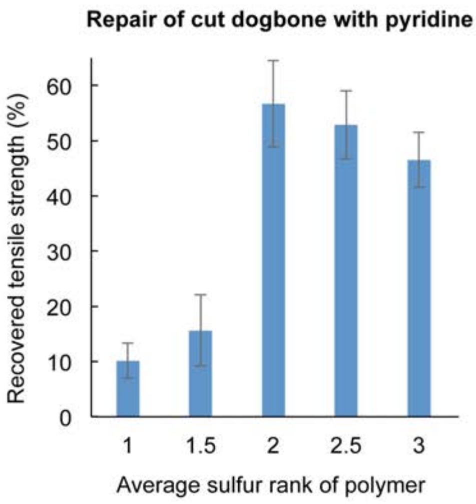

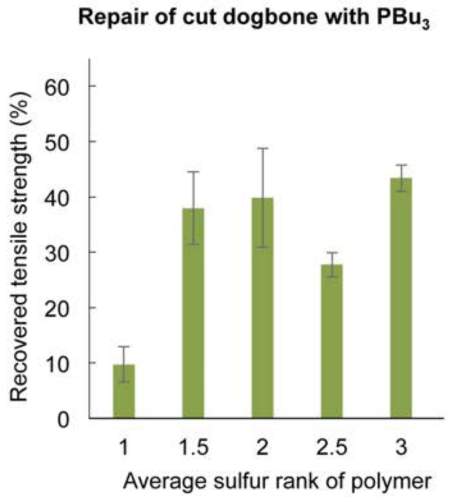

Repair of polymers using triethylamine, 2,6-lutidine, and ethyl nicotinate

Polymers with the standard composition of 50 % sulfur, 35 % canola oil and 15 % DCPD (sulfur rank of approximately 4) were cut down the centre of the gauge section, returned to the mold and repaired using 10 µL of triethylamine, 2,6-lutidine or ethyl nicotinate. The experiments were carried out in triplicate, along with three uncut control samples. The samples were left for 24 hours to react in the mold (with out compression) before tensile testing by DMA. The same method was used as previous tensile testing with a force ramp rate of 0.2 N/min using a film tension clamp. The stress at failure (MPa) is reported below for each sample:

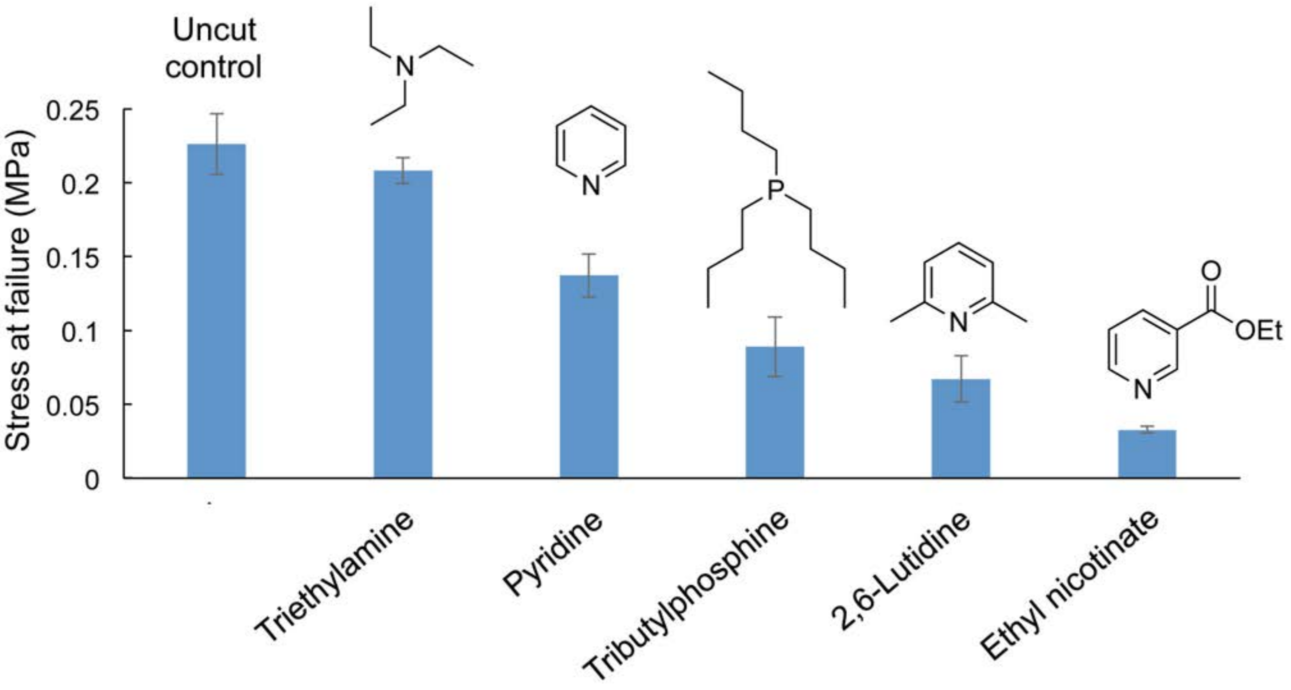

Disulfide crossover experiments using triethylamine, 2,6-lutidine, and ethyl nicotinate catalysts

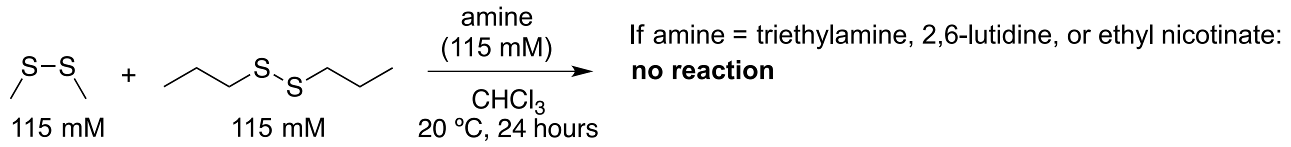

Dimethyl disulfide (10.2 µL, 0.115 mmol) was added to a 1.5 mL GC-MS vial. Chloroform was then added. The volume of chloroform was such that the final reaction volume was 1 mL. Di-*n*-propyl disulfide (18 µL, 0.115 mmol) was then added. Finally, either triethylamine, 2,6-lutidine or ethyl nicotinate was added to the vial so that the final concentration of the amine was 115 mM. The vial was then capped and the GC-MS of the reaction mixture was analyzed several times over 24 hours. No reaction was detected after 24 hours. GC traces are shown below.

GC-MS method: The GC was initially heated to 30 degrees where it was held for 3 minutes after sample injection. The column was then heated at 20 degrees per minute to 250 degrees. This gave a total run time of 14 minutes. The detector was delayed until 2.75 minutes to prevent the solvent from damaging the detector. Using this method, triethylamine was eluted at 3.123 minutes, dimethyl disulfide at 4.164 minutes, 2,6-lutidine at 6.121 minutes, dipropyl disulfide at 8.41 minutes, and ethyl nicotinate at 9.245 minutes. The samples were placed in an autosampler and tested regularly over 24 hours.

No crossover with triethylamine (115 mM in $CHCl_3$, 24 h):

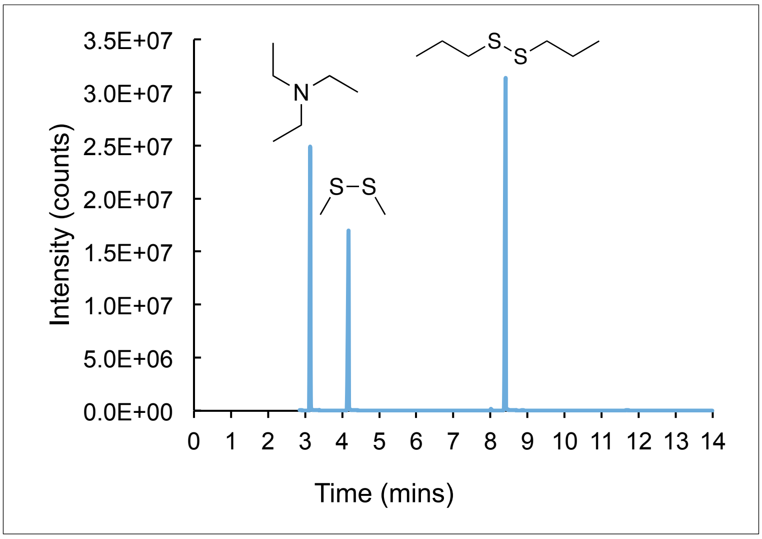

No crossover with 2,6-lutidiene (115 mM in $CHCl_3$, 24 h):

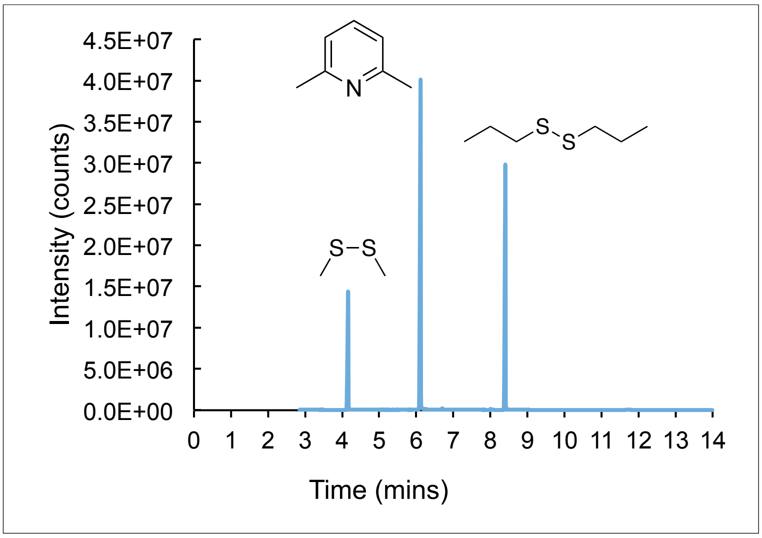

No crossover with ethyl nicotinate (115 mM in $CHCl_3$, 24 h):

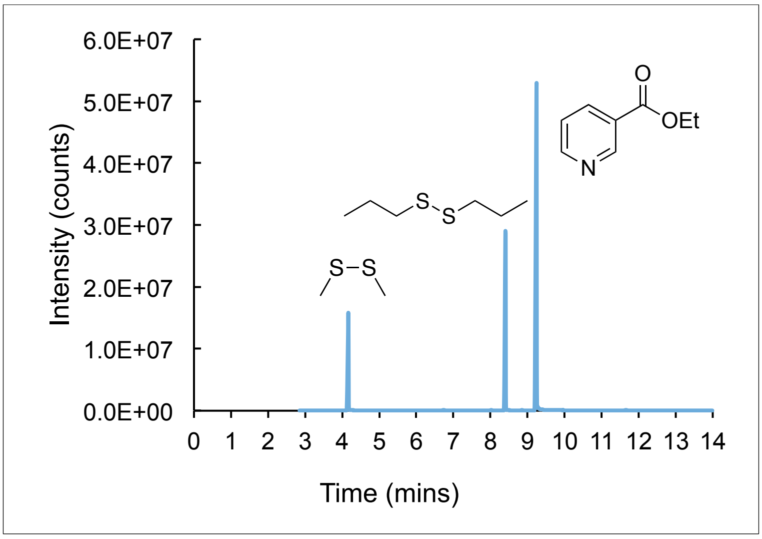

Trisulfide crossover experiments using triethylamine, 2,6-lutidine, and ethyl nicotinate catalysts

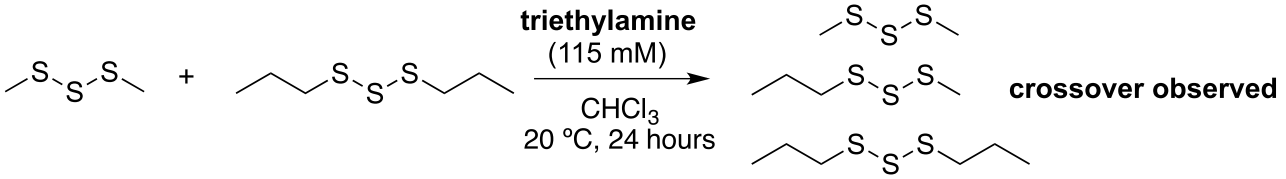

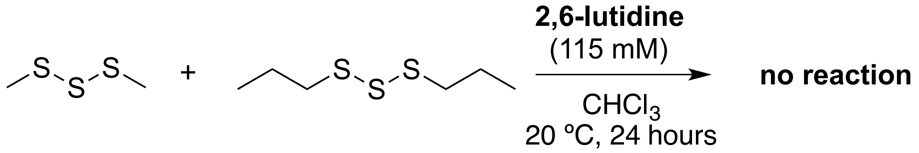

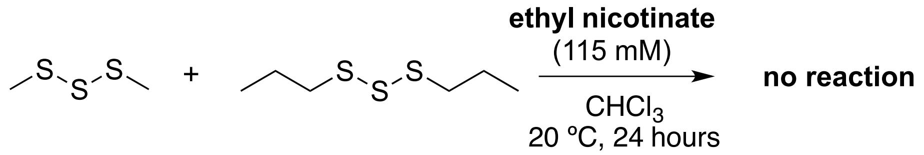

Dimethyl trisulfide (12.1 µL, 0.115 mmol) was added to a 1.5 mL GC-MS vial. Chloroform was then added. The volume of chloroform was such that the final reaction volume was 1 mL. Di-*n*-propyl triisulfide (17.9 µL, 0.115 mmol) was then added. Finally, either triethylamine, 2,6-lutidine or ethyl nicotinate was added to the vial so that the final concentration of the amine was 115 mM. Separate vials were made for each catalyst. The vial was then capped and the GC-MS of the reaction mixture was analyzed several times over 24 hours. The triethylamine experiment was also analyzed at 48 hours. The GC traces are shown on the next page.

GC-MS method: The GC was initially heated to 30 degrees where it was held for 3 minutes after sample injection. The column was then heated at 20 degrees per minute to 250 degrees. This gave a total run time of 14 minutes. The detector was delayed until 2.75 minutes to prevent the solvent from damaging the detector. Using this method, triethylamine was eluted at 3.123 minutes, 2,6-lutidine at 6.121 minutes, dimethyl trisulfide at 7.145 minutes, ethyl nicotinate at 9.245 minutes, and di-*n*-propyl trisulfide at 10.115 minutes. The samples were placed in an autosampler and tested regularly over 24 hours.

Triethylamine catalyzed crossover of trisulfides:

No crossover at **5 min** (triethylamine, 115 mM in $CHCl_3$):

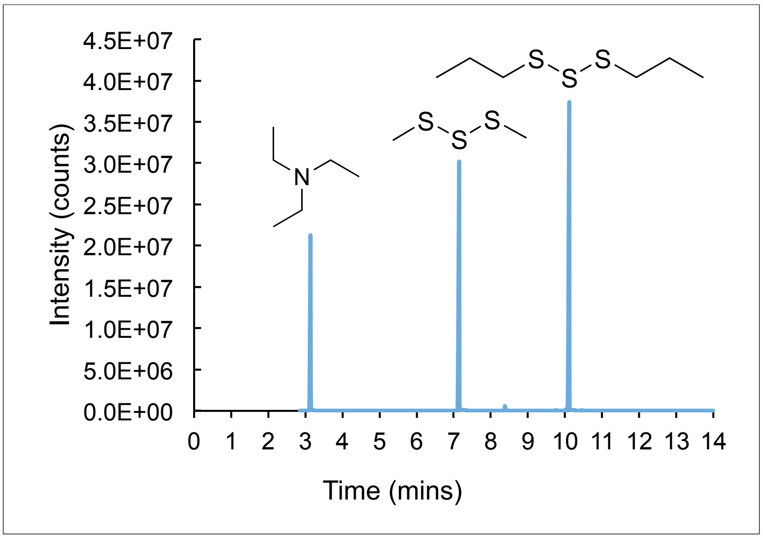

Crossover observed with triethylamine (115 mM in $CHCl_3$, **24 h**):

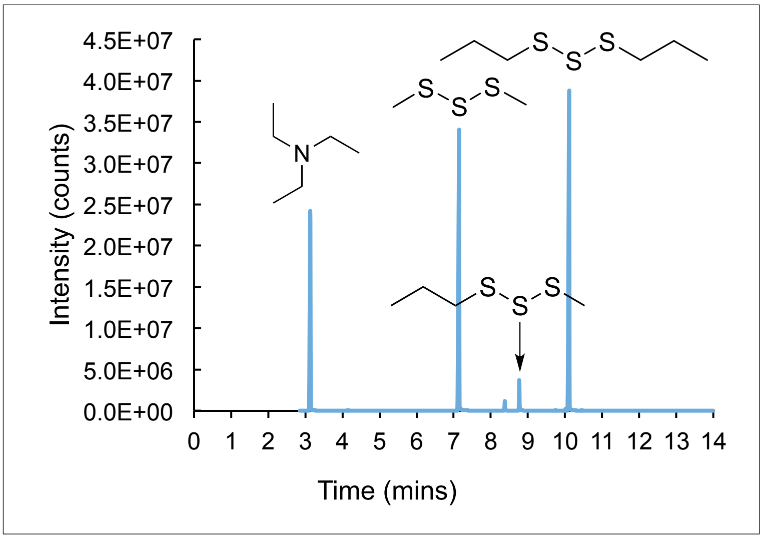

Crossover observed with triethylamine (115 mM in $CHCl_3$, **48 h**):

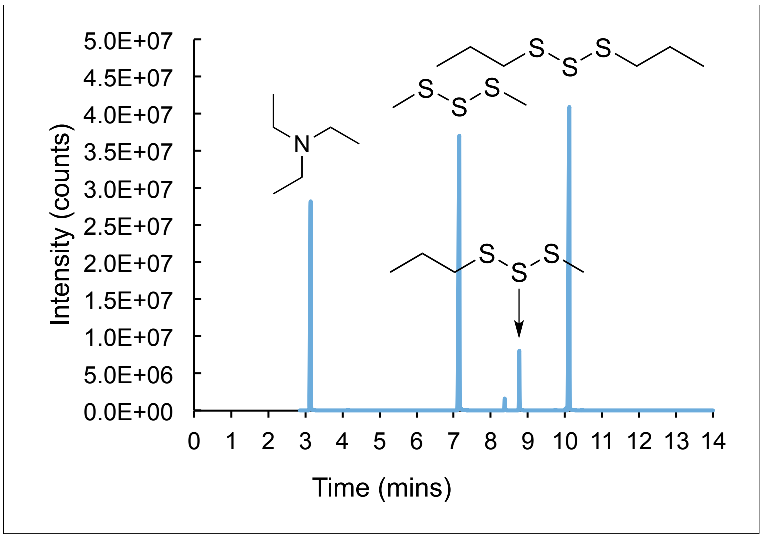

No crossover with 2,6-lutidiene (115 mM in $CHCl_3$, 24 h):

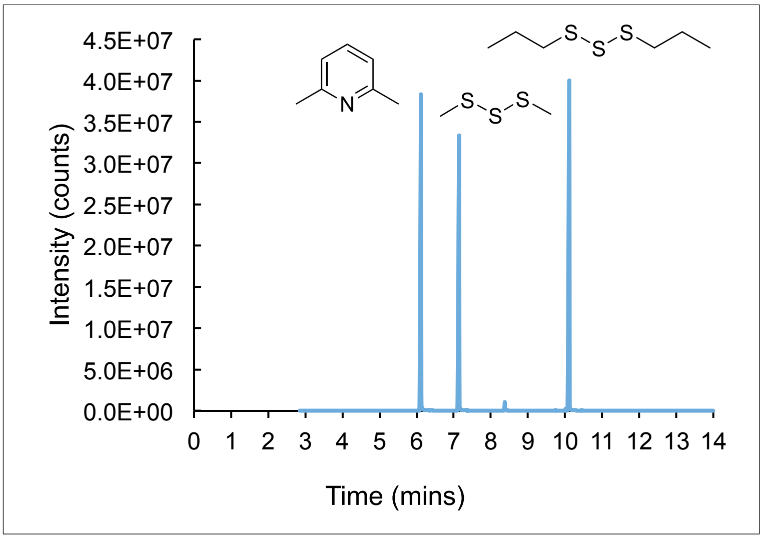

No crossover with ethyl nicotinate (115 mM in $CHCl_3$, 24 h):

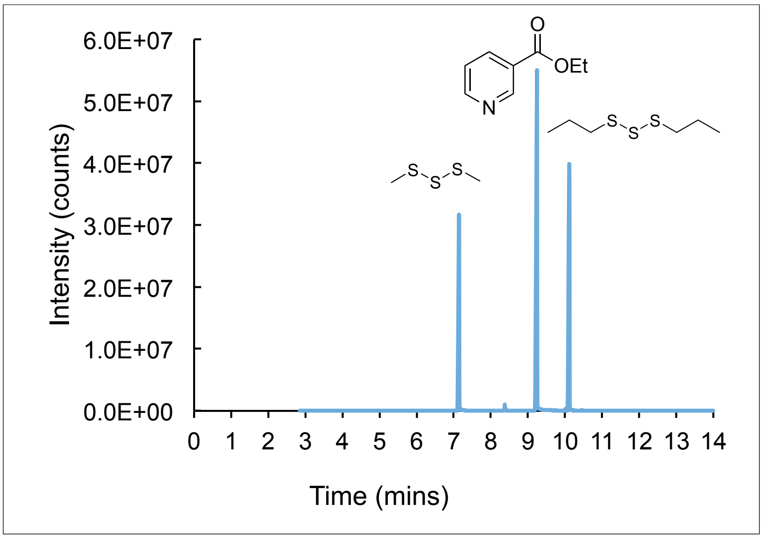

Synthesis and attempted repair of a polymer with a higher glass transition temperature (50 wt% sulfur, 35 wt% DCPD, 15 wt% canola oil)

CAUTION!

At this feed ratio of DCPD a lower temperature and shorter reaction time is required in the preparation of the pre-polymer. If longer heating and higher temperatures were applied, a highly exothermic runaway reaction resulted in which the pre-polymer bubbled out of the reaction vial. We recommend keeping this polymerization at a scale of less than 10 g. Never cap the vial during the reaction so no pressure build-up occurs.

Image of product after runaway reaction after heating monomers in a vial on a hotblock for 13 minutes at 170 ºC:

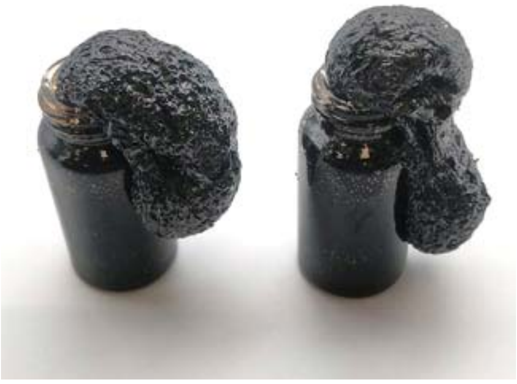

Modified protocol to accommodate higher DCPD feed:

Sulfur (5.0 g, 156 mmol sulfur atoms) was added to a 25 mL vial. DCPD (3.5 g, 26.5 mmol) and canola oil (1.5 g, 1.70 mmol) were added to a second 25 mL vial. The vial containing sulfur was added to a hot block and heated to 160 ºC with stirring for 2.5 minutes. The vial containing DCPD and canola oil was pre-heated to 160 ºC and then poured into the vial containing sulfur. The reaction mixture was stirred at 160 ºC for 6 minutes to form the liquid pre-polymer and then the liquid was immediately removed from the hotblock and poured into the silicone dogbone mold. The mold was placed in an oven pre-heated to 130 ºC and the polymer was cured for 24 hours. The resulting polymer was black and very brittle. The average sulfur rank for this polymer (based on the molar ratio of sulfur atoms to total alkenes) is 2.7. Images of the polymer dogbones are shown below.

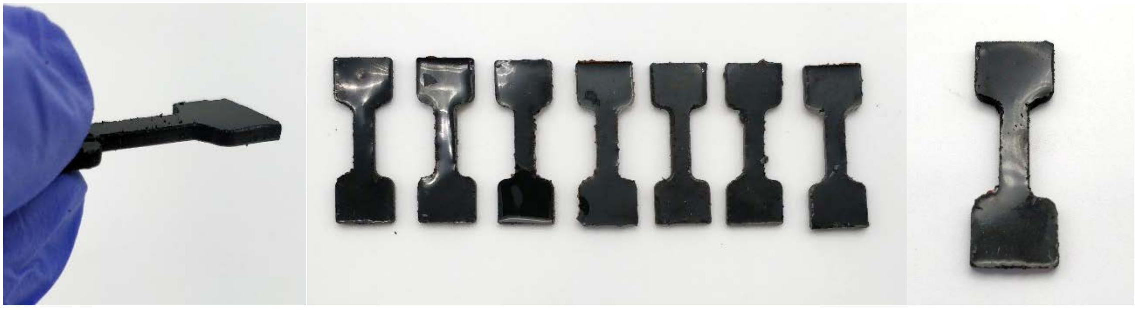

Glass transition temperature (50 wt% sulfur, 35 wt% DCPD, 15 wt% canola oil): Approximately 10 mg of polymer was ground and placed into a DSC tray. A Perkin Elmer DSC 8000 was used to perform a temperature scan of the polymer from -50 °C to 150 °C at a ramp rate of 10 °C/min. The temperature was scanned over this range three times to find the glass transition. The region of the scan between 20 ºC and 100 ºC is shown below, which contains the glass transition temperature (60 ºC).

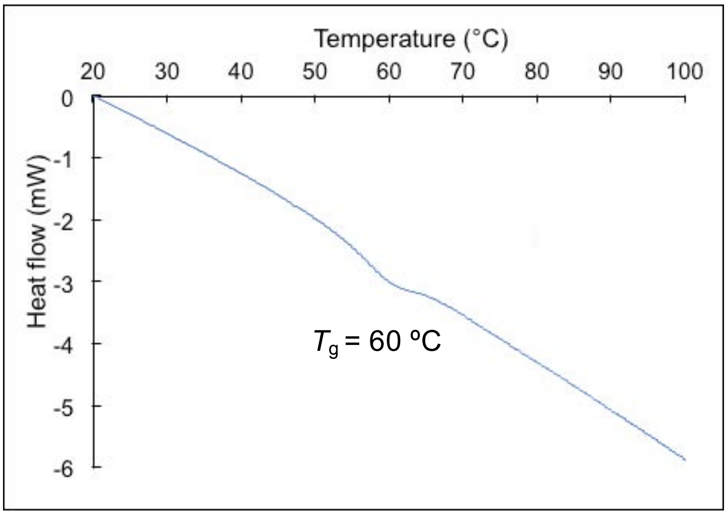

Tensile modulus (50 wt% sulfur, 35 wt% DCPD, 15 wt% canola oil): The polymer was tested for tensile strength using a TA Q800 dynamic mechanical analyser. A polymer dogbone was clamped at both ends and a tensile force was applied to the polymer. The force ramped at a rate of 0.2 N/min until failure. The polymer was compared to the original polymer with a composition of 50 % sulfur, 35 % canola oil and 15 % DCPD which had a T_g = -9 ºC. The tensile modulus was calculated using the slope of the linear section of the stress-strain curve shown below. The tensile modulus of the higher T_g polymer was 1092 Mpa and the tensile modulus of the original polymer had a tensile modulus was 2.11 Mpa.

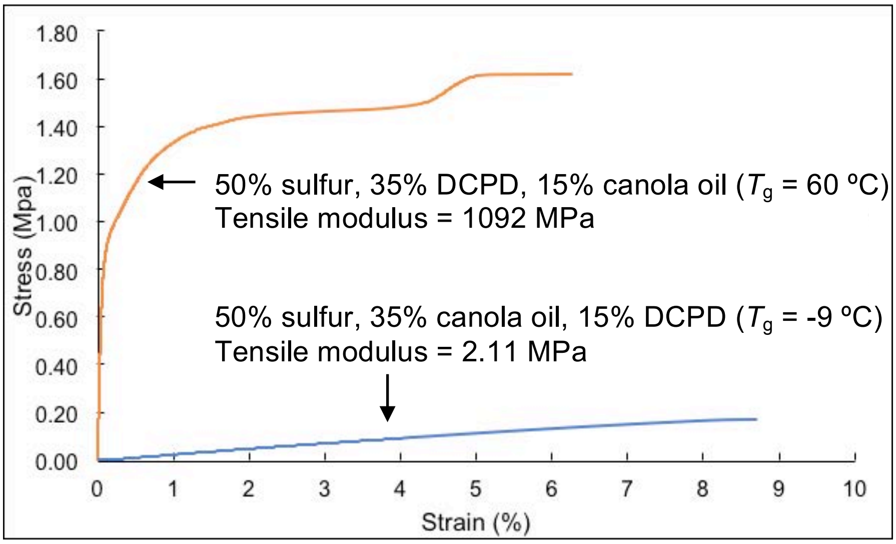

Attempted repair of polymer dogbone made from 50 wt% sulfur, 35 wt% DCPD, 15 wt% canola oil (T_g = 60 ºC).

Six dogbone shaped polymer pieces were cut down the centre of the gauge section with a scalpel. Three of these pieces were replaced into the mold and 10 µL of pyridine was applied to the cut section of three of the samples and 10 µL of tributylphosphine was applied to the cut section of the other three samples. The polymer pieces were left for 24 hours to react with no compression. After this time, the polymer pieces were removed from the mold. No repair occurred in any of the samples. This experiment was repeated and again repair was not observed for any sample.

Mechanistic proposal for polymer repair with tributylphosphine

Average sulfur rank = 3 (disulfides, trisulfides, and tetrasulfides shown in this polymer network)

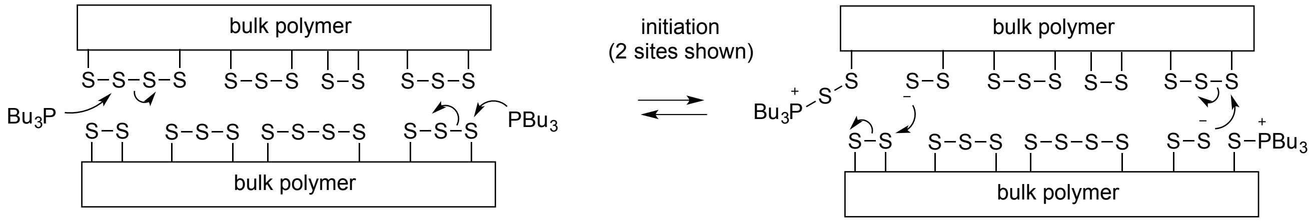

Based on crossover experiments, PBu_3 can attack disulfides and polysulfides of higher sulfur rank. Here, PBu_3 is shown attacking tetrasulfides and trisulfides on the polymer surface.

Persulfide anion can attack other S-S bonds. Computations show this reaction has a low-lying barrier. S-S group attacked can be disulfide or higher sulfur rank. Attack can be interpolymer or intrapolymer.

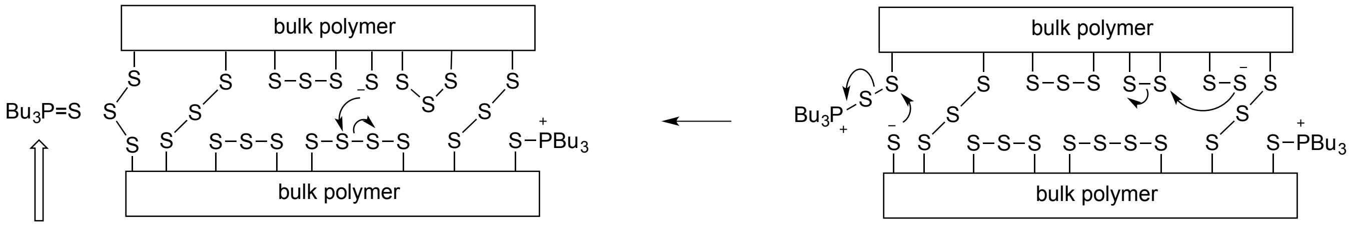

PBu_3 is converted into tributylphosphine sulfide. This substance was recovered from the polymer after the reaction with PBu_3. This is a termination event in the polymer repair. It should also be noted that each phosphorous atom will remove one sulfur atom from the polymer

Note: the S-S metathesis requires mobility of these functional groups for reaction to occur. This may require the polymer species at the interface to be above their glass transition temperature. The liquid tributylphosphine at the interface may also facilitate this mass transfer through solvation of the reactive groups. However, control experiments indicate that neither inert solvents (such as chloroform, toluene, THF, etc) nor physical contact alone are sufficient for the polymers pieces to bond together. A reagent such as PBu_3 that breaks S-S bonds is required to join the polymer interfaces.

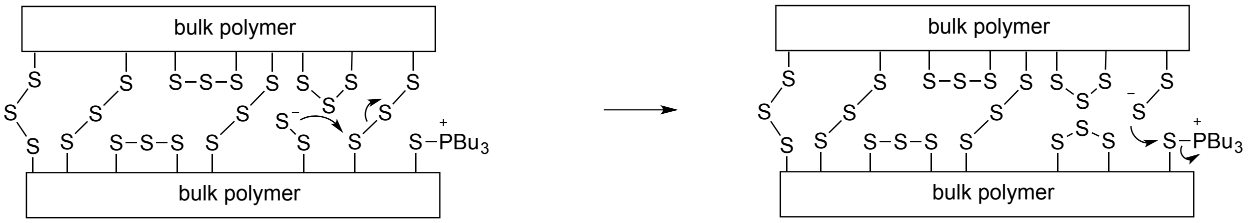

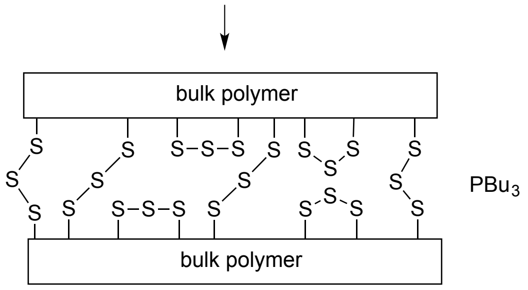

The regenerated PBu_3 catalyst can attack another polysulfide (re-initiating S-S metathesis). The reaction is complete when all PBu_3 is converted to $Bu_3P{=}S$, which remains in the polymer unless extracted with solvent. The phosphine can also be converted to phosphine oxide if air or water are present.

Note that excess phosphine will break so many S-S bonds that the polymer network will break down and dissolve. With catalytic quantities, S-S metathesis results in adhesion between the two polymer interfaces

Mechanistic proposal for polymer repair with pyridine

(Triethylamine is expected to catalyze the reaction by the same mechanism)

Average sulfur rank = 3 (disulfides, trisulfides, and tetrasulfides shown in this polymer network)

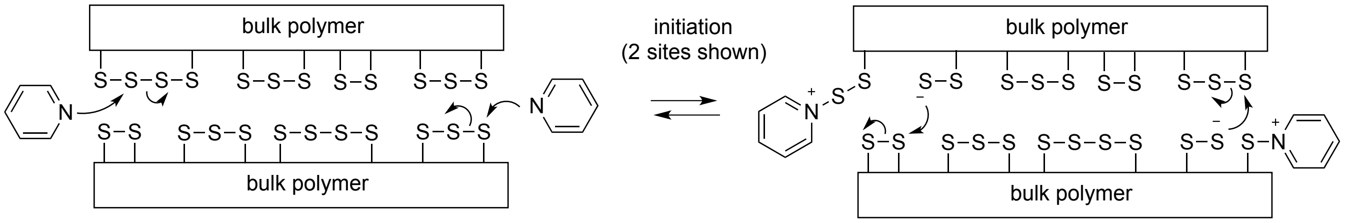

Pyridine will attack trisulfide or longer polysulfide groups on the polymer surface. Based on model crossover experiments and computational studies, pyridine is not expected to attack disulfide groups at 20 ºC.

Persulfide anion can attack other S-S bonds. Computations show this reaction has a low-lying barrier. S-S group attacked can be disulfide or higher sulfur rank. Attack can be interpolymer or intrapolymer.

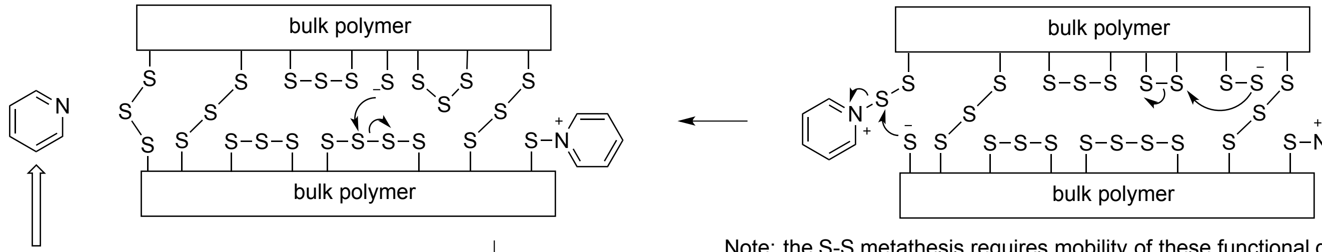

Regenerated pyridine catalyst can attack another polysulfide (re-initiating S-S metathesis). If pyridine evaporates, this is a termination step.

Justification: pyridine does not remain in polymer. Unmodified pyridine recovered from polymer.

Note: the S-S metathesis requires mobility of these functional groups for reaction to occur. This may require the polymer species at the interface to be above their glass transition temperature. The liquid pyridine at the interface may also facilitate this mass transfer through solvation of the reactive groups. However, control experiments indicate that neither inert solvents (such as chloroform, toluene, THF, etc) nor physical contact alone are sufficient for the polymers pieces to bond together. A reagent such as pyridine that breaks S-S bonds is required to join the polymer interfaces.

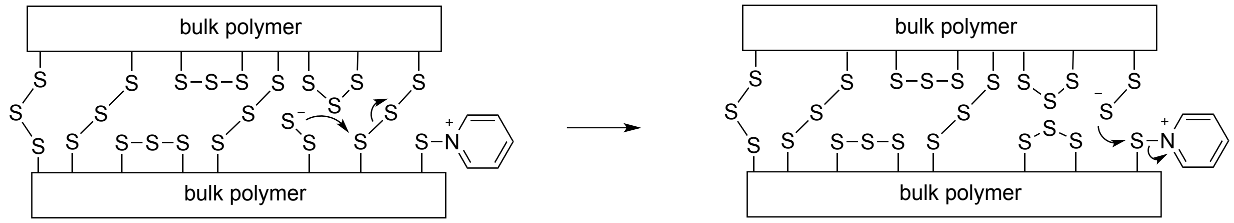

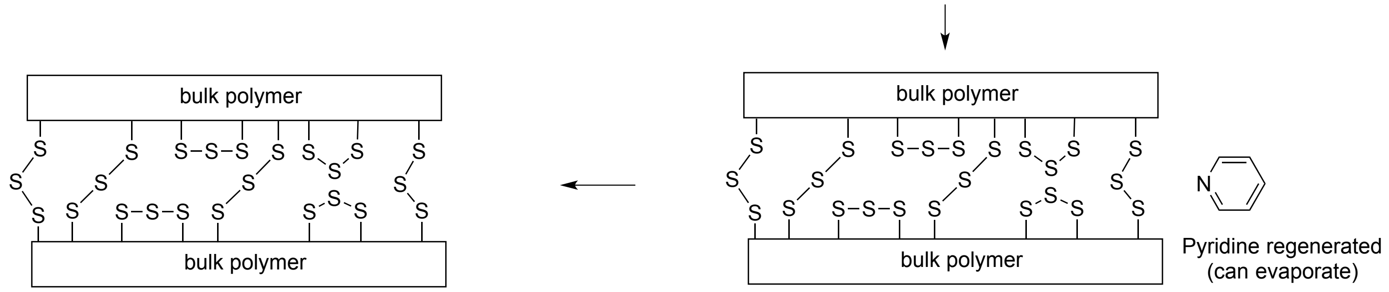

Reaction complete when all of the pyridine has evaporated. Two polymer interfaces joined through S-S linkages.

Shear and peel tests after using the polymer as a latent adhesive

The polymer was synthesized as before, with curing in a mold that provided a shape with two parallel square interfaces, one larger than the other. The smaller of the two surfaces had a side length of 40 mm for a total surface area of 1600 mm^2. The larger surface had a side length of approximately 45 mm for a total surface area of 2025 cm^2 This corresponds to a 200-fold increase in surface area over the dogbone shaped polymers which had a reactive surface of 2 mm x 4 mm. The pieces had a width of approximately 5 mm. The 40 mm x 40 mm surface area was used for the reactive interface.

Next, six 150 mm x 50 mm x 3 mm steel plates were obtained and four holes were then drilled into the corners of each piece. The centre of the holes were placed 15 mm from both sides in each corner of the metal plates with a diameter of 10 mm. A hammer drill was used to make each of the holes using a 3 mm drill bit as a guide, followed by a 10 mm drill bit to expand the hole to the desired size. A 50 mm x 50 mm area in the middle of one side of each plate was lightly ground using a hand grinder to roughen the surface. The larger square surface of the polymer pieces were glued to the roughened area of the steel plates using Gorilla Glue epoxy. A small amount of the epoxy was mixed and applied to the roughened area of the steel plate. The larger surface of a polymer piece was then placed on the epoxy glue and left for two hours. A ceramic tile was placed on the top of the polymer piece and clamped to the metal plate to ensure good contact between the polymer and the metal plate. This was repeated for two steel plates with two separate polymer pieces.

The catalysts were then placed on the smaller exposed surface of the polymer using a micropipette. 200 µL of tributylphosphine or pyridine was used because this volume covered the 1600 mm^2 polymer interface without overflowing. After the addition of the catalyst to the polymer surface, another metal plate with attached polymer was placed on top such that the polymer surfaces were aligned, and the metal plates were positioned perpendicular to each other. The polymer was then left for 24 hours to react before shear and peel tests were performed. Images of the polymer pieces and the metal plates are shown below:

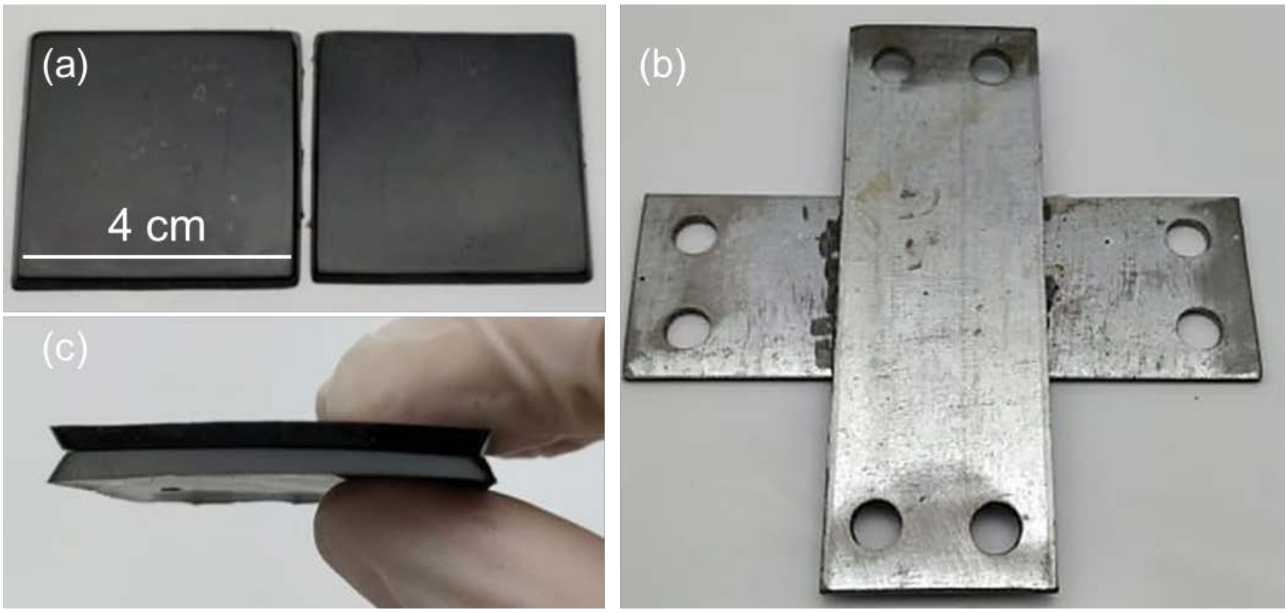

Shear force test

The shear force test was designed to pull each metal plate in opposite directions directly parallel to the adhered surface of the polymers. This was achieved by placing an 8 mm metal rod through each of the holes in one the metal plates. Nuts were used on both sides of the holes to prevent the plates from moving. An 8 mm steel quick connect chain link was then added to the top two holes of the other metal plate. A 1 m long, 5 mm thick steel chain was then hooked onto the quick connect chain link on one side, passed through a weight then hooked to the other quick connect chain link. The chain was doubled over to increase strength and decrease the total length to 50 cm. The weight was held by the adhesion for 30 seconds before removal. After which, the weight was removed and increased in mass. Intervals of 2.5 kg were used with a starting weight of 5 kg. The weight of the chain, two quick connect chain links and the steel plate was 739.3 g combined which was added to the mass of the weights to give the total weight. The greatest weight which was held for 30 seconds without failure was recorded as the maximum adhesion strength in this in-house test. After each test, the polymer and epoxy was removed from the metal using a handheld grinder. The experimental set up and tabulated data for the shear tests can be seen below.

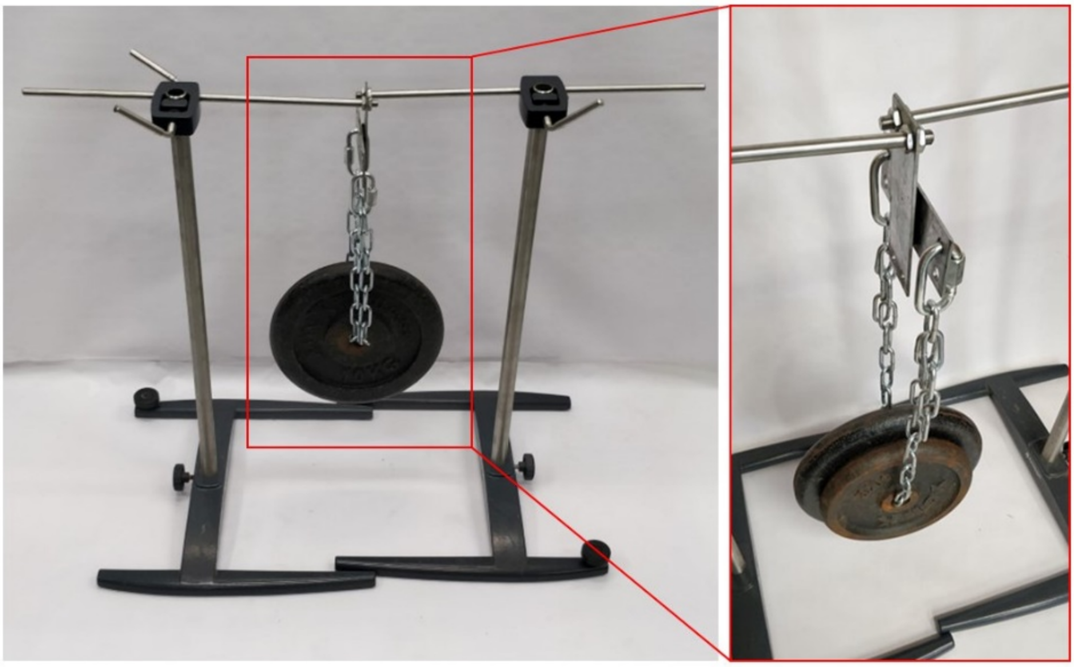

	Replica	*Maximum weight held for 30 seconds (kg)*	*Shear strength (N/cm^2)*	*Shear strength (psi)*	*Weight at failure (kg)*
Pyridine	1	20.74	12.7	180	23.24
	2	18.24	11.2	159	20.74
	3	18.24	11.2	159	20.74
	Average	19.07	11.7	166	21.57
Tributylphosphine	1	18.24	11.2	159	20.74
	2	18.24	11.2	159	20.74
	3	18.24	11.2	159	20.74
	Average	18.24	11.2	159	20.74

Peel test

The peel test was designed to test the force required to pull the polymer pieces apart with a force directed in the normal direction away from the reacted surface. The same metal plates were used and the reactions were performed in the same way as the shear test. A heavy retort stand was equipped with a bar which had a flattened steel section on the end. This flattened section had a width and length of 150 mm and a thickness of 3 mm. It had two 10 mm x 3 mm removed sections which ran parallel with the outer edges of the flattened section. The removed sections were located 22.5 mm from the outer edges of the flat section and were separated by 45 mm on the inner side.

Four, 8 mm steel quick connect chain links were hooked through each of the holes in both metal plates. A 1 m long, 5 mm thick steel chain was used to hold one of the metal plates with the attached polymer level by hooking the chain over the bar attached to the retort stand. Care was taken to ensure that the metal plate was level by using the same number of chain links on both sides of the metal plate, with any slack taken up by wrapping the chain around the center part of the flattened steel bar. Another chain was hooked onto the quick connect chain links on one side of the other metal plate and passed through a weight before being hooked to the quick connect chain links on the other side. Again, the chain was doubled up to increase strength and shorten the length. The weight was left for 30 seconds and if failure did not occur in this time, the weight was increased by 1.25 kg. An initial weight of 2.5 kg was used. The weight of four quick connect chain links, the chain and the metal plate was 878.2 g. This was added to the mass of the weight to give the total mass. The testing apparatus and results are shown below:

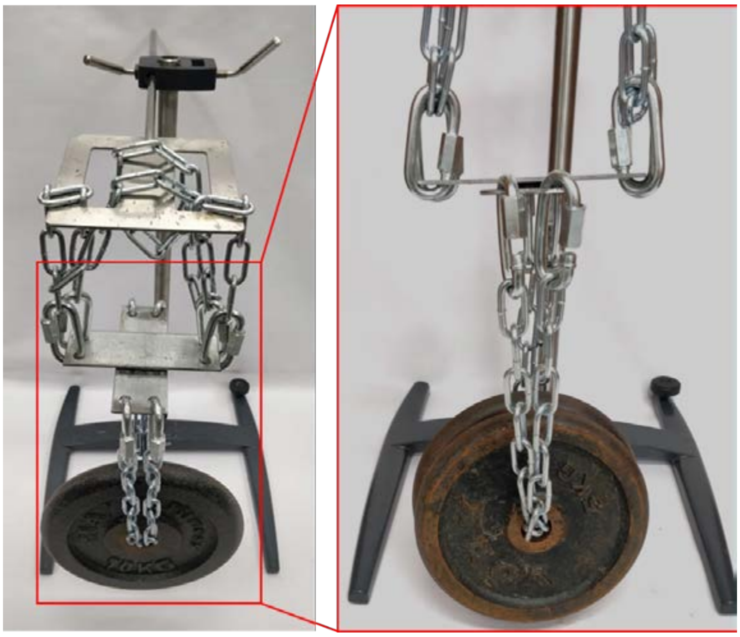

	Replica	*Maximum weight held for 30 seconds (kg)*	*Adhesion strength (kg/cm)*	*Weight at failure (kg)*
Pyridine	1	8.37	20.52	9.62
	2	10.87	26.64	12.12
	3	10.87	26.64	12.12
	Average	10.04	24.60	11.29
Tributylphosphine	1	5.87	14.38	7.12
	2	5.87	14.38	7.12
	3	8.37	20.52	9.62
	Average	6.70	16.43	7.95

Polymer assembly and additive manufacturing demonstration

The use of pyridine induced adhesion for polymer assembly was demonstrated by producing a wall of polymer bricks that were bound together using a pyridine as the “chemical mortar.” A mold negative was 3D printed using a Creality CR-10s pro 3D printer and standard poly(lactic acid) filament. The mold negative consisted of 10 rows of seven bricks. Six bricks in each row had dimensions of 10 mm x 10 mm x 16 mm while one brick in every row was made to be half the length, giving dimensions of 10 mm x 10 mm x 8 mm. This allowed the bricks to be overlapped like conventional brick laying technique to increase strength and give a classic break aesthetic. Each brick was separated by 5 mm and each row was separated by 5 mm. A wall was made that extended around the outside of the grid of bricks with a thickness of 3 mm and a height of 5 mm above the top of the bricks (15 mm total). Liquid silicone was mixed and poured into the mold negative up to the top of this wall and left for 2 hours at room temperature to cure. The mold negative and final mold can be seen below.

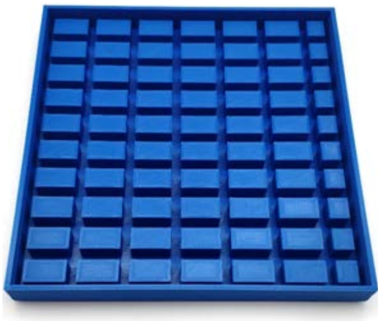

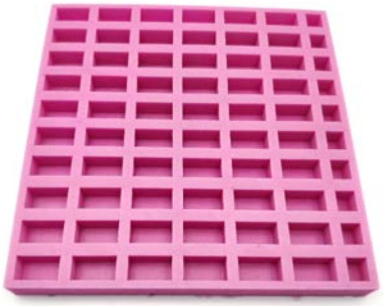

The polymer bricks were prepared using the same method as other experiments and maintained the same monomer ratio of 50 % sulfur, 35 % canola oil and 15 % DCPD. One 10 g batch could produce four full sized bricks and one half-brick. After some experimentation, it was found that the optimum volume of pyridine to induce the adhesion of the polymer bricks was 1 μL/4 mm^2. For the 10 mm x 10 mm side of the polymer, this corresponded to 25 μL while for the larger 10 mm x 16 mm side of the polymer, this corresponded to 40 μL of pyridine. The bottom layer of the wall was prepared first by applying 25 μL to the 10 mm x 10 mm side of a polymer brick and placing it in contact with the 10 mm x 10 mm side of another polymer brick. This was repeated for every brick on the bottom layer. Between layers, the wall was left for 30 minutes such that some adhesion had occurred before continuing to the next layer. For subsequent layers, 40 μL of pyridine was placed on the top of the previous layer for the 10 mm x 16 mm face and 25 μL of pyridine was applied to the 10 mm x 10 mm face of each brick. After all layers had been applied, the wall was left for 24 hours to ensure full adhesion of bricks. After this point, the wall could be picked up from any brick and remain intact.

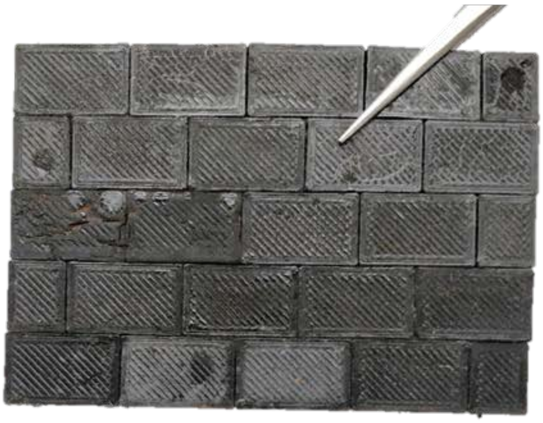

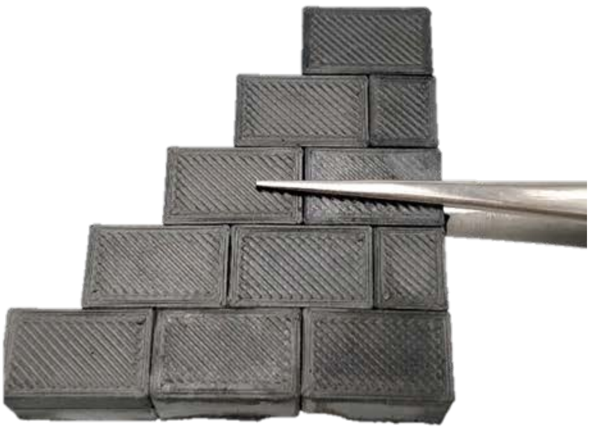

Repair of polymers using solution of pyridine in chloroform

Polymer pieces with a composition of 50 % sulfur, 35 % canola oil and 15 % DCPD (average sulfur rank of approximately 4) were cut down the centre of the gauge section with a scalpel. They were then returned to the mold and 10 μL of a pyridine solution in chloroform was applied to the interface. The polymer was left to react for 24 hours at room temperature. 6 controls were also prepared, 3 undamaged polymer pieces and 3 pieces that were cut with nothing applied at the interface. 10 pyridine solutions were prepared ranging from 10 % pyridine by volume to 100 % pyridine in 10 % increments. Each pyridine concentration was tested in triplicate. After reacting for 24 hours, the polymer samples were tested for tensile strength using dynamic mechanical analysis. The polymer pieces were clamped at both ends and a tensile force was ramped at 0.2 N/min until failure. The polymer pieces with no solvent did not repair. Polymer pieces to which chloroform only (no pyridine) was applied did not repair (see page S22-S24). In contrast, repair was observed with all solutions in which pyridine was present (see below). We note that the volatility of chloroform means that the concentration of pyridine was difficult to control throughout the repair process. The main purpose of this experiment was to show less pyridine can be used that when it is applied neat across the polymer surface.

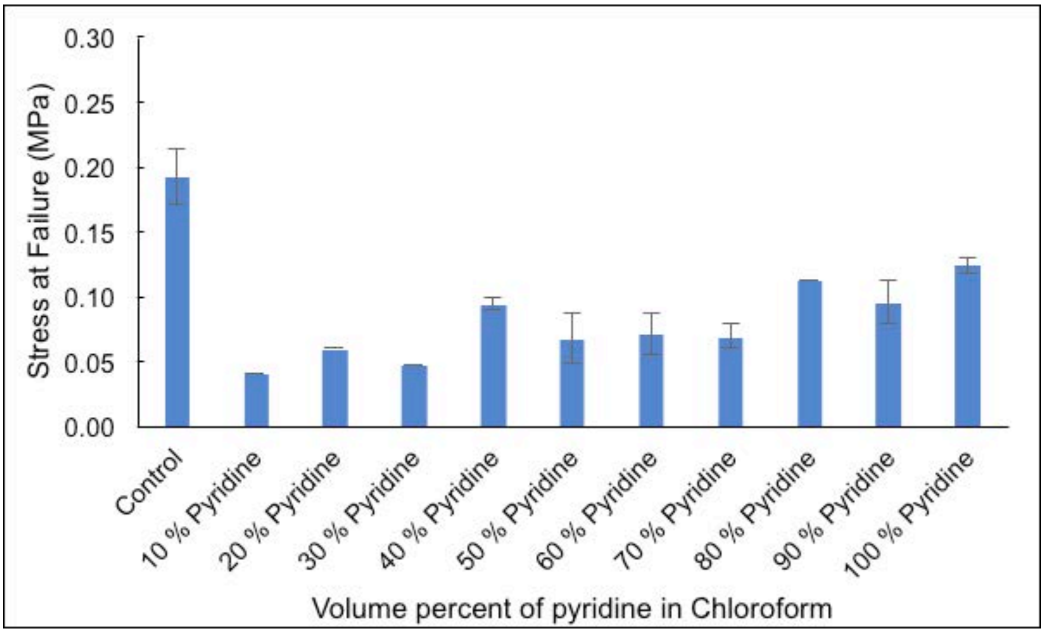

Polymer recycling and reforming using a pyridine catalyst

Pyridine was used as a catalyst to recycle and reform the polymer. Dog bone shaped polymer pieces were cut with a scalpel into small pieces before being ground in a mortar and pestle. The ground polymer powder was passed through a 1 mm sieve. Any pieces which were too large to fit through the sieve were ground again until all particles were able to pass through the sieve. 10 g of the ground polymer was weighed into a 100 mL beaker. This corresponds to approximately 8 dogbone pieces. 5 mL of pyridine was added to the polymer in 1 mL portions. After every addition of pyridine, the polymer was stirred with a small spatula for approximately 30 seconds. An 8.5 cm square press consisting of three parts, as shown below, was prepared in advance by fitting a Teflon sheet on the base piece. The Teflon sheet is to prevent the polymer from sticking to the metal press. The outer piece was then placed around the base piece and the Teflon sheet. The pyridine-coated polymer was transferred to the Teflon sheet and distributed evenly using the same spatula. Another Teflon sheet was placed on top of the polymer and the top piece of the press was fitted into the outer piece. The polymer was then compressed to 40 MPa for 30 minutes at room temperature. After compression, the polymer maintained the shape of the press, forming a flexible sheet. A graphic showing the full procedure can be seen below and on the following page.

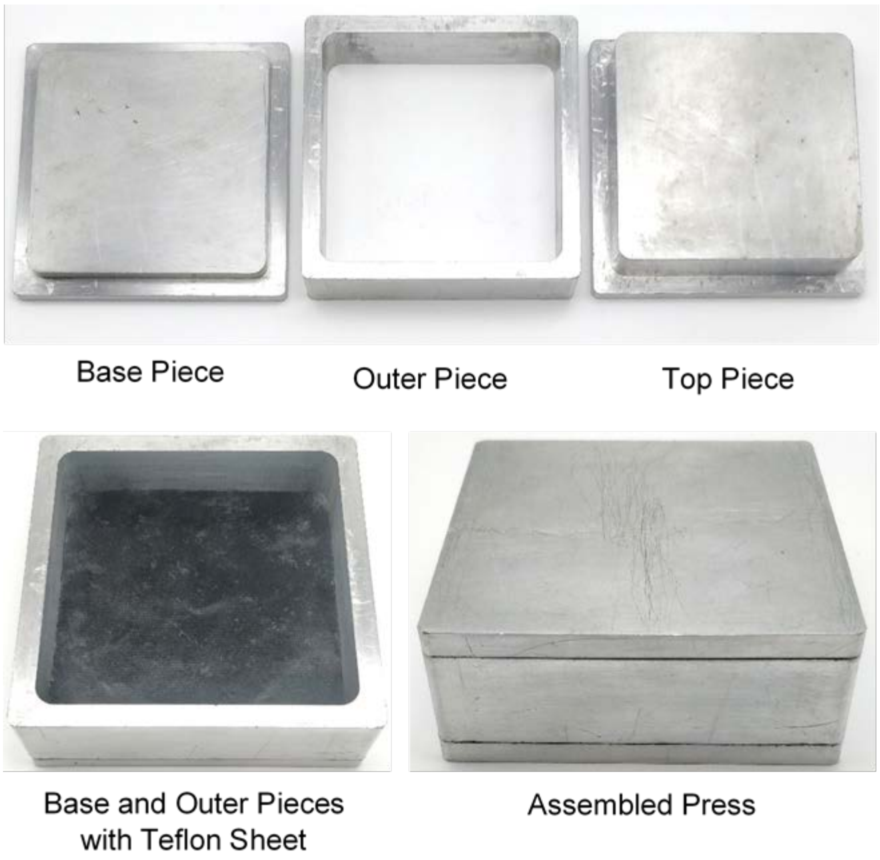

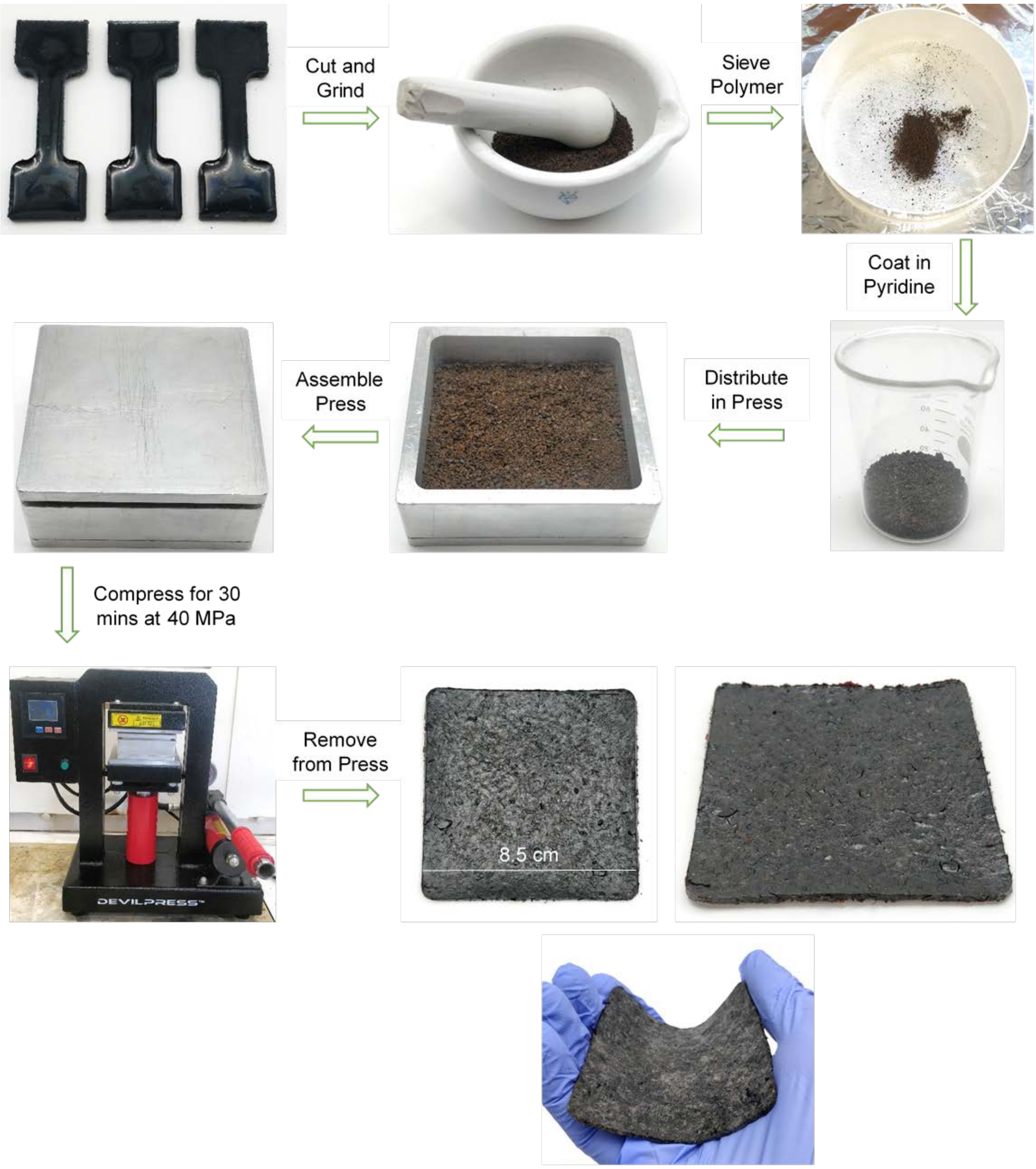
Cut and Grind
Sieve Polymer
Coat in Pyridine
Distribute in Press
Assemble Press
Compress for 30 mins at 40 MPa
DEVILPRESS
Remove from Press
8.5 cm

To demonstrate that the formation of the flexible mat was due to the pyridine induced adhesion and not due to pressure alone, a control was performed in which no pyridine was applied to the powdered polymer. 10 g of the polymer was weighed out and distributed evenly in the press with a Teflon sheet on either side. The polymer was then compressed to 40 MPa for 30 minutes with no pyridine. After 30 mins, the polymer was removed from the press. No adhesion occurred, with the polymer showing no change from before the pressure was applied.

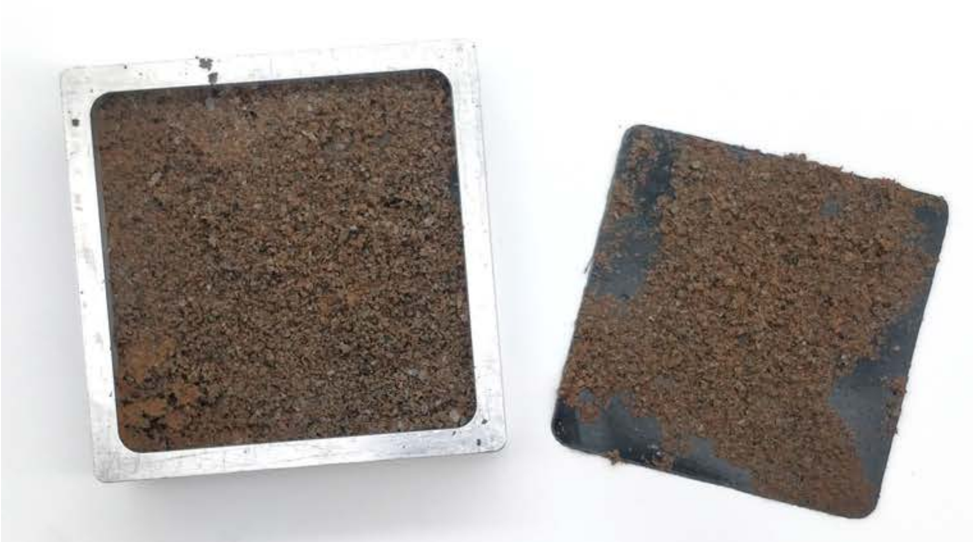

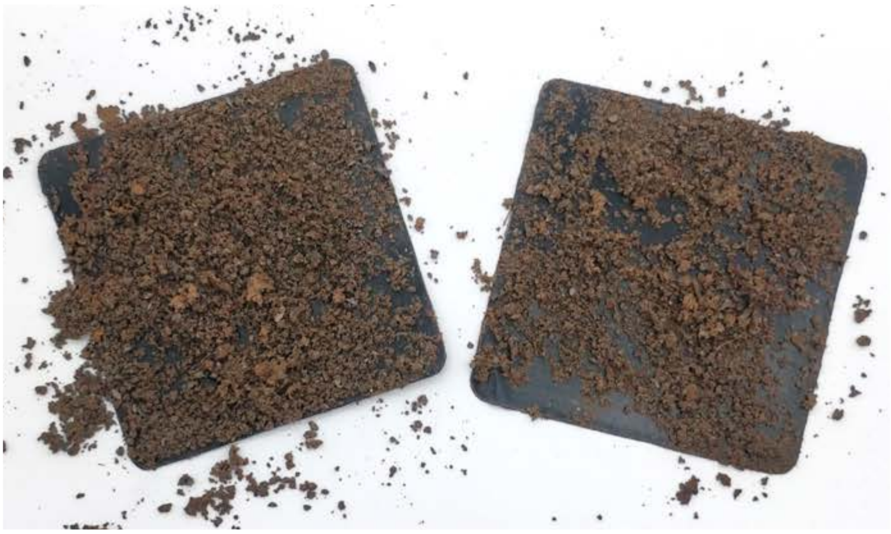

References

1) Tikoalu, A. D.; Lundquist, N. A.; Chalker, J. M. *Adv. Sustainable Syst.* **2020**, 1900111.

2) Kuttner, C.; Tebbe, M.; Schlaad, H.; Burgert, I.; and Fery, A. *ACS Appl. Mater. Interfaces* **2012**, *4*, 3484-3492.

3) Nikfarjam M.; López-Guerra E. A.; Solares, S. D.; Eslami B. *Beilstein J. Nanotechnol.* **2018**, *9*, 1116.

4) Lee C.; Yang, W.; Parr R. G. *Phys. Rev. B* **1988**, *37*, 785–789.

5) Becke, A. D. *J. Chem. Phys.* **1993**, *98*, 5648–5652.

6) Stephens, P.J.; Devlin, F.J.; Chabalowski, C. F.; Frisch, M. J. *J. Phys. Chem.* **1994**, *98,* 11623–11627.

7) Grimme, S. *WIREs Comput. Mol. Sci.* **2011**, *1*, 211.

8) Becke, A. D.; Johnson, E. R. J. *Chem. Phys.* **2005**, *123*, 154101.

9) Grimme, S.; Ehrlich, S; Goerigk, L. J. *Comput. Chem.* **2011**, *32*, 1456.

10) Marenich, A. V.; Cramer, C. J.; Truhlar, D. G. J. *Phys. Chem. B*, **2009**, *113*, 6378–6396.

11) Gonzalez, C.; Schlegel, H. B. *J. Chem. Phys.* **1989**, *90*, 2154.

12) Gaussian 16, Revision A.01, Frisch, M. J.; Trucks, G. W.; Schlegel, H. B.; Scuseria, G. E.; Robb, M. A.; Cheeseman, J. R.; Scalmani, G.; Barone, V.; Petersson, G. A.; Nakatsuji, H.; Li, X.; Caricato, M.; Marenich, A. V.; Bloino, J.; Janesko, B. G.; Gomperts, R.; Mennucci, B.; Hratchian, H. P.; Ortiz, J. V.; Izmaylov, A. F.; Sonnenberg, J. L.; Williams-Young, D.; Ding, F.; Lipparini, F.; Egidi, F.; Goings, J.; Peng, B.; Petrone, A.; Henderson, T.; Ranasinghe, D.; Zakrzewski, V. G.; Gao, J.; Rega, N.; Zheng, G.; Liang, W.; Hada, M.; Ehara, M.; Toyota, K.; Fukuda, R.; Hasegawa, J.; Ishida, M.; Nakajima, T.; Honda, Y.; Kitao, O.; Nakai, H.; Vreven, T.; Throssell, K.; Montgomery, J. A., Jr.; Peralta, J. E.; Ogliaro, F.; Bearpark, M. J.; Heyd, J. J.; Brothers, E. N.; Kudin, K. N.; Staroverov, V. N.; Keith, T. A.; Kobayashi, R.; Normand, J.; Raghavachari, K.; Rendell, A. P.; Burant, J. C.; Iyengar, S. S.; Tomasi, J.; Cossi, M.; Millam, J. M.; Klene, M.; Adamo, C.; Cammi, R.; Ochterski, J. W.; Martin, R. L.; Morokuma, K.; Farkas, O.; Foresman, J. B.; Fox, D. J. Gaussian, Inc., Wallingford CT, 2016. See also: http://www.gaussian.com.

13) Curtiss, L. A.; Redfern, P. C.; Raghavachari, K., *J. Chem. Phys.* **2007,** *127*, 124105.

14) Curtiss, L. A.; Redfern, P. C.; Raghavachari, K. *WIREs Comput. Mol. Sci.* **2011,** *1*, 810-825.

15) Curtiss, L. A.; Redfern, P. C.; Raghavachari, K. *Chem. Phys. Lett.* **2010,** *499*, 168-172.

16) Karton, A.; O'Reilly, R. J.; Radom, L. *J. Phys. Chem. A* **2012,** *116*, 4211–4221.

17) A. Karton, A.; Goerigk, L. *J. Comput. Chem.* **2015,** *36*, 622-632.

18) Yu, L. J.; Sarrami, F.; O'Reilly, R. J.; Karton, A. *Chem. Phys.* **2015,** *458*, 1-8.
